# Real-world impact of acupuncture on analgesics and healthcare resource utilization in breast cancer survivors with pain

**DOI:** 10.1186/s12916-024-03626-2

**Published:** 2024-09-16

**Authors:** Ding Quan Ng, Sanghoon Lee, Richard T. Lee, Yun Wang, Alexandre Chan

**Affiliations:** 1 School of Pharmacy & Pharmaceutical Sciences, University of California Irvine, 802 W Peltason Dr, Irvine, CA 92697-4625 USA; 2 School of Pharmacy, Chapman University, RK 94-206, 9401 Jeronimo Road, Irvine, CA 92618 USA; 3 College of Korean Medicine, Kyung Hee University, Seoul, South Korea; 4 Integrative Medicine Program, Departments of Supportive Care Medicine and Medical Oncology, City of Hope Comprehensive Cancer Center, Duarte, CA USA

**Keywords:** Acupuncture, Cancer pain, Real-world evidence, Claims data, Propensity score, Difference-in-difference

## Abstract

**Background:**

This study evaluated the real-world impact of acupuncture on analgesics and healthcare resource utilization among breast cancer survivors.

**Methods:**

From a United States (US) commercial claims database (25% random sample of IQVIA PharMetrics® Plus for Academics), we selected 18–63 years old malignant breast cancer survivors experiencing pain and ≥ 1 year removed from cancer diagnosis. Using the difference-in-difference technique, annualized changes in analgesics [prevalence, rates of short-term (< 30-day supply) and long-term (≥ 30-day supply) prescription fills] and healthcare resource utilization (healthcare costs, hospitalizations, and emergency department visits) were compared between acupuncture-treated and non-treated patients.

**Results:**

Among 495 (3%) acupuncture-treated patients (median age: 55 years, stage 4: 12%, average 2.5 years post cancer diagnosis), most had commercial health insurance (92%) and experiencing musculoskeletal pain (98%). Twenty-seven percent were receiving antidepressants and 3% completed ≥ 2 long-term prescription fills of opioids. Prevalence of opioid usage reduced from 29 to 19% (*P* < 0.001) and NSAID usage reduced from 21 to 14% (*P* = 0.001) post-acupuncture. The relative prevalence of opioid and NSAID use decreased by 20% (*P* < 0.05) and 19% (*P* = 0.07), respectively, in the acupuncture-treated group compared to non-treated patients (*n* = 16,129). However, the reductions were not statistically significant after adjustment for confounding. Patients receiving acupuncture for pain (*n* = 264, 53%) were found with a relative decrease by 47% and 49% (both *P* < 0.05) in short-term opioid and NSAID fills compared to those treated for other conditions. High-utilization patients (≥ 10 acupuncture sessions, *n* = 178, 36%) were observed with a significant reduction in total healthcare costs (*P* < 0.001) unlike low-utilization patients.

**Conclusions:**

Although adjusted results did not show that patients receiving acupuncture had better outcomes than non-treated patients, exploratory analyses revealed that patients treated specifically for pain used fewer analgesics and those with high acupuncture utilization incurred lower healthcare costs. Further studies are required to examine acupuncture effectiveness in real-world settings.

**Supplementary Information:**

The online version contains supplementary material available at 10.1186/s12916-024-03626-2.

## Background

Over two million patients are diagnosed with cancer annually in the United States (US), leading to a projected population of 22 million by 2030 [1]. Cancer patients experience poorer quality of life as a consequence of acute and chronic cancer-related symptoms [2, 3], among which pain is highly disabling and arguably the most feared symptom. Prevalence of pain stands at over 40% across cancer types [4, 5], which can increase to over 70% among metastatic cancer patients [4].

In comparison to nonmalignant pain, cancer pain is multifactorial with varying presentations dependent on the cancer type, stage, and cancer treatment received [6]. Aromatase inhibitors, prescribed for reducing breast cancer recurrence in > 50% of all breast cancer cases [7, 8], cause arthralgias in more than half of the patients [9, 10]. Taxane-related agents can induce neuropathic pain that is difficult to treat and debilitating to quality of life [11]. Although opioids and non-steroidal anti-inflammatory drugs (NSAIDs) remain highly relevant in pain management [12, 13], use of these medications is complicated by adverse events (AEs) such as constipation, gastric ulcers, and medication dependence. Optimizing pain control remains an uphill battle in cancer survivorship and supportive care [14–16]. With complex etiologies, significant AEs associated with analgesics use, and improved understanding of psychological and spiritual elements in cancer pain treatment, there has been a paradigm shift towards the integration of conventional pharmacological and complementary non-pharmacological interventions (i.e., integrative medicine) for holistic, safe, and effective pain management [12, 13].

In the recent SIO-ASCO guidelines for pain management, acupuncture received a moderate recommendation, highest among non-pharmacological therapies, for alleviating aromatase inhibitor-induced arthralgias, as well as general and musculoskeletal pain [12]. While published large-scale acupuncture trials have reported significant declines in cancer pain severity [17, 18], it remains unclear if these findings are translatable to real-world practices. The lack of a robust placebo control results in questions regarding the durability of the observed efficacy and criticisms about acupuncture’s identity as a “mega-placebo” [19, 20]. Alongside the dearth of scientific understanding regarding acupuncture among payers, acupuncture utilization is inequitable and largely restricted to patients with private insurance coverage and high disposable income for out-of-pocket expenditure [21]. Novel approaches for assessing the effectiveness and value of acupuncture are needed.

Using a large US commercial claims database, we evaluated the real-world treatment effect of acupuncture for managing cancer pain. Trial-reported benefits of acupuncture for cancer pain are most studied among breast cancer patients who thus serve as the target population for this study [17, 18, 22]. Pain outcomes were determined with analgesics utilization, and we hypothesized that acupuncture treatment had reduced the use of analgesics in comparison to non-treated survivors. The impact on direct medical costs and healthcare resource utilization were also evaluated to investigate the value of acupuncture from the payers’ perspective.

## Methods

### Data source

This was a retrospective cohort study utilizing a 25% random sample of IQVIA PharMetrics® Plus for Academics (JAN2006-DEC2021), a closed database of fully adjudicated patient-level health plan claims, providing a longitudinal view of inpatient and outpatient services, prescription and office/outpatient administered drugs, costs, and detailed enrollment information. Data contributors are largely commercial health plans, thus making the database representative of the commercially insured US national population for patients under 65 years of age. As data is de-identified in accordance with the US Health Insurance Portability and Accountability Act, institutional review board approval was not required for this study.

### Study design

**Fig. 1:**
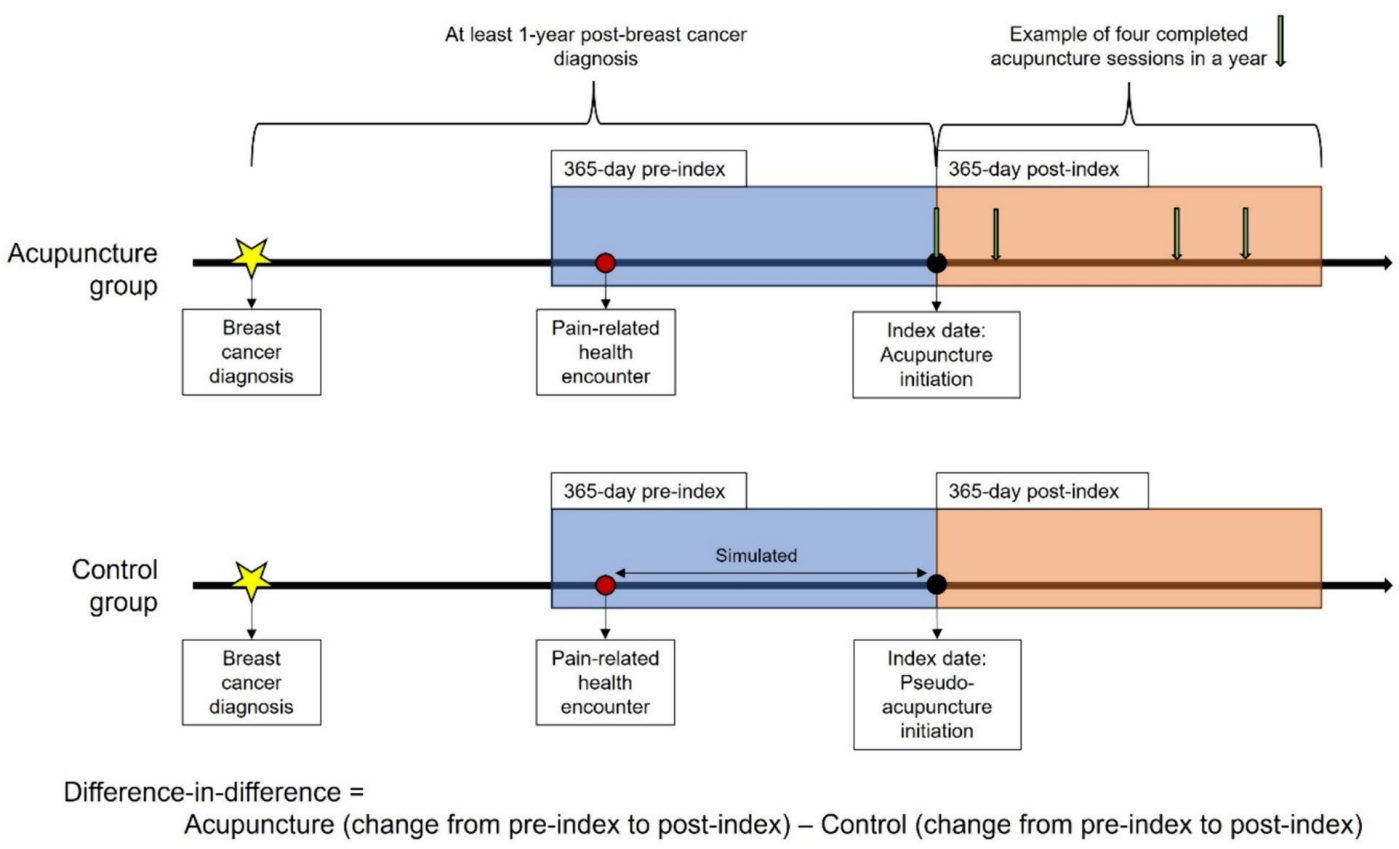
Illustration of study design and difference-in-difference methodology application

We employed the difference-in-difference (DID) technique, a two-timepoint, pre- and post-intervention analysis, to assess the causal effect of acupuncture exposure with appropriate counterfactual comparisons in a non-randomized setting [23]. In the acupuncture-treated group, the initiation of acupuncture was set as the reference date (i.e., index date) to determine the pre- and post-index periods for comparisons. The index date for non-treated controls was simulated following an observation in this study that patients seek acupuncture after an average of 236 days after a health encounter for pain. The key analysis involved comparing the annualized changes in outcomes, from the pre-index to post-index periods, between acupuncture-treated and non-treated cohorts (Fig. 1).

### Study population and eligibility criteria

Eligible patients were 18–63 years old (to exclude Medicare-switching), at least 1 year removed from malignant breast cancer diagnosis, continuously enrolled in medical and pharmacy plans for 2 years (from 365-day before to 365-day after index date), and with one or more documented musculoskeletal/general/neoplasm-related pain claims during the 365-day pre-index period. Eligible acupuncture-treated patients first received acupuncture after cancer diagnosis and were not found with acupuncture claims during the pre-index period, while non-treated patients must not have any documented acupuncture-related claims. The algorithms for identifying key health encounters (e.g., acupuncture, breast cancer, and pain) were summarized in Additional file 1: Table S1.

### Acupuncture exposure

Acupuncture exposure was determined by the presence of one or more claims for acupuncture using CPT-4 codes after cancer diagnosis (Additional file 1: Table 1). One service unit represents a 15-min treatment duration, and for the purpose of the analyses, an acupuncture session is considered as 30 min long [18].

### Outcomes

Analgesics of interest [opioids (non-parenteral), NSAIDs, and adjuvant analgesics such as serotonin-norepinephrine reuptake inhibitors (SNRIs), tricyclic antidepressants (TCAs), and gabapentinoids] were selected based on the NCCN clinical practice guidelines for adult cancer pain [13]. We implemented NDC and HCPCS codes to identify prescription fills (Additional file 1: Table S1). Measures of medication utilization include the prevalence of analgesics users and the rate of short-term (< 30-day supply) and long-term (≥ 30-day supply) prescription fills. The primary outcome is the annualized incremental change in the prevalence of opioid users when compared between acupuncture-treated and non-treated cohorts.

Direct medical costs were determined by the all-cause total healthcare costs paid by both patients and payers. Costs were adjusted to 2022 US dollars using the Consumer Price Index’s annual medical care component [24]. Healthcare resource utilization was defined as the rates of all-cause hospitalizations and emergency department (ED) visits.

### Covariates

Sociodemographic characteristics of age, gender, and US geographic region were measured at index date. Healthcare payer (commercial, managed Medicare, Medicare advantage/cost, others) and plan [preferred provider organization (PPO), health maintenance organization (HMO), point-of-service (POS), others] types were determined with monthly enrollment characteristics throughout the 2-year study period. Covariates evaluated during the 365-day pre-index period include total pain-related expenditure, pain subtypes (musculoskeletal, general, or neoplasm-related), Charlson’s comorbidity index excluding cancer-related codes as per National Cancer Institute (NCI) recommendations [25–27], menopausal symptoms, as well as predictors of severe patient-reported symptoms in cancer such as antidepressant use, depression health encounter, and ≥ 2 long-term opioid prescription fills [28]. Cancer-related characteristics (metastases and exposure to anticancer medications) were identified during the period between cancer diagnosis and index date.

### Statistical analysis

#### Primary analysis

Differences between covariates were tested between acupuncture-treated and non-treated patients using Wilcoxon rank-sum test and Pearson’s chi-squared test for continuous and categorical variables, respectively. Distribution of continuous outcomes (cost outcomes) was examined with histogram plots.

For DID analysis, the model was specified as follows: *g*(*Y*) = *β*0 + *β*1*[*post-index*] + *β*2*[*acupuncture group*] + *β*3*[*post-index* × *acupuncture group*] + *ε*, with *β*3 serving as the main coefficient of interest (i.e., DID estimate) for each outcome *Y*. Models for prevalence of analgesics use as outcomes were modeled with generalized estimating equations (GEE) for estimating population averaged effects, with logit (prevalence < 10%) or log (prevalence ≥ 10%) link function, Huber-White robust standard errors for controlling for heteroskedasticity, first-order autoregression correlation structure for time-varying outcomes, and binomial distribution. Similarly, with GEE and log link function, count outcomes were examined with negative binomial distribution, and cost data implemented gamma distribution. Changes in outcomes from pre- to post-index were estimated using the exponentials of *β*1 for non-acupuncture patients, and a linear combination of *β*1 and *β*3 for acupuncture patients. We further explored the bivariate relationship between predetermined covariates and outcomes that were found to be significant in unadjusted analyses to evaluate potential sources of significant differences not explained by acupuncture exposure.

For covariate adjustment (covariates are listed in Table 1), we implemented an inverse probability of treatment weighting (IPTW) approach as multivariable adjustment with the GEE-DID models could not converge for various outcomes [29]. The probability of treatment assignment (i.e., propensity scores), conditional on the covariates, was estimated with multivariable logistic regression [30]. We employed restricted cubic smoothing splines with five knots to model the relationship between continuous covariates and log-odds of the exposure [31]. Each case was weighted according to the inverse of the propensity score corresponding to its assigned exposure. We stabilized the weights by multiplying them with the marginal probability of the exposure assignment to correct for very large or small weights that could destabilize the estimated effect [31, 32]. Successful covariate balance between the two groups in the weighted cohort was defined as having standardized mean differences achieving <|0.1| for all covariates [33]. Covariate(s) that could not achieve the ideal balance standard were added to the GEE-DID model after IPTW-weighting.

**Table 1:** Baseline characteristics of study cohort

Characteristics	Controls	Acupuncture	*P* value
***N* (%)**	**16129 (97.0%)**	**495 (3.0%)**	
Year of index date, *n* (%)			0.010*
2007	1368 (8.5%)	58 (11.7%)	
2008	1903 (11.8%)	48 (9.7%)	
2009	1623 (10.1%)	57 (11.5%)	
2010	1504 (9.3%)	28 (5.7%)	
2011	1360 (8.4%)	39 (7.9%)	
2012	1309 (8.1%)	48 (9.7%)	
2013	1067 (6.6%)	32 (6.5%)	
2014	984 (6.1%)	25 (5.1%)	
2015	1145 (7.1%)	42 (8.5%)	
2016	1135 (7.0%)	40 (8.1%)	
2017	840 (5.2%)	21 (4.2%)	
2018	724 (4.5%)	19 (3.8%)	
2019	580 (3.6%)	27 (5.5%)	
2020	567 (3.5%)	11 (2.2%)	
2021	20 (0.1%)	0 (0.0%)	
Index age, median (Q1, Q3)	55 (50, 60)	55 (50, 59)	0.83
Age at breast cancer diagnosis, median (Q1, Q3)	53 (47, 57)	52 (47, 57)	0.11
Female, *n* (%)	15,977 (99.1%)	492 (99.4%)	0.73
US region, *n* (%)			< 0.001***
Northeast	3297 (20.4%)	88 (17.8%)	
Midwest	4760 (29.5%)	61 (12.3%)	
South	3996 (24.8%)	22 (4.4%)	
West	3669 (22.7%)	323 (65.3%)	
Payer type, *n* (%)			< 0001***
Commercial	13,927 (86.3%)	455 (91.9%)	
Managed Medicaid	481 (3.0%)	7 (1.4%)	
Medicare advantage or Medicare cost	458 (2.8%)	3 (0.6%)	
Others	1263 (7.8%)	30 (6.1%)	
Plan type, *n* (%)			< 0.001***
PPO	9683 (60.0%)	366 (73.9%)	
HMO	3411 (21.1%)	55 (11.1%)	
POS	770 (4.8%)	22 (4.4%)	
Others	2265 (14.0%)	52 (10.5%)	
Time from pre-index pain health encounter (in days), median (Q1, Q3)	252 (119, 336)	236 (105, 328)	0.14
Pain subtypes, *n* (%)			
Musculoskeletal	15,642 (97.0%)	486 (98.2%)	0.12
General	1188 (7.4%)	46 (9.3%)	0.11
Neoplasm-related	240 (1.5%)	5 (1.0%)	0.38
Annual healthcare cost (2022 USD), median (Q1, Q3)			
All claims	12,779.45 (5735.13, 34,230.06)	21,492.51 (9591.98, 62,439.71)	< 0.001***
Pain-related claims	457.58 (187.25, 1390.70)	902.03 (435.49, 2251.74)	< 0.001***
NCI Charlson’s comorbidity index, *n* (%)			0.28
0	10,439 (64.7%)	320 (64.6%)	
1	3761 (23.3%)	128 (25.9%)	
2	1227 (7.6%)	31 (6.3%)	
3 or more	702 (4.4%)	16 (3.2%)	
Cancer-related characteristics, *n* (%)			
Presence of metastases	1244 (7.7%)	57 (11.5%)	0.002**
Bone metastases	601 (3.7%)	21 (4.2%)	0.55
Brain metastases	133 (0.8%)	3 (0.6%)	0.59
Prior taxane exposure	3038 (18.8%)	122 (24.6%)	0.001**
Current or prior tamoxifen exposure	2143 (13.3%)	65 (13.1%)	0.92
Current or prior aromatase inhibitors exposure	2273 (14.1%)	75 (15.2%)	0.51
Supportive care-related characteristics, *n* (%)			
Current antidepressant use	3954 (24.5%)	131 (26.5%)	0.32
Depression	2739 (17.0%)	91 (18.4%)	0.41
Two or more 30-day supply of opioid prescription fills	659 (4.1%)	14 (2.8%)	0.16
Menopausal symptoms	2091 (13.0%)	133 (26.9%)	< 0.001***

Abbreviations: *HMO* Health maintenance organization, *N/n, counts,* *NCI* National Cancer Institute, *POS* Point-of-service, *PPO* Preferred provider organization, *Q1* Quartile 1, *Q3*, Quartile 3; *USD* United States Dollar

^*^P< 0.05, ^**^P< 0.01, ^***^P< 0.001

All analyses were two-tailed, tested at 5% significance level, and presented as ratios or percentage changes accompanying 95% confidence intervals (CI).

#### Sensitivity analysis

To emulate a randomized controlled trial design, sensitivity analysis comparing acupuncture-treated and non-treated cohorts utilized a 1:1 nearest neighbor matching without replacement. Propensity scores were estimated via logistic regression, adjusting for all covariates listed in Table 1.

#### Exploratory analysis

Because acupuncture treatment is highly heterogenous in terms of treated conditions [34, 35] and real-world utilization rate [36], we conducted exploratory analyses comparing changes in outcomes between (1) low (< 10 sessions) against high (≥ 10 sessions [18]) acupuncture utilization and (2) acupuncture treatment for pain against treatment for other conditions.

Data extraction, cleaning, IPTW, and propensity score matching (PSM, with R package MatchIt [37]) were executed with R version 4.3.2 [38]. All other analyses were performed on Stata version 16.1.

## Results

### Cohort characteristics

Our cohort comprised 16,624 eligible breast cancer survivors (median age = 55 years; stage 4 = 7.8%) who averaged 2.5 years (standard deviation, SD = 1.9) after cancer diagnosis. Four hundred ninety-five (3.0%) survivors had received acupuncture within a year of pain-related health encounter. Compared to non-treated survivors, more acupuncture-treated survivors had metastatic cancer, prior taxane chemotherapy exposure, reported more menopausal symptoms, lived in the Western region of the US, and with commercial PPO health plans. Acupuncture-treated patients were also with higher healthcare expenditure during the pre-index period (*P* < 0.05, Table 1). The selection algorithm for eligible patients is illustrated in Additional file 1: Fig. S1. Cost outcomes, both pre- and post-index, were found with a right-skewed distribution (Additional file 1: Fig. S2).

### Acupuncture utilization

Acupuncture-treated patients completed an average of 11.5 (median = 6) acupuncture sessions in a given year. The total annual cost of acupuncture per patient was $919.60 (median = $476.81), paying $183.22 (median = $49.12) out-of-pocket (OOP) on average. Cost per session averaged $84.80 (median = $78.56), with $22.39 (median = $10.17) being OOP expenditure (Additional file 1: Table S2).

### Impact of acupuncture on analgesics utilization

#### Opioid utilization

**Fig. 2:**
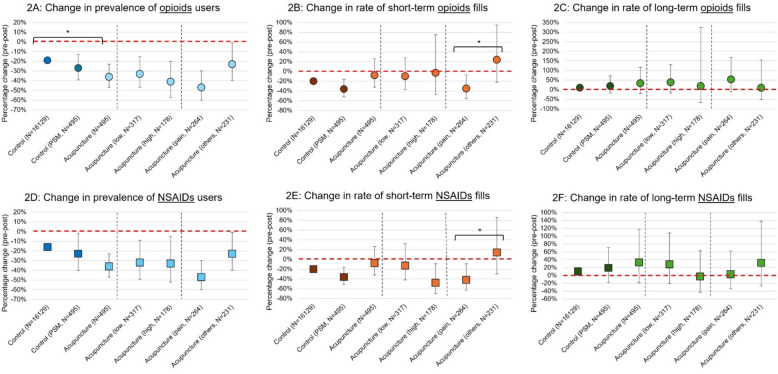
Percentage changes with 95% confidence intervals (pre-index to post-index) in opioid and NSAID utilization outcomes. The percentage change (pre-post) statistics, with 95% confidence intervals, were calculated from the pre-post change ratios from Tables 2 and 3. For example, a pre-post change ratio of 0.64 for opioid users among acupuncture-exposed patients (*n* = 495, Table 2), the percentage change (pre-post) statistics will be (0.64 − 1) × 100% =  − 36%. Absolute pre-index and post-index outcomes for the subgroups are presented in Table 2, eTable 6, and eTable 8. *P < 0.05 (DID estimate)

After acupuncture initiation, the prevalence of opioid users reduced by 36% (95% CI = 0.53 to 0.85, *P* < 0.01). The DID was estimated to have a prevalence ratio (PR) of 0.80 (95% CI = 0.66 to 0.96, *P* < 0.05), which indicated that the relative prevalence of opioid use decreased by 20% in the acupuncture-treated group compared to non-treated patients, over the 2-year, pre-index to post-index periods (Table 2, Fig. 2A). However, we did not observe statistically significant DID estimate on opioid prescription fill rates (Table 2, Fig. 2B–C). All predetermined covariates were significantly associated with opioid use in the bivariate analyses, except for biological sex, current or previous tamoxifen exposure, menopausal symptoms, and having two or more 30-day opioid prescription fills (Additional file 1: Table S3).

**Table 2:** Difference-in-difference analysis comparing changes in outcomes among acupuncture against controls

	Acupuncture (*N* = 495)			Controls, ref (*N* = 16129)			DID		Controls PSM, ref (*N* = 495)			DID
	Pre-index	Post-index	Pre-post change ratio^a^ (95% CI)	Pre-index	Post-index	Pre-post change ratio^b^ (95% CI)	Ratio^c^ (95% CI)	IPTW-ratio^d^ (95% CI)	Pre-index	Post-index	Pre-post change ratio^b^ (95% CI)	PSM-ratio^e^ (95% CI)
Proportion of users, *n* (%)												
Opioids	145 (29.3%)	93 (18.8%)	0.64*** (0.53, 0.77)	4191 (26.0%)	3381 (21.0%)	0.81*** (0.78, 0.83)	0.80* (0.66, 0.96)	0.88 (0.68, 1.15)	136 (27.5%)	99 (20.0%)	0.73** (0.61, 0.87)	0.88 (0.68, 1.14)
NSAIDs	103 (20.8%)	70 (14.1%)	0.68** (0.54, 0.85)	3100 (19.2%)	2605 (16.2%)	0.84*** (0.81, 0.87)	0.81 (0.65, 1.01)	0.87 (0.56, 1.34)	86 (17.4%)	66 (13.3%)	0.77* (0.60, 0.98)	0.89 (0.64, 1.23)
SNRIs	47 (9.5%)	48 (9.7%)	1.02 (0.79, 1.33)	1402 (8.7%)	1387 (8.6%)	0.99 (0.94, 1.04)	1.04 (0.79, 1.35)	0.80 (0.55, 1.17)	66 (13.3%)	56 (11.3%)	0.83 (0.66, 1.04)	1.23 (0.87, 1.75)
TCAs	18 (3.6%)	12 (2.4%)	0.66 (0.39, 1.10)	433 (2.7%)	423 (2.6%)	0.98 (0.89, 1.07)	0.67 (0.40, 1.14)	0.39 (0.12, 1.24)	13 (2.6%)	14 (2.8%)	1.08 (0.61, 1.92)	0.61 (0.28, 1.32)
Gabapentinoids	47 (9.5%)	65 (13.1%)	1.44** (1.10, 1.89)	1272 (7.9%)	1228 (7.6%)	0.96 (0.91, 1.02)	1.50** (1.14, 1.97)	1.53* (1.06, 2.23)	41 (8.3%)	41 (8.3%)	1.00 (0.73, 1.37)	1.44 (0.95, 2.18)
Short-term Rx fills, count (rate/person)												
Opioids	263 (0.53)	243 (0.49)	0.92 (0.68, 1.26)	9866 (0.61)	7922 (0.49)	0.80*** (0.77, 0.84)	1.15 (0.84, 1.58)	0.84 (0.51, 1.38)	329 (0.66)	209 (0.42)	0.64** (0.48, 0.84)	1.45 (0.96, 2.20)
NSAIDs	105 (0.21)	77 (0.16)	0.73 (0.52, 1.04)	2202 (0.14)	1706 (0.11)	0.77*** (0.71, 0.84)	0.95 (0.66, 1.36)	0.93 (0.59, 1.47)	68 (0.14)	60 (0.12)	0.88 (0.57, 1.38)	0.83 (0.47, 1.46)
SNRIs	9 (0.02)	10 (0.02)	1.11 (0.40, 3.06)	202 (0.01)	168 (0.01)	0.83 (0.60, 1.16)	1.34 (0.46, 3.88)	0.81 (0.29, 2.23)	10 (0.02)	2 (0.00)	0.20* (0.05, 0.82)	5.56 (0.98, 31.62)
TCAs	1 (0.00)	1 (0.00)	1.00 (0.06, 15.99)	85 (0.01)	73 (0.00)	0.86 (0.54, 1.35)	1.16 (0.07, 19.32)	1.43 (0.09, 23.79)	3 (0.01)	1 (0.00)	0.33 (0.02, 5.34)	3.00 (0.06, 151.49)
Gabapentinoids	10 (0.02)	4 (0.02)	0.40 (0.10, 1.58)	325 (0.02)	287 (0.01)	0.88 (0.67, 1.16)	0.45 (0.11, 1.83)	4.19 (0.61, 28.80)	21 (0.04)	17 (0.03)	0.81 (0.42, 1.56)	0.49 (0.11, 2.26)
Long-term Rx fills, count (rate/person)												
Opioids	82 (0.17)	109 (0.22)	1.33 (0.81, 2.17)	4694 (0.29)	5180 (0.32)	1.10** (1.04, 1.17)	1.20 (0.73, 1.98)	0.57 (0.15, 2.18)	85 (0.17)	101 (0.20)	1.19 (0.82, 1.72)	1.12 (0.60, 2.07)
NSAIDs	153 (0.31)	177 (0.36)	1.16 (0.80, 1.68)	5046 (0.31)	5022 (0.31)	1.00 (0.94, 1.05)	1.16 (0.80, 1.69)	1.32 (0.72, 2.41)	143 (0.29)	122 (0.25)	0.85 (0.61, 1.19)	1.36 (0.82, 2.24)
SNRIs	198 (0.40)	217 (0.44)	1.10 (0.85, 1.41)	7098 (0.44)	7170 (0.44)	1.01 (0.96, 1.06)	1.08 (0.84, 1.40)	0.97 (0.76, 1.25)	324 (0.65)	323 (0.65)	1.00 (0.80, 1.24)	1.10 (0.79, 1.54)
TCAs	70 (0.14)	47 (0.09)	0.67 (0.36, 1.25)	1677 (0.10)	1781 (0.11)	1.06 (0.96, 1.17)	0.63 (0.34, 1.19)	0.33 (0.10, 1.10)	67 (0.14)	52 (0.11)	0.78 (0.46, 1.30)	0.87 (0.38, 1.94)
Gabapentinoids	186 (0.38)	295 (0.60)	1.59** (1.21, 2.08)	4451 (0.28)	4841 (0.30)	1.09* (1.02, 1.16)	1.46** (1.10, 1.93)	1.21 (0.94, 1.55)	145 (0.29)	204 (0.41)	1.41 (0.70, 2.82)	1.13 (0.53, 2.38)
Healthcare resource utilization												
Total cost (2022 USD), median (Q1, Q3)	21,493 (9592, 62,440)	16,027 (7878, 33,534)	0.74** (0.61, 0.89)	12,779 (5735, 34,230)	9183 (4010, 21,805)	0.72*** (0.69, 0.74)	1.03 (0.85, 1.24)	1.29* (1.09, 1.52)	19,242 (9438, 58,526)	11,126 (4784, 26,870)	0.59*** (0.49, 0.71)	1.25 (0.96, 1.62)
Hospitalization, count (rate/person)	105 (0.21)	82 (0.17)	0.78 (0.56, 1.10)	2906 (0.18)	2232 (0.14)	0.77*** (0.72, 0.82)	1.02 (0.72, 1.44)	1.72* (1.04, 2.86)	122 (0.25)	71 (0.14)	0.58** (0.43, 0.79)	1.34 (0.85, 2.13)
Emergency department, count (rate/person)	206 (0.42)	175 (0.35)	0.85 (0.65, 1.11)	7307 (0.45)	5939 (0.37)	0.81*** (0.78, 0.85)	1.05 (0.80, 1.37)	1.29 (0.92, 1.80)	220 (0.44)	131 (0.26)	0.60*** (0.47, 0.76)	1.43 (0.99, 20.5)

Abbreviations: *CI*, confidence interval; *DID*, difference-in-difference; *GEE*, generalized estimating equation; *IPTW*, inverse probability of treatment weighting; *N/n*, counts; *NSAIDs*, non-steroidal anti-inflammatory drugs; *PSM*, propensity score matched; *Q1*, quartile 1; *Q3*, quartile 3; *ref*, reference group for DID analysis; *Rx*, prescription; *SNRIs*, serotonin-norepinephrine reuptake inhibitors; *TCAs*, tricyclic antidepressants; *USD*, United States dollar

^*^P < 0.05, ^**^P < 0.01, ^***^P < 0.001

^a^Exponential function of *β*1 + *β*3 in the GEE-DID model, log(*Y*) = *β*0 + *β*1*[*post-index*] + *β*2*[*acupuncture group*] + *β*3*[*post-index* × *acupuncture group*] + *ε*, where *Y* are either the prevalence or odds for binary outcomes, rate outcomes for prescription fills, hospitalization and emergency department visits, or cost outcomes

^b^Exponential function of *β*1 in the GEE-DID model

^c^Exponential function of *β*3 in the GEE-DID model

^d^Exponential function of *β*3 in the GEE-DID model after IPTW-weighting. Time from pre-index pain health encounter could not achieve a standardized mean difference <|0.1| after IPTW-weighting and was thus added into the models (eFig. 3)

^e^Exponential function of *β*3 in the GEE-DID model after PSM

The characteristics and covariate balance statistics of the IPTW-weighted and PSM-matched cohorts are summarized in eFig. 3 and Additional file 1: Table S4, respectively. After IPTW and PSM, the DID estimates on opioid use prevalence was not significantly different between treated and non-treated patients (Table 2, Fig. 2A–C).

#### NSAID utilization

Acupuncture treatment was associated with a 32% decline (95% CI = 0.54 to 0.85, *P* < 0.01) in the prevalence of NSAID users from baseline, with a DID PR estimate of 0.81, albeit not statistically significant (95% CI = 0.65 to 1.01, *P* > 0.05), compared to non-treated patients (Table 2, Fig. 2D). We did not observe any significant DID estimate on NSAID prescription fill rates or after IPTW and PSM (Table 2, Fig. 2E–F).

#### Adjuvant analgesics utilization

Acupuncture was not associated with any changes in TCA or SNRI utilization. However, significant increases in prevalence (odds ratio [OR] = 1.44, 95% CI = 1.10 to 1.89, *P* < 0.01) and long-term prescription fills (rate ratio [RR] = 1.59, 95% CI = 1.21 to 2.08, *P* < 0.01) of gabapentinoids were found among acupuncture-treated patients. This effect was associated with DID ratio estimate of 1.50 and 1.46, respectively, when compared to non-treated patients (*P* < 0.01). All predetermined covariates were significantly associated with gabapentinoid use, except biological sex and tamoxifen exposure. Age at breast cancer diagnosis was also significantly associated with rates of long-term prescription fills of gabapentinoids (Additional file 1: Table S3). DID remained statistically significant in the IPTW-weighted model but not after PSM when evaluating odds of gabapentinoid use (Table 2).

### Impact of acupuncture on healthcare cost and resource utilization

Breast cancer survivors who initiated acupuncture were found with an average decline in total healthcare cost by 26% (95% CI = 0.61 to 0.89, *P* < 0.01) and an absolute decrease by $15,513 (95% CI =  − $24,815 to − $6213, *P* < 0.01). This decrease was not significantly different from non-treated patients. However, DID estimates differ between IPTW and PSM analyses (Table 2).

### Exploratory analysis 1: high vs low acupuncture utilization

One hundred seventy-eight (36.0%) patients received ≥ 10 sessions and were characterized by a higher overall healthcare expenditure and increased prior taxane exposure at baseline (Additional file 1: Table S5).

The annualized changes in analgesics and healthcare resource utilization outcomes were not significantly different between low (< 10 sessions) and high (≥ 10 sessions) acupuncture utilization breast cancer survivors. Notably, a significantly larger increase in gabapentinoid utilization was observed among low-utilization patients (*P* < 0.01) but not among those with higher acupuncture utilization (Table 3, Additional file 1: Table S6).

**Table 3:** Difference-in-difference exploratory analysis by (1) high vs low acupuncture utilization and (2) acupuncture for pain vs other conditions

	Exploratory analysis 1: high vs low acupuncture utilization			Exploratory analysis 2: acupuncture for pain vs other conditions		
	Acupuncture, ≥ 10 sessions (*N* = 178)	Acupuncture, < 10 sessions (*N* = 317)	DID (ref: < 10 sessions)	Acupuncture for pain (*N* = 264)	Acupuncture for other conditions (*N* = 231)	DID (ref: other conditions)
	Pre-post change ratio^a^ (95% CI)	Pre-post change ratio^b^ (95% CI)	Ratio^c^ (95% CI)	Pre-post change ratio^a^ (95% CI)	Pre-post change ratio^b^ (95% CI)	Ratio^c^ (95% CI)
Proportion of users, PR/OR						
Opioids	0.59** (0.43, 0.80)	0.67** (0.53, 0.85)	0.88 (0.59, 1.30)	0.53*** (0.40, 0.70)	0.77* (0.60, 0.99)	0.69 (0.47, 1.00)
NSAIDs	0.67* (0.48, 0.95)	0.68* (0.51, 0.91)	0.98 (0.63, 1.55)	0.63** (0.47, 0.85)	0.74 (0.53, 1.05)	0.85 (0.54, 1.34)
SNRIs	0.79 (0.49, 1.26)	1.14 (0.83, 1.57)	0.69 (0.39, 1.22)	1.11 (0.74, 1.66)	0.96 (0.68, 1.35)	1.16 (0.68, 1.97)
TCAs	0.74 (0.36, 1.52)	0.59 (0.28, 1.23)	1.25 (0.45, 3.50)	1.00 (0.56, 1.78)	0.24* (0.07, 0.82)	4.11* (1.07, 15.72)
Gabapentinoids	1.17 (0.79, 1.72)	1.66** (1.15, 2.41)	0.70 (0.41, 1.20)	1.86** (1.26, 2.75)	1.13 (0.77, 1.64)	1.65 (0.96, 2.84)
Short-term Rx fills, RR						
Opioids	0.97 (0.53, 1.75)	0.90 (0.63, 1.28)	1.08 (0.54, 2.14)	0.65* (0.45, 0.93)	1.24 (0.78, 1.95)	0.53* (0.29, 0.94)
NSAIDs	0.52* (0.30, 0.91)	0.87 (0.58, 1.32)	0.60 (0.30, 1.20)	0.58* (0.37, 0.91)	1.14 (0.70, 1.86)	0.51* (0.26, 0.99)
SNRIs	0.50 (0.03, 8.00)	1.29 (0.42, 3.91)	0.39 (0.02, 7.73)	0.33 (0.03, 3.85)	1.50 (0.44, 5.14)	0.22 (0.01, 3.44)
TCAs^d^	–	–	–	–	–	–
Gabapentinoids^d^	–	–	–	0.20 (0.02, 2.54)	0.60 (0.13, 2.79)	0.33 (0.02, 6.50)
Long-term Rx fills, RR						
Opioids	1.19 (0.33, 4.25)	1.38 (0.83, 2.30)	0.86 (0.22, 3.41)	1.53 (0.88, 2.68)	1.10 (0.48, 2.55)	1.39 (0.51, 3.81)
NSAIDs	0.97 (0.57, 1.63)	1.28 (0.79, 2.08)	0.76 (0.37, 1.54)	1.03 (0.66, 1.62)	1.32 (0.73, 2.38)	0.78 (0.37, 1.64)
SNRIs	0.84 (0.57, 1.23)	1.20 (0.88, 1.63)	0.70 (0.43, 1.14)	1.37 (0.86, 2.17)	0.93 (0.72, 1.19)	1.48 (0.87, 2.50)
TCAs	0.44 (0.18, 1.08)	0.91 (0.41, 2.03)	0.49 (0.15, 1.61)	0.78 (0.39, 1.55)	0.27 (0.06, 1.23)	2.93 (0.55, 15.70)
Gabapentinoids	1.31 (0.86, 2.01)	1.88*** (1.35, 2.61)	0.70 (0.41, 1.20)	1.99** (1.19, 3.31)	1.33 (0.99, 1.80)	1.49 (0.82, 2.69)
Healthcare resource utilization						
Total cost (2022 USD), cost change ratio	0.59*** (0.46, 0.76)	0.82 (0.64, 1.06)	0.72 (0.50, 1.02)	0.67*** (0.54, 0.82)	0.79 (0.59, 1.06)	0.85 (0.59, 1.21)
Hospitalization, RR	0.56 (0.30, 1.04)	0.92 (0.61, 1.38)	0.61 (0.29, 1.28)	0.72 (0.41, 1.26)	0.83 (0.54, 1.27)	0.86 (0.43, 1.75)
Emergency department, RR	0.77 (0.52, 1.14)	0.93 (0.64, 1.33)	0.83 (0.48, 1.41)	0.84 (0.57, 1.23)	0.86 (0.59, 1.25)	0.97 (0.57, 1.66)

*Abbreviations*: *CI*, confidence interval; *DID*, difference-in-difference; *GEE*, generalized estimating equation; *N/n*, counts; *NSAIDs*, non-steroidal anti-inflammatory drugs; *OR*, odds ratio; *PR*; prevalence ratio; *ref*, reference group for DID analysis; *RR*, rate ratio; *Rx*, prescription; *SNRIs*, serotonin-norepinephrine reuptake inhibitors; *TCAs*, tricyclic antidepressants; *USD*, United States dollar

^*^P < 0.05, ^**^P < 0.01, ^***^P < 0.001

^a^Exponential function of *β*1 + *β*3 in the GEE-DID model, log(*Y*) = *β*0 + *β*1*[*post-index*] + *β*2*[*group*] + *β*3*[*post-index* × *group*] + *ε*, where *Y* are either the prevalence or odds for binary outcomes, rate outcomes for prescription fills, hospitalization and emergency department visits, or cost outcomes

^b^Exponential function of *β*1 in the GEE-DID model

^c^Exponential function of *β*3 in the GEE-DID model

^d^Models did not converge if point estimate and 95% CI is not estimated

High-utilization patients, despite spending more on acupuncture sessions (average total cost: $1035 vs $787, *P* = 0.054), were observed with a significant reduction in total healthcare costs after acupuncture treatment (*P* < 0.001) unlike low-utilization patients (Table 3, Additional file 1: Table S6). After further stratifying into quintiles based on number of completed acupuncture sessions, the average cost savings in quintiles 1, 2, 4, and 5 were comparable to the PSM-control cohort, except for quintile 3 which comprised patients who completed 5 to 8.5 sessions (Additional file 1: Fig. S4).

### Exploratory analysis 2: acupuncture for pain vs other conditions

Two hundred sixty-four (53.3%) patients received acupuncture specifically for pain and, compared to patients treated for other conditions (*n* = 231), were observed with less neoplasm-related pain diagnoses and more acupuncture sessions (Additional file 1: Table S7).

Patients receiving acupuncture for pain were found with statistically significant DID RR estimates of 0.53 (95% CI = 0.29 to 0.94, *P* < 0.05) and 0.51 (95% CI = 0.26 to 0.99, *P* < 0.05), suggesting a decline in short-term opioid and NSAID prescription fills compared to patients using acupuncture for treating other conditions (Table 3, Fig. 2B and F). These DID estimates remained statistically significant after adjusting for neoplasm-related pain diagnoses (*P* < 0.05, results not presented). On the other hand, patients treated for non-pain conditions were observed with a significantly larger decline in prevalence of TCA users compared to those treated for pain (*P* < 0.05, Table 3, Additional file 1: Table S8). We also observed a statistically significant increase in gabapentinoid utilization (*P* < 0.01) and reduction in total healthcare cost (*P* < 0.001) only among patients treated for pain (Table 3, Additional file 1: Table S8). Further stratified analysis found that gabapentinoid utilization was significantly increased among low-utilization pain-treated survivors (*P* < 0.05) rather than high-utilization patients (*P* > 0.05) (Additional file 1: Table S9).

##  Discussion

This is the first study that has evaluated the real-world impact of acupuncture treatment for cancer pain. The overall acupuncture utilization for pain is low (< 3%) among commercially insured breast cancer survivors, albeit comparable to previous published data [39]. Across all analyzed subgroups of patients, those treated specifically for pain were found with the largest decline in prevalence and short-term fills of opioids and NSAIDs after acupuncture treatment. High-utilization patients were found with greater healthcare cost savings compared to low-utilization patients, with the latter escalating to the use of gabapentinoids. Taken together, findings confirm that acupuncture reduces pain and that a value-based care model may be most appropriate to facilitate increased acupuncture coverage for pain management among cancer survivors.

Acupuncture analgesia has been widely recognized in multiple clinical guidelines in both cancer and non-cancer patient populations [12, 40, 41] and functionally ascribed to local and systemic neurochemical changes. The role of endogenous opiates in acupuncture analgesia was evident from the lack of analgesic response after naloxone administration and opiate receptors depletion [42, 43]. Local release of adenosines and the activation of adenosine A1 receptors, which serve as inhibitory mediators for neurons, are crucial in inhibiting the transmission of painful stimuli to the anterior cingulate cortex during acupuncture [44]. Clinical evidence for acupuncture in cancer pain is aplenty, with two large, randomized trials specifically described in the SIO-ASCO integrative oncology guidelines for pain management [12]. The PEACE study (*n* = 360) investigated the efficacy of electroacupuncture and auricular acupuncture compared to waitlist control reported significant pain reduction after 10 weekly acupuncture sessions [18]. Another trial recruited breast cancer patients (*n* = 226) experiencing aromatase inhibitor-induced joint pain and found that 12 weeks (18 sessions) of acupuncture had relieved pain compared to sham and waitlist controls [17]. Our study contributed by finding a decline in opioid and NSAID real-world medication use between the year before and after acupuncture initiation. Importantly, this improvement is not attributable to non-specific effects of acupuncture as evidenced by the lack of effectiveness observed among patients treated for non-pain conditions. In all, acupuncture is not just a placebo as it has achieved a clinically meaningful impact on patients’ health in the real-world.

Some pain-treated, low-utilization patients were observed with increased gabapentinoid usage. Considering the relative safety of acupuncture across indications [45], we hypothesize that gabapentinoid initiation is confined to non-responders of acupuncture. Relatedly, our assessment of total healthcare expenditure concluded that cost savings after acupuncture initiation were comparable to non-treated controls, other than one-fifth of treated patients who were not frequent users of acupuncture and likely non-responders. Thus, future research should examine ways to predict acupuncture response prior to treatment initiation. Applying the concept of precision medicine, the research into clinical, sociodemographic, environmental, lifestyle, and genetic predictors of treatment response will enable the prescription of acupuncture to patients who will receive maximal health benefits [46]. Adopting a value-based care model by periodically evaluating patient response to acupuncture (e.g., monitoring patient-reported outcomes and ensuring patient adherence) will ensure that only responders should continue with the treatment and prevent unnecessary healthcare expenditure [47]. Furthermore, it is known that acupuncture utilization was more frequent among individuals of female gender, White or Asian racial-ethnic background, and those with higher levels of education and income due to affordability-related barriers [21, 48, 49]. Despite the promises of acupuncture for cancer pain management, the inequitable access to acupuncture for minoritized and marginalized populations will pervade the widening care disparities [21, 50]. Other payers, including Medicaid, Medicare, and HMOs (referring to Additional file 1: Table S3, these payers were observed with higher prescription rates of analgesics), should consider implementing precision medicine and value-based care to enhance cancer survivors’ access to acupuncture treatment for pain and reduce healthcare disparities.

There are several limitations in this study. As with other retrospective studies, unmeasured confounding is unavoidable. Although acupuncture is recommended for all cancer survivors, we have focused only breast cancer survivors to minimize the degree of unmeasured confounding; future studies should investigate in another group of cancer survivors. Even so, not all sociodemographic variables are available, thus we could not adjust for factors such as race/ethnicity and education attainment that are different between acupuncture-treated and non-treated patients. Nevertheless, while these characteristics may impact acupuncture access and utilization, they are unlikely to impact the underlying efficacy [50]. Selection bias is another concern. Naturally, cancer patients seeking integrative therapies such as acupuncture have greater symptom burden and poorer symptom control than their peers [51–53]. While we have attempted to control for potential confounders such as antidepressants and chronic opioid use, our PSM-control cohort is observed with greater improvements in ED and hospitalization rates compared to other control and acupuncture-treated patients. Non-pharmacological modalities for pain relief are seldom captured in claims dataset, in vie that they are often out-of-pocket expenditures [39]. Patients seeking these modalities are likely to share similar characteristics to the patients within the acupuncture-treated cohort in our study, thus possibly explaining the lack of associations observed in our analyses after IPTW and PSM. Acupuncture utilization was also highly heterogenous, with only 53% receiving it for pain management. We reason that these limitations have blunted the reported association, and the true effect size may be greater.

Finally, our statistical power and study design are impacted by the low real-world utilization of reimbursed acupuncture treatment. In Medicare Part B, beginning in 2020, acupuncture is claimable for only chronic low back pain [54]. Claims for other pain-related indications can only be obtained from Medicare managed care (i.e., Medicare Part C). The consequence is that SEER-Medicare, the database of choice for cancer-specific questions among older adults, is not suitable as the reliability and completeness of Medicare Part C data remains an area of active research [55, 56]. It is prudent to apply this methodology on SEER-Medicare after data validations to address the effectiveness of acupuncture coverage in older cancer survivors. Regardless, the study’s strength is underscored by its large sample size of over 10,000 patients and a robust pre-post-intervention, DID study design that ensures exposure-outcome temporality and similar index times for comparison of outcomes between acupuncture and control patients, effectively answering an important research question regarding the real-world treatment effect of acupuncture at managing pain in breast cancer survivors.

## Conclusion

Although adjusted results did not show that patients receiving acupuncture had better outcomes than non-treated patients, exploratory analyses revealed that patients treated specifically for pain used fewer analgesics and those with high acupuncture utilization incurred lower healthcare costs. Follow-up research should evaluate ways to examine acupuncture effectiveness in real-world settings with pragmatic trials, understand predictors of acupuncture response, validate our findings within SEER-Medicare, and replicate with other groups of cancer survivors (e.g., bone metastases). Clinicians and payers can consider applying value-based care to enhance cancer survivors’ access to acupuncture treatment for pain.

## Supplementary Information

Supplementary Material 1: Additional file 1: Table S1 Algorithms for identifying health-related covariates. Table S2 Acupuncture utilization statistics. Table S3 Bivariate association analysis between predetermined covariates and outcomes with significant DID estimates when comparing acupuncture-treated vs non-treated breast cancer survivors. Table S4 Baseline characteristics comparing acupuncture against control patients after propensity score matching. Table S5 Baseline characteristics of high vs low acupuncture utilization patients. Table S6 Difference-in-difference exploratory analysis by high vs low acupuncture utilization. Table S7 Baseline characteristics comparing acupuncture-treated patients for pain vs for other conditions. Table S8 Difference-in-difference exploratory analysis by acupuncture for pain vs other conditions. Table S9 Change in gabapentinoid utilization and total healthcare cost among patients treated with acupuncture for pain, stratified by high vs low acupuncture utilization. Fig. S1 Acupuncture and control cohort selection. Fig. S2 Distribution of annualized total healthcare cost, pre- and post-index. Fig. S3 Standardized mean differences in the unadjusted (original) and weighted (IPTW-weighted) cohorts. Fig. S4 Effect of number of acupuncture sessions (in quintiles) on the change in total all-cause healthcare cost from pre- to post-index.

## Data Availability

The dataset was available for purchase from IQVIA. We do not own the data and hence are not permitted to share in the original form. The underlying code for this study is not publicly available but may be made available to qualified researchers on reasonable request from the corresponding authors.

## Data availability

No datasets were generated or analysed during the current study.
