# Supplementary material for: Real-world impact of acupuncture on analgesics and healthcare resource utilization in breast cancer survivors with pain
Source: BMC Med. 2024 Sep 16;22:394. doi: 10.1186/s12916-024-03626-2 (PMC11406884; doi:10.1186/s12916-024-03626-2)

**Additional File 1**

DQ Ng, S Lee, RT Lee, Y Wang, A Chan. Real-world impact of acupuncture on analgesics and healthcare resource utilization in breast cancer survivors with pain.

**Table S1: Algorithms for identifying health-related covariates**

**Table S2: Acupuncture utilization statistics**

**Table S3: Bivariate association analysis between predetermined covariates and outcomes with significant DID estimates when comparing acupuncture-treated vs non-treated breast cancer survivors**

**Table S4: Baseline characteristics comparing acupuncture against control patients after propensity score matching**

**Table S5: Baseline characteristics of high vs low acupuncture utilization patients**

**Table S6: Difference-in-difference exploratory analysis by high vs low acupuncture utilization**

**Table S7: Baseline characteristics comparing acupuncture-treated patients for pain vs for other conditions**

**Table S8: Difference-in-difference exploratory analysis by acupuncture for pain vs other conditions**

**Table S9: Change in gabapentinoids utilization and total healthcare cost among patients treated with acupuncture for pain, stratified by high vs low acupuncture utilization**

**Fig. S1: Acupuncture and control cohort selection**

**Fig. S2: Distribution of annualized total healthcare cost, pre- and post-index**

**Fig. S3: Standardized mean differences in the unadjusted (original) and weighted (IPTW-weighted) cohorts**

**Fig. S4: Effect of number of acupuncture sessions (in quintiles) on the change in total all-cause healthcare cost from pre- to post-index**

**Table S1: Algorithms for identifying health-related covariates**

| **Covariate** | **Algorithms** |
| --- | --- |
| Acupuncture | CPT-4:   - 97810: Acupuncture, one or more needles; without electrical stimulation, initial 15 minutes of personal one-on-one contact with the patient. - 97811: Acupuncture, one or more needles; without electrical stimulation, each additional 15 minutes of personal one-on-one contact with the patient, with re-insertion of needles. - 97813: Acupuncture, one or more needles; with electrical stimulation, initial 15 minutes of personal one-on-one contact with the patient. - 97814: Acupuncture, one or more needles; with electrical stimulation, each additional 15 minutes of personal one-on-one contact with the patient, with re-insertion of needles. |
| Cancer (malignant neoplasms) | ICD-9-CM: 140-209  ICD-10-CM: C00-C96 |
| Malignant breast cancer | ICD-9-CM: 174.x, 175.x  ICD-10-CM: C50.x |
| Metastases | ICD-9-CM: 197.x, 198.x, 199.0, 209.7x  ICD-10-CM: C7B.x, C78.x, C79.x, C80.0 |
| Brain metastases | ICD-9-CM: 198.3  ICD-10-CM: C79.3x |
| Bone metastases | ICD-9-CM: 198.5  ICD-10-CM: C79.5x |
| Neoplasm-related pain | ICD-9-CM: 338.3  ICD-10-CM: G89.3 |
| General pain | ICD-9-CM: 338.19, 338.29, 338.4, 780.96  ICD-10-CM: G89.29, G89.4, R52 |
| Musculoskeletal pain |  |
| Myalgia, fibromyalgia | ICD-9-CM: 729.1  ICD-10-CM: M79.1, M79.1x, M79.7 |
| Pain in limbs | ICD-9-CM: 729.5  ICD-10-CM: M79.6xx |
| Pain in back/spine | ICD-9-CM: 723.1, 724.1, 724.2, 724.3, 724.4, 724.5  ICD-10-CM: M54.1x, M54.2, M54.3x, M54.4x, M54.5, M54.5x, M54.6, M54.89, M54.9 |
| Pain in joints | ICD-9-CM: 719.1x |
| Depression^1^ | ICD-9-CM: 296.20, 296.21, 296.22, 296.23, 296.24, 296.25, 296.26, 296.30, 296.31, 296.32, 296.33, 296.34, 296.35, 296.36, 296.51, 296.52, 296.53, 296.54, 296.55, 296.56, 296.60, 296.61, 296.62, 296.63, 296.64, 296.65, 296.66, 296.89, 298.0, 300.4, 309.1, 311  ICD-10-CM: F31.30, F31.31, F31.32, F31.4, F31.5, F31.60, F31.61, F31.62, F31.63, F31.64, F31.75, F31.76, F31.77, F31.78, F31.81, F32.0, F32.1, F32.2, F32.3, F32.4, F32.5, F32.9, F33.0, F33.1, F33.2, F33.3, F33.40, F33.41, F33.42, F33.8, F33.9, F34.1, F43.21, F43.23 |
| Menopausal symptoms | ICD-9-CM: 627.x  ICD-10-CM: N95.x |
| Taxanes | HCPCS^2^: C9127, C9431, J9264, J9265, J9267, J9259, J9170, J9171, C9276, J9043  NDC: 00024582411, 45963061359, 25021021305, 00074433501, 00143920501, 00172375377, 67457078108, 00703476701, 00143920401, 47335028641, 45963061356, 63323076350, 25021024501, 00015347530, 44567050501, 63323076306, 47335032340, 00409423501, 25021024504, 00409173201, 66758005001, 70860020017, 25021021350, 00015347627, 63323076352, 66758004303, 45963079056, 00703476801, 00703476881, 45963076552, 55150038001, 00955102001, 16729022850, 00555198414, 66758095004, 42367012125, 00069914411, 67457044917, 00069007801, 25021022201, 00409020120, 67457053208, 55390011420, 51079096101, 00075800404, 00409036701, 51991093898, 00703476681, 63323076305, 43066001001, 50742046316, 00703321301, 72485021401, 10518010207, 51079096201, 45963061389, 43066000601, 70700017522, 00069914122, 43598038957, 63739097117, 00075800301, 55390031420, 39822220001, 47335028541, 00409020127, 10518010209, 10518010208, 66758005003, 51991093798, 00069914111, 67457053102, 47781059507, 66758004301, 45963073454, 00069914222, 42367012129, 63739093211, 00703321681, 25021021317, 00172375494, 16714013701, 55390051405, 00409020110, 55390051420, 44567050601, 51991093698, 00955102104, 16729023165, 00703476401 |
| Tamoxifen | HCPCS^2^: S0187  NDC: 63739026910, 00310060490, 60429091030, 57866661801, 00555090414, 60429090960, 00172565780, 51862044610, 00591223330, 63739026942, 00555044663, 51862044760, 00093078406, 00378027401, 51862044710, 51862064310, 00093078486, 00310060018, 63304060130, 00310060430, 51862044605, 63304060060, 51407043960, 00054483413, 51862068201, 89141012301, 00093078405, 51862044918, 00172565649, 68382082701, 68084092411, 68084093511, 00310060025, 00591223218, 00591247330, 00555044603, 54868300402, 51862044705, 00172565760, 00378014405, 63304060190, 51862044718, 00054883125, 00093078210, 51862064260, 00172565658, 00310060060 |
| Aromatase inhibitors | HCPCS^2^: S0170, S0156  NDC: 59651018030, 68382038306, 16729003415, 54569573200, 35356027030, 54868500000, 69117000301, 67877017110, 68084080321, 60429028630, 60505325503, 16571042103, 00378500193, 68382020906, 69097031602, 71921019033, 00378207105, 50268047615, 00054016413, 59651023630, 62559067030, 54569619800, 00603418016, 51991000533, 67877017130, 42043018003, 66435041530, 00310020137, 00781535631, 68001015508, 54569571400, 59651018090, 60505298503, 68084080311, 59762285801, 49999098630, 55111064630, 42291037490, 51991062010, 69117000401, 55111064730, 62756025013, 50268007511, 60687011211, 51079032306, 42291010530, 63323077230, 00378603405, 68258903501, 00093753656, 00054026913 |
| Opioids (non-parenteral) | NDC: 59911594401, 00247007815, 68308031210, 49999006012, 67544000230, 54868007202, 49999006030, 55045111207, 68084037311, 00603233819, 00603233832, 00591085001, 00436018201, 00591085205, 54569002402, 00406048450, 52544085101, 00472141916, 21695054704, 00406048301, 00054815524, 67544000250, 51285060105, 67544088290, 51285030302, 00555030302, 67544000275, 00364032301, 00781175201, 00054815624, 00555030305, 51079010621, 51655080252, 00591085201, 60429050030, 00093035010, 67544000215, 00045051560, 00677061205, 68094076162, 55045309404, 67253035110, 67544034570, 00591354605, 55289000598, 00228305611, 67544088245, 54569252307, 00247007830, 62037052301, 00904017560, 00603233802, 63629295203, 55887095120, 00093015001, 51285030404, 50458051570, 66813092201, 50458051360, 00603255221, 00002101102, 55289004815, 00228302110, 51285068404, 62037052305, 43063001403, 00527169801, 50991031601, 54868037801, 00603233928, 51079010601, 55045285309, 55289000524, 51991007401, 00045051160, 00603233816, 71610011146, 00121050410, 60760002230, 71610011160, 00228200350, 67544000353, 71610011130, 58016027100, 00603254921, 69238199301, 50383007906, 64720030510, 00406048462, 53265028603, 00093035005, 43063001406, 58864000415, 71610011154, 68084037301, 55289000512, 00078024305, 00045051170, 66336063260, 00677067601, 00247007804, 67544088270, 52959000304, 68030322702, 60951067570, 54868007207, 00904391660, 00591354601, 00406048410, 52544042505, 63739000401, 64720030411, 00603255321, 00904017580, 55289000560, 71610011145, 33261000202, 63629149402, 00403218684, 00677063201, 00121050416, 63304056201, 54868007205, 67544034530, 16590002360, 00086004616, 67544088240, 63304056210, 67544047460, 68462019401, 00143300001, 17856007905, 54868037802, 00054024425, 00677171401, 52959000302, 55289002620, 13107006001, 65162003350, 58864000420, 00121050404, 51285030402, 58016006200, 51079016124, 00555030402, 43353001060, 00054801304, 66267000112, 00054800204, 53489016105, 13107006005, 00603233821, 00228305811, 00187084301, 00045051373, 67544088260, 00603233804, 00603102058, 67544000390, 00364052601, 00591042501, 67544000280, 00054802224, 00054316163, 71930005452, 67544047470, 51285060002, 00172398560, 00121050415, 53265028510, 00603236221, 00228302010, 00440702612, 63874020230, 00603233820, 67544088253, 63874020201, 00677099633, 68094076159, 00603233721, 00440702690, 49999006015, 00591042505, 63874020212, 00440721130, 65234004616, 60432024516, 71930005612, 53265028550, 67544000290, 52544085110, 55289000525, 55289000530, 00555030502, 52544085205, 64376061131, 66992034010, 42195084010, 64248041910, 00525003201, 66992084010, 64376061101, 18011003201, 13517013001, 10551041910, 70362073030, 00037520030, 00037540030, 00037580030, 00037512030, 13913001001, 51772031401, 51772031101, 71500014001, 13913000901, 00406920830, 42358010032, 55253007201, 63459054128, 63459031224, 51862063628, 63459054428, 42358060032, 49884046055, 00093786765, 00406921230, 42747022132, 57881033432, 55253007130, 63459030424, 00406921630, 63459054328, 00555108201, 55253007001, 00093537165, 55253007301, 55253007530, 35356037860, 63459054628, 63459050230, 00555108501, 63459054228, 00555108401, 42747022632, 63459050630, 00093786565, 57881033132, 55253007401, 42358020032, 42747022232, 00074246224, 55253007330, 42358040032, 00555108101, 00093537265, 49884046355, 55253007230, 00093537565, 00074246124, 00406920630, 49884045955, 55253007101, 42747022432, 00093786519, 63459031624, 00093786965, 00093537065, 55253007030, 00406920230, 00406920430, 55253007430, 35356037828, 71500000230, 20482000410, 20482001215, 71500000110, 20482001615, 71500000630, 20482000101, 20482000130, 20482000230, 20482000210, 00093690245, 54868307600, 60505701302, 50458009005, 54868152301, 47781042447, 60505708100, 49884076252, 00378911916, 00591319872, 60505708102, 49884076452, 00093690045, 60505701402, 00378912498, 60505700300, 00245042205, 49884076378, 47781042747, 00591321354, 00406917576, 60505701300, 00591360354, 00781711355, 00378912798, 00591360172, 00781711255, 68115056905, 00781724355, 60505700202, 00591360372, 00093690345, 60505708400, 49884076178, 00245042189, 47781042811, 00378911998, 00781724155, 60760091105, 00781724255, 00781710955, 00406905076, 50458009205, 51862031578, 47781042847, 00591360054, 60505700402, 50458009405, 60505708502, 00406911276, 00781711455, 60505708200, 00406906276, 49884076478, 50458010205, 60505701002, 00591360272, 00591321372, 51862031478, 00591360154, 60505700302, 54868596300, 60505700800, 00406902576, 00245042305, 50458010405, 60505701102, 00591321254, 60505700600, 60505700702, 00406912576, 00378912316, 16590060505, 60505701000, 60505701400, 00591321272, 60505708602, 00245042389, 49884076278, 60505700200, 00245042105, 50458010505, 54868028700, 00378912598, 57866304901, 00378912298, 50458003305, 60505701202, 00093690319, 60505701100, 54868016201, 00245042089, 57866304301, 67767012318, 52959073725, 43353073450, 00555089804, 54569390904, 51862021701, 55045121306, 67544039753, 43353001770, 67544002363, 62584073801, 55289099730, 67544081260, 60713006915, 00591217501, 13551080105, 49502041601, 00591050301, 51862021705, 60760038898, 63739045510, 63874023050, 57866550803, 00904344040, 51862058605, 51862022801, 63717090101, 31722094205, 00603388704, 65162011850, 00603389004, 42858013901, 00406036305, 67544002446, 54569030308, 53746011005, 00591261105, 55887095240, 55045198006, 00406036201, 47781039760, 58016019500, 59630091110, 67544002367, 52959038000, 27808011601, 00121231650, 43353050345, 00785112001, 54868423705, 71930002743, 68115017140, 43353073467, 52959032420, 68084085295, 00603388528, 55289073760, 43353032460, 00603388328, 58016022940, 49999058801, 53265032850, 55045121307, 47781039560, 67544075053, 13811065710, 00254360035, 52959031220, 00179011240, 53746011801, 00074194914, 68115017060, 00406036010, 00603389028, 00406036091, 00682080801, 71930001952, 55045121305, 00406036323, 52544016105, 00406012323, 00591050305, 00677118401, 54868358500, 67544002360, 42254034020, 00093516101, 54569468501, 54569524008, 42858020350, 00247126220, 00406035862, 67544002580, 59630091210, 68308022401, 52544091301, 59441013801, 00603389002, 00591038705, 63717089516, 00603389021, 43353073415, 43353050360, 00591217605, 31722099601, 71930004412, 51079078121, 00603388212, 54868303800, 00603358621, 63304049601, 52959053324, 00603389132, 63874020350, 00603388320, 00603388921, 71930004312, 13107021201, 23490748700, 58864027130, 00074197314, 00247097816, 00591217505, 72865011001, 43353015957, 00406036001, 00904682661, 59630091310, 67544075070, 42291033201, 10702019301, 49999027700, 67544075072, 54868514603, 52544038505, 63304049701, 00603388822, 52544050301, 67544002453, 00047044824, 27808003702, 67544002560, 55887044301, 54868516704, 54569434700, 68115017100, 65162067550, 71930004212, 67544002472, 65224091560, 68387022030, 00406012523, 00440761090, 43063035624, 00603389022, 55045292101, 35356058630, 67336091201, 67544002460, 00406036501, 00254203501, 65162067510, 66336067015, 43376031060, 00074304153, 67544026930, 52544050205, 68387023060, 00406035823, 43063035915, 58016006390, 54569332209, 27808003601, 67544075060, 00785635063, 16590011372, 00254359628, 65162069650, 00591316801, 00879057405, 00603389121, 49999016990, 00406012512, 47781041360, 00247007915, 00406035710, 00591260905, 54868372905, 58016023250, 10702018950, 67544002545, 00591217101, 63739032610, 68387023630, 50268040815, 43386035101, 00247103608, 16590011972, 67544027980, 51079077920, 16590011530, 55045306504, 65224035060, 54868474700, 00406012412, 00179011630, 54868474701, 64376064805, 55289096540, 55700026106, 00406036791, 23490748709, 62022066301, 52959031200, 64376064901, 23490708500, 59011027660, 52544038705, 67544002420, 68387023690, 51862021505, 68084010001, 65162011050, 54569273602, 00182003405, 68084060111, 49999005305, 55289036020, 53746011210, 62584073833, 58177090907, 67544026990, 51079077921, 54569596400, 43353042260, 00406012410, 43376035060, 21695026820, 23490784900, 51862022705, 58016075835, 60429059105, 53265032950, 67544067075, 64376064301, 55289036060, 58016049560, 60429050960, 55289073706, 52959073802, 49999005390, 00527164905, 51862021501, 43353073454, 42858020101, 43353015967, 00254360038, 65162069610, 00121065516, 47781039660, 00603388804, 00121477215, 00603388802, 00121477205, 27808003503, 00406012301, 53746011901, 55289099706, 00555089502, 00406012562, 66116013030, 54868228106, 46672005250, 13107021105, 00591260701, 00254360028, 43063000403, 55887029512, 58016066290, 10702019401, 54868303807, 55289013760, 67544002428, 00406036191, 16590011920, 63739013001, 60429057201, 60429050918, 67544026953, 67544002320, 43386035801, 68084036201, 50474090250, 00440761060, 00603388821, 60429057390, 00904344061, 65224032060, 00591217201, 10702019110, 00121231615, 60429051024, 43386035301, 43376023010, 43376032060, 67544002357, 00591085301, 67544002475, 00603388828, 47781039260, 68084086309, 00247007998, 68308022301, 66116062320, 00406035723, 67544002350, 66267011040, 68084022701, 00254359635, 76181000125, 60429059201, 00603388728, 51079077721, 00603388516, 65162011510, 00074227712, 51079078099, 66689000450, 55289013730, 00603388120, 00121477210, 00591261201, 00406036762, 54868497607, 00364074405, 43063000402, 00047044830, 63739045501, 43063068690, 00406036523, 54868372902, 43353042253, 00904642061, 55289026810, 00456060101, 00406012423, 58016075812, 63629542201, 00074194912, 21695038660, 71930004352, 11528042001, 16590011910, 55289026890, 52544085301, 10544031615, 68084088411, 52959052120, 60429057290, 69101060008, 68084088409, 67544039770, 13107002105, 43353015946, 00603389032, 63629542801, 67544002590, 59011027360, 52959031206, 55045253907, 67544081253, 27808011602, 43353015972, 43353015980, 51079093321, 67544002445, 51862021601, 31722094305, 00603388820, 00603389728, 49502041505, 17478045016, 43376022010, 53265033310, 50474091001, 43353050330, 67544002457, 00406037801, 67544027930, 00406035833, 60760034960, 47781039460, 00247007900, 00406035910, 43353018753, 00555089602, 00785635001, 00121065504, 55887062315, 43353015954, 00904641961, 58016079300, 00023602201, 55289099704, 54868409900, 55289026820, 58016075889, 43353032490, 68084022711, 13107002005, 49999016960, 55887062314, 51862058601, 65162014510, 00406035891, 00591322801, 00121477107, 52555007605, 00781160601, 00904763060, 62022081801, 00555091504, 63629153208, 71930001912, 60429059101, 00603388520, 60687041801, 00781153205, 59011027160, 58016023200, 00603388228, 60429057330, 55887068082, 52959053340, 00677162201, 43063009102, 00591260505, 67544067055, 67544002394, 67544023970, 66336040894, 31722099701, 00904763240, 67544002490, 54868516707, 00603388732, 53746011001, 00591261005, 50991057801, 00591050205, 63629542204, 67544067030, 67544067053, 23710090101, 00179011290, 50474093001, 67544067045, 52544016201, 63304056005, 67544026970, 00406012510, 66267010960, 00254360135, 53746011910, 52544054001, 00406012330, 51079025401, 42858020201, 53265033110, 00247007902, 00121465510, 00121477240, 61808012001, 54868505902, 54868303801, 00247034200, 55289080260, 16590011772, 54092013801, 53746011810, 52959038060, 00603388220, 51862022901, 43353073490, 76014000125, 54569030304, 67544067090, 00603388221, 00406035901, 00785112201, 60429050990, 27808003703, 55289034815, 43353073455, 67544067080, 62037052405, 67544026967, 53746011401, 55887029015, 53265033550, 65224033060, 67544002553, 21695027210, 52959052130, 58016095002, 60429057212, 57664011188, 60429051090, 65162011511, 23710090201, 65224093060, 63739014101, 00131210437, 60429051018, 21695027308, 68084089511, 54569552300, 67544002653, 71930002112, 72865011101, 66689000550, 61808012005, 11528041001, 68084089509, 54569831400, 00339404912, 63481069870, 00603388116, 58016043603, 00247007904, 54868409800, 68387023012, 00406012462, 13107002001, 53746011905, 54868303803, 52544053905, 13107000401, 67544002570, 55289036024, 60793002401, 60429057312, 00074305453, 00904763261, 60951063970, 66869012810, 43063068506, 43386035601, 66336044220, 66336044215, 66689000660, 00591054005, 00254359238, 60429050901, 51862022905, 60429057360, 67544002310, 67544067072, 54569524000, 54868303101, 60429051060, 67336091105, 00406036105, 68084035301, 59702067416, 54868505900, 60951064170, 55289065130, 00403227715, 57664011113, 00023602101, 33358016840, 54868007101, 50474050001, 63874020342, 50268040015, 68115017330, 00364074401, 68084014411, 54569524005, 43353042275, 00603389122, 21695077430, 00349849453, 54868184501, 18837006490, 53746011305, 43386035205, 54868423704, 58016019512, 51079093320, 54868514604, 58407009101, 43353073420, 42806011201, 72865011201, 63629294704, 00591038701, 00406035791, 42858020301, 55887068016, 00406012405, 67544002330, 53746014501, 16590011430, 67544002455, 00536391401, 67544002575, 52959031215, 00904344260, 00440761091, 67544039760, 51079027421, 00406036605, 54868303802, 43353032470, 60429059001, 66267010860, 00406035801, 54868497605, 00247103620, 43353073410, 49999032760, 27808003502, 58016066200, 63304049705, 65224034060, 50268040011, 00406435701, 50474090701, 00603388712, 54569030306, 27808003603, 53265033350, 60846090201, 00047048630, 00254203601, 62793071001, 00904763160, 64950034047, 42549051490, 42254010020, 66336040860, 64376064305, 00406036362, 51079077701, 00172564360, 49999001990, 67544002450, 65162011010, 00406012312, 57866018001, 43353015950, 00121154440, 27808003701, 54868403401, 65162012050, 50268040115, 00406036662, 63717090201, 49999001700, 55045198002, 52544072901, 00603388826, 51862021605, 00603388322, 00074227714, 00591320205, 00603388122, 67544002354, 66336067006, 67544027990, 00603388721, 31722094201, 00603388321, 00406037805, 51079086721, 00406012305, 10544035702, 00406012401, 00254359528, 58016063290, 49999001790, 00603389016, 43353050353, 43063033960, 00406036705, 53265033511, 43353072160, 10702019010, 00603388332, 00781153201, 50474090201, 63739013010, 35356079601, 00247007906, 50474093201, 55289089415, 49999001760, 67544027970, 63481066870, 53265033011, 47781041160, 23490569404, 00074227454, 63739013115, 63874023020, 63739013110, 55289099720, 67544067070, 57664017088, 00047048624, 54868007105, 43353050375, 43353018740, 55289036006, 67544002670, 68084088401, 27808003602, 53746011410, 13107021101, 68084086311, 35356079620, 52959031212, 00591054001, 00406035762, 00406036301, 67544025853, 50268040315, 63874029371, 67544002377, 67544066960, 55887095213, 00044072841, 67544002361, 55289011612, 13107021301, 68453091210, 62037056701, 00406036701, 65224094060, 43353015953, 00254203401, 00044072702, 54868098200, 00591038801, 00591261001, 63629317802, 00406536105, 00785112250, 55289094415, 67544067046, 00023602205, 67544002315, 67544002630, 00904682561, 43353073453, 27808011402, 50474091050, 53265033050, 51862058701, 52959018520, 00254359438, 00603129558, 66336044224, 65162011150, 58016084590, 67544002375, 53746010910, 00440760510, 00254359828, 00247061300, 52959041512, 00406035709, 54569602702, 00406036391, 53265032810, 00406324301, 23635041601, 42858030416, 42858041616, 59011045405, 42858033801, 00074241512, 54868496901, 58177062104, 00054439225, 54868496904, 35356051430, 00406324401, 59011031560, 10544037404, 00527135501, 42858023401, 00591362901, 00406331201, 35356081630, 00406333201, 58177029904, 16590094230, 00591363101, 00054839224, 00574029401, 68084042301, 42858030125, 63739027510, 42858030225, 13107010901, 00406331601, 13107010701, 00527135301, 51224010250, 00054026525, 43063005106, 54868496900, 42858030101, 00254361128, 00254361228, 58177029811, 00074242614, 00406324901, 00074245202, 00074241612, 58177029911, 59011045210, 00074241654, 42858030250, 58177062011, 31722012101, 60687057911, 00054338750, 00044102203, 13811070210, 00044102802, 00074241514, 13107010801, 16590082728, 58177062111, 57664076288, 00187325110, 00527323737, 00527323637, 42358010310, 42358010210, 66336041699, 57664046708, 00555038102, 00591072701, 51224015550, 30698033701, 00603442421, 00406711301, 00024033704, 00555039202, 00024033505, 52152015804, 57664047108, 00024033206, 00054459625, 42806005030, 00024033504, 00024033502, 42806005101, 00054459525, 00024033506, 00115702701, 00054859611, 54868515103, 00054354563, 00603441521, 52152015802, 00024033705, 00054855411, 66689069439, 54868285403, 54868494806, 23490587809, 00054355663, 42806031801, 68462080101, 66336017060, 00406052710, 00406575501, 60687021411, 68115057100, 00406697434, 68094003162, 66689069479, 00054039168, 00406622505, 00406577123, 00054421825, 68462080001, 43063022260, 00054457025, 68084073811, 66689083699, 00054421925, 51079089840, 00904653061, 72865012001, 00054855424, 51079069439, 67877011601, 00406575523, 00054854725, 66689089840, 68115057200, 00054355344, 00054070925, 63739000610, 54868494807, 00054355367, 42806031701, 66336017160, 00054454725, 00406577162, 57866310604, 16590066060, 66479056003, 00713019312, 00054378663, 00527190662, 68084015801, 49999084660, 68084040301, 00054375150, 68084040501, 57866310602, 42858080101, 46987041011, 00034051910, 68084015901, 62559017001, 62559017105, 68084015711, 46987032411, 54868403300, 49999084960, 00034051490, 68094000159, 52544001160, 60793043101, 63857032306, 00034051610, 00054479025, 63857032411, 60793021101, 63304045301, 00713019412, 00023601960, 00574711412, 46987032311, 00406831505, 00406833062, 00832022700, 00228350606, 00228309211, 00228350311, 49884083801, 60793060301, 60793043520, 00228434711, 57866705104, 64365050702, 00228350706, 00591345101, 00245016212, 46987032711, 50383096530, 00054479225, 00034051612, 00406800315, 00245016312, 00054023763, 35356004730, 00591374301, 54868403301, 46987041211, 58177088601, 66591085141, 67877067101, 63857032206, 00054023524, 00054023624, 68382090401, 63857032311, 27808008201, 16590066056, 66479054025, 66689003201, 46987032211, 00115127701, 63857037711, 58177088880, 00054040450, 68462020301, 00054051741, 00406838001, 68462020201, 68094075401, 49884066801, 68382090701, 00054480527, 49999084860, 62559017005, 27808008202, 68462020205, 00378265801, 60951065270, 51862018601, 00054023625, 00832022800, 35356033400, 63857041211, 60951065570, 00034102510, 23490791309, 54868319102, 59011026210, 66479056024, 00527142536, 54868497305, 00832023300, 42858063101, 00406800312, 60793060401, 52544003960, 60793043320, 60793021201, 00378266101, 52544089660, 43063027260, 68084016001, 00527142562, 46987033011, 00832027311, 66591054110, 66479054225, 69344011111, 00002255002, 51862018701, 00034102630, 00591345201, 57866705102, 54868596402, 60951065285, 54868403303, 00832022750, 63739089910, 00832022650, 35356005130, 54868319103, 00406833023, 00054880524, 60793043301, 52959018290, 54868319106, 00527142563, 00574711212, 60793043001, 00378265901, 49884083301, 00228309011, 35356005000, 63629108801, 00054035244, 00832022900, 00023601560, 46987037711, 59011026010, 16590060730, 63857032611, 58177031304, 00245016112, 54868403304, 42858080401, 00228350206, 58177088657, 00228350511, 00904655761, 00034052202, 68084015701, 00172216560, 54868497300, 58521011050, 00115128201, 00054023741, 60951065870, 66591087141, 00115128101, 63304045101, 66689003216, 00228350406, 49884083601, 00034102610, 35356033500, 00904656061, 68094075458, 42858079901, 00591344901, 68382090301, 60432012200, 00310034550, 00023601460, 00054879311, 00034052102, 00034051412, 54868457300, 00121090494, 60793043501, 42858090001, 00054858224, 35356005030, 54868211501, 66479054010, 63857041011, 00904655861, 00034051712, 16590047330, 68382090501, 54868550801, 00023601260, 46987032811, 58177033004, 66689003316, 35356004900, 58177088656, 51862018501, 00832027411, 35356005200, 58177034004, 00527190635, 00054378563, 66479054125, 00054023525, 43386054001, 00527190663, 00406838023, 54868457200, 00054023849, 00228309111, 00406083012, 00378266201, 52959040630, 54868494401, 00054051744, 63857032211, 00054858324, 60999012010, 00034051725, 10544037228, 00228350111, 00121082501, 00034051810, 59011026125, 00406831562, 68084040311, 00054023749, 46987032511, 00310034510, 64365050802, 66689003250, 68084040401, 60999013202, 00054040444, 23490791203, 35356005100, 00034102515, 60793043401, 63304045001, 00527142535, 60793060601, 43386054301, 00034051690, 63857032511, 00121090405, 00034051510, 59011026205, 16590061930, 42806000601, 63481062875, 13107005501, 72245068310, 63481012785, 66336046560, 57866391307, 00054039063, 60793052501, 00406866830, 59011010325, 59011044010, 23635099101, 00904674361, 54868498000, 68084096801, 63629191101, 54868502405, 60687040677, 49999085101, 10702000801, 43063051230, 57866707602, 66336017462, 31722019101, 00603499221, 63304068401, 68382079601, 43063023130, 54868044100, 33358013040, 49999089930, 52959055630, 00603499121, 60505353901, 55887012830, 10702005601, 72245019310, 58177091401, 31722091701, 00172635460, 10702000950, 68030391301, 63481062975, 60951079770, 60429052001, 66336014510, 00115164401, 47781026501, 00054368344, 66689002450, 31722094801, 59011010010, 66591095141, 42858000110, 00115155901, 50458052679, 65162020710, 16590085590, 68071015891, 47781022905, 31722094901, 13107004605, 00781576701, 52152016511, 54868034602, 00603497928, 60951070070, 23635011501, 64950035405, 59011046010, 00904643761, 42806000801, 60687040667, 47781026401, 60951070270, 65162020610, 68308047547, 00406058201, 54868597000, 00406059301, 00603497921, 60951031070, 66689040150, 00406055223, 52544082501, 42806011601, 00054052363, 00406855605, 49884019701, 66689002416, 16590061572, 35356021160, 54868381502, 68084069901, 43063065306, 55289095110, 63629306604, 55289095190, 31722091705, 63481062270, 59630099410, 43063021910, 53746020305, 54868507603, 00456520001, 00591082501, 00115155801, 13107004601, 00172635560, 00054864816, 13107004405, 10702000850, 00054865824, 00406055401, 42858010201, 42858000401, 35356039960, 63481062375, 31722095105, 52152021511, 68084018501, 59011048020, 68084071001, 68094000559, 00603499721, 00121483905, 68308011101, 47781023001, 65162005010, 54868502401, 63481013575, 55887013391, 63481062770, 42549058156, 00406056201, 31722095101, 35356083960, 00093573301, 00603499421, 54868034603, 00591074905, 54868034600, 59011043020, 23490780106, 23635058110, 00406052301, 54868500403, 33358013020, 72865011401, 10702005701, 00406055232, 00904709561, 00555027802, 00904667840, 42291064601, 13107005701, 47781023005, 33261019620, 42549058203, 52152040802, 43598054601, 68094000561, 54868393205, 54868170002, 00228287611, 00527142635, 43598054701, 35356083990, 31722019205, 00364239501, 52152041002, 60951060275, 54868398600, 54868381501, 00054878216, 54868539000, 69344011311, 54868533802, 65162004810, 54868039504, 42549058430, 68094080101, 63629191201, 47781026301, 59011022520, 55289095112, 72245068303, 00378710801, 54868170000, 43386043101, 54868398605, 43063030499, 23490791003, 54868509700, 33358028190, 00781570301, 31722095005, 42549061090, 54868457400, 68382079301, 24090068285, 68071028660, 00406058262, 57866318204, 35356076290, 35356009030, 60429051701, 00904644561, 54868471001, 54868478301, 68382079501, 00406051223, 00879051205, 71930004612, 57664015513, 43063021930, 42549058030, 00591350101, 42858000210, 59011041520, 63629190901, 63739073910, 54868539002, 64720022510, 10702018401, 68453099210, 68071034491, 68308084201, 54868500400, 10702005750, 00904643961, 59011010320, 65162005150, 10702080101, 60951070570, 00054039344, 16590061630, 00364060501, 54868539005, 68308040547, 54868590202, 00603498221, 57866391309, 13107004501, 16590067710, 54868500404, 68308010801, 59011010720, 24510011010, 55887014150, 00527166905, 35356000930, 52544074901, 00536567101, 42858000101, 43598054605, 59011048010, 58177067704, 00591350201, 10544038230, 68084037901, 68382079401, 59011010925, 60505353801, 42549061890, 54868500406, 57664037088, 55887013182, 10544062110, 57664022388, 00904682892, 13107005601, 31722019201, 00115702601, 54868460400, 59011010510, 52544073701, 60793052601, 59011010310, 63629377401, 54868539004, 52152021411, 68084018511, 43386049101, 72887068103, 00406052205, 54868381401, 66813085001, 67204096001, 47781026305, 66813082501, 00555065802, 64011016804, 49999085215, 54868277105, 68308084101, 54868381300, 68462034857, 10544038156, 68084069911, 00406852001, 69344021311, 43386092060, 47781019605, 00093003301, 63739036510, 00603497821, 35356083930, 47781026405, 54868502400, 00406052201, 23490780101, 43063002506, 00182146501, 58177068304, 00591355101, 71930002330, 31722091805, 10702001801, 00904709461, 00406059401, 68084035411, 00591093201, 59011044020, 66591094141, 49999085790, 57664022488, 60951061070, 00904709361, 68084082821, 16590065390, 10702018601, 43386049201, 57866416505, 54868044103, 24090068288, 00182146505, 63629377501, 68084035511, 66689002530, 54868381400, 72887068203, 59630099210, 00590012775, 66479058403, 43063024630, 00590012785, 23490759203, 68084037801, 63629191001, 43386043401, 43598054705, 72865012801, 59011010025, 63629306603, 57866007702, 42806000701, 63481062275, 54868533804, 66336017494, 00406051232, 43386043201, 33358013130, 23490779901, 31722019401, 54868502403, 57664015588, 31722095001, 63481012775, 68308084501, 31722091801, 00378710401, 00406053205, 13107004401, 59011046020, 63629377403, 16590092530, 00904644461, 00406853062, 54868502408, 00555077802, 00054465725, 54868381301, 43063023160, 00182917501, 60951079670, 16590067172, 54868498003, 16590061882, 00591093301, 00603499021, 00054368663, 16590061772, 54868381403, 60951070170, 00054055129, 00228287911, 00406053201, 54868051000, 64950035450, 63481062185, 59702068301, 24090068188, 00406851062, 00904659061, 55887014120, 58177046104, 65162004750, 68084037811, 54868507600, 54868051001, 50268064411, 24090068385, 52152004104, 66591093141, 50268064511, 00555065102, 58177044504, 10702002306, 64950090110, 54868170008, 00591270801, 54868039503, 54868500407, 42858010250, 00904682894, 54868381404, 00115155701, 63304068301, 65162004950, 63739063610, 66479058350, 59011041510, 52544082001, 00054866524, 60505353701, 72245019303, 63481012770, 35356044430, 00603499821, 60951060285, 00406851501, 00406051201, 63481062970, 00054465325, 58177046204, 50268064611, 47781023063, 47781022901, 57664016088, 54868498300, 57664018788, 54868170004, 54868381504, 49884013701, 68774016301, 52152007502, 00378611301, 00603499228, 60951079470, 00406100901, 63481081801, 64896069613, 63481081301, 00054028325, 00115123201, 64896069801, 00115123113, 63481081320, 64896069501, 00115131513, 35356038030, 35356038860, 00115131701, 00115123413, 00228326206, 63481081460, 31722092901, 63481043970, 63481043870, 10702007106, 63481081860, 63481043470, 00115123313, 00115131601, 63481069370, 00228322806, 63481090770, 64896069701, 63481061775, 63481067470, 64896069713, 64720025810, 35356038060, 63481081660, 00228326211, 64896070001, 35356096730, 63481081820, 35356021401, 16590060930, 63481055370, 64896070013, 64896069913, 63481081601, 63481081360, 35356049901, 63481081720, 60951079570, 00054028425, 00228322711, 00115131713, 63481061370, 00115131501, 63481081260, 64720025910, 35356049960, 00228322911, 00228326111, 00228322906, 10702007001, 00115123401, 16590074756, 23490607301, 00024192704, 52544039501, 60429057001, 52544039601, 00024195104, 00591039501, 55045309901, 63304050601, 55045303708, 52152021302, 00406311801, 61621043011, 61621043001, 69865026501, 50458086101, 50458083002, 50458082004, 35356081060, 69865026002, 24510017460, 24510005860, 50458084004, 50458083004, 69865022002, 24510007510, 69865023002, 53489049901, 63629286801, 00045065910, 00172651500, 72888008005, 43353007292, 67544027570, 60760065730, 53746061710, 00172635960, 68115091700, 00904555660, 71610039240, 54569596700, 43353007245, 68180038406, 57664037708, 71610039860, 71610000648, 67544058270, 43353007230, 16590022920, 47335053383, 65862092201, 54738021002, 71610039254, 65162061711, 68382031910, 68387090030, 71610000620, 49999012901, 50458065010, 65243038009, 68382033401, 65243038324, 54569408901, 71610039280, 35356005690, 10135051905, 00172635910, 16714011111, 43353007228, 16714011105, 67544020770, 49999089610, 00378808805, 68094001761, 55045326202, 76439013650, 47335085983, 66336091594, 68084013901, 50458065530, 55887065860, 43353007290, 55887065830, 58864067820, 43353028330, 49999012960, 42549052730, 76218070805, 10147090103, 51079099156, 69420515001, 49884094605, 54868463806, 71610039285, 68084049611, 67544027580, 00406717110, 00904555661, 57664037713, 52959081430, 57664053788, 71610039220, 67544020760, 52817019500, 43353007265, 71610000615, 63739067110, 65862092205, 43353007280, 16714011104, 17236036305, 16590022940, 51079099101, 47335086183, 00045065970, 35356054730, 12280005200, 16714048102, 43353007250, 16590022971, 55045335003, 52959068812, 55289071994, 54868463800, 59630082503, 71610039846, 71610039272, 54569543600, 00062065530, 18837025930, 65243032406, 62584055911, 49999012920, 71610039265, 71610000698, 63629286800, 67544027592, 72888008000, 60429058805, 53746061701, 54868463805, 55887065845, 55289071990, 60505264405, 57866878706, 65162012711, 42549032730, 65243030009, 58016002800, 00904555640, 63874053263, 54569596705, 71610039245, 67544020780, 71610039885, 55289071940, 52959081418, 21695056330, 43353007289, 71511030116, 65162012710, 52959068803, 35356005530, 71610000685, 67544027553, 54868558400, 55289089530, 16714011106, 59011033430, 68180069906, 69543013610, 71610000675, 68025005330, 66267021012, 49999069330, 00440857030, 13811069130, 65162012750, 00172635970, 71610039845, 71610000650, 49999012900, 55154254104, 71610039890, 58016061700, 67877032205, 71335167603, 00406717162, 54868360502, 43353007298, 00185031101, 21695013245, 59011033530, 60505264401, 76218070801, 00378415493, 10544036930, 50111061602, 43353028380, 49999012930, 60429058801, 43353007220, 00378415293, 71610000630, 00045065960, 57664053718, 52959041400, 52959068810, 52959041424, 71610039290, 60505017101, 00228271411, 42571011905, 43353007270, 42858090203, 50458065060, 71093011906, 67544027590, 00062065730, 43353007215, 29300035510, 59011033630, 43353007257, 58016002860, 50458065330, 60429058330, 68025007230, 00228271450, 00440857091, 71610039857, 65162062710, 67544027530, 63187006330, 60429058430, 51079099130, 71610000645, 55154254107, 16590022972, 50268077411, 00591046605, 21695091330, 67544058253, 10370022311, 67544027550, 54868529103, 66336091520, 47335053788, 49999012915, 55289089515, 16714048103, 00378808801, 66336091530, 43353007275, 54569543601, 50090080600 |
| NSAIDs | NDC: 00904546420, 00028025801, 11822020568, 00378720001, 70000036002, 00006094168, 00378716501, 66336003030, 10768712101, 60429009290, 23490542401, 63739047205, 59762738004, 10702001601, 55289059060, 00378101001, 00677142501, 49999023328, 43353021680, 52544071305, 00677117306, 52959085645, 49483061750, 43598049501, 50428306397, 50228046501, 54738057203, 68084041121, 55111036701, 52544066701, 00904606961, 54868092200, 00093014705, 46122011026, 00247009205, 18393027242, 24385064771, 00363029208, 60505010201, 00054422225, 43353039192, 54569403200, 00409228720, 49348080910, 00009348111, 46122056278, 50268059615, 60505357901, 81131070008, 43353021853, 71626030160, 68084077201, 54868455601, 68382027101, 54288012910, 10544031130, 00677151401, 69097085407, 37205026171, 49738064778, 60429009390, 62584074680, 64380080906, 67544010392, 36800036871, 00078043605, 00045046304, 52959018700, 00074379501, 50436460601, 55390048001, 00004641501, 50428716305, 59762500202, 55289067320, 00093100601, 53746046650, 72611072201, 54868617000, 49035064782, 58657068201, 00409379619, 00074009649, 49483061950, 00069102902, 50428529388, 00004620001, 13811066160, 00363074258, 63739069101, 65162046410, 21695007760, 41163052162, 53746019305, 55289029890, 60429024301, 53746013700, 00247006290, 68462040601, 70000017601, 00074081702, 12333098751, 55289014090, 21140074152, 00603021621, 00781505631, 00363125224, 33358019930, 10768701902, 63874032304, 68001028100, 53489044205, 51079028119, 57866460401, 10135014301, 69238110205, 69367030201, 53329067730, 59779019780, 42291053250, 49035012694, 71610038792, 15127033501, 60429029901, 73684015060, 70000036001, 50428035813, 00409106801, 65862090801, 60760060714, 00029485220, 63874033401, 70030013726, 63874042630, 15370017030, 00440162890, 00904674780, 65862090901, 66267011760, 58016026200, 63323016225, 68180050201, 43353021694, 00300154530, 51655004924, 67544023330, 68382027201, 00172409970, 69097015807, 42494040001, 43353075280, 54868397300, 43063039630, 00904621640, 42571014405, 63739013715, 65504000695, 58016020800, 63868075940, 63868046550, 75834023660, 55111010101, 30142016634, 49999007100, 55289005207, 43353039392, 65862052199, 58177030204, 00904585361, 00121183605, 50580049824, 57894007102, 43353027380, 54436002002, 65862051501, 42291062860, 11845107208, 00054422221, 50732074701, 66424039610, 53746046605, 00247005845, 60687049101, 00074379906, 42211020429, 60429046105, 00045060304, 60429030001, 64679075702, 58016072430, 00332319309, 59137051504, 16571020106, 68462032590, 77771015660, 33358018790, 00536397901, 30142064778, 54868113300, 60505384706, 62250069102, 00228261450, 63874060701, 68180018506, 69238110105, 55370014108, 87701040485, 42254008915, 59911360701, 52427027430, 59779064771, 71610059280, 12333905407, 70748031209, 66336046330, 43063039530, 52959053930, 00781135205, 23490574100, 00904530920, 71610058330, 49884021601, 00573017130, 43063036830, 50580060107, 00363039212, 00008090202, 60809030272, 60242032501, 00009738601, 40986001061, 65162018811, 59779019716, 00363039208, 55887073630, 49348087634, 50428108886, 67544010460, 61442012710, 60346009215, 00004692006, 43292055617, 36800060471, 49884085110, 59911360802, 49884077805, 58016062800, 11822037360, 60687019425, 11673036882, 29033001301, 45802063190, 63323016212, 53265026910, 00025151501, 00113949071, 16590016420, 63981051759, 50428044588, 00247006240, 00247005800, 35356069800, 00363016626, 52959053960, 00363014082, 52959047220, 63874049501, 00472126194, 00028026401, 55289066320, 72865014901, 00143990801, 59762002804, 00378113401, 00113064785, 00591323101, 50090005407, 00904522959, 55289032812, 00363126194, 65162013250, 00074153903, 24385000926, 00603473221, 55289045120, 49999038330, 21695006700, 00536393906, 00046083181, 58016048920, 55289069330, 72266011901, 55111068460, 55111068401, 68462030201, 55289057920, 00045041660, 62011024901, 00185014105, 60429013520, 00074203602, 00904791559, 15330021810, 51641021205, 63874032390, 43063001306, 50428828595, 51655005124, 53746013150, 00003218710, 00677109002, 16590009620, 49999000406, 52735075823, 00378300001, 51079019101, 43353097960, 00591366319, 00172436160, 72789018582, 52959047160, 54458096510, 71610045692, 00573015030, 50580011060, 23155000605, 42254003420, 49999004915, 00247134328, 42291063001, 66220081511, 00548902100, 68180059702, 00008080003, 17236007705, 00172417560, 00536460505, 55700016890, 10702001610, 62584074611, 16714049802, 50428041354, 66267096400, 62011001402, 55289083710, 55289004121, 13811066050, 54569436400, 49884088905, 00025142160, 00378215001, 68462035905, 00186052039, 00006011431, 00029485120, 54868219901, 00185013910, 00258360701, 60505384801, 67253000301, 62269035029, 13668031001, 00185014005, 70954015110, 00093101593, 43353059330, 55111068205, 63739013701, 55111048701, 00172364870, 49348037469, 49483060401, 00093031401, 00615356363, 00781178901, 24385036134, 52959045640, 21695006690, 52959008030, 63874032330, 57896054001, 51079019001, 62011003002, 50580018404, 00247006298, 00378014701, 66267041820, 67544045653, 60429024401, 50580011009, 16714073401, 00047092224, 52735076401, 50428002808, 00536393902, 68084096901, 54868043903, 67544052520, 45802005705, 57866026205, 68387021030, 60242032701, 00904553661, 70000019702, 00536109306, 76282015210, 75834023805, 51079059619, 70000024001, 11822317350, 63629146909, 00172407960, 00074230630, 00781118701, 00781178910, 66993016130, 52959051614, 00069323066, 45802049071, 70030014676, 37205064605, 00440762506, 66860008503, 11822361590, 55289014215, 62011001001, 00904523051, 60429009427, 67544010480, 55111048705, 10544043102, 50580048111, 55289014220, 00247005360, 00009017007, 00536358702, 17236056510, 68180039701, 11673005705, 33358018404, 49614011685, 68115043760, 11822074040, 00093053605, 49348063904, 00045044816, 68382005001, 69367030105, 53489062001, 59779089734, 00603402328, 00904117852, 68382027314, 00364076605, 55111068430, 55887080860, 59137053000, 54569521202, 11673036871, 00603437216, 69543038625, 25866010506, 29300025605, 15127039207, 70860070141, 43353039174, 00781128901, 54569376202, 00003281811, 38245015010, 00247006201, 40986001067, 68387027020, 00005450791, 64899002301, 00405454103, 00008069001, 51293084301, 10939019711, 11822074060, 00904791224, 16714049902, 50268016911, 70748021507, 53746046500, 68382005005, 00228263211, 58517026030, 63739018015, 54868550600, 10939067622, 41163017440, 68084070311, 19458983603, 00904774540, 65162013703, 10544036230, 54738096101, 55045125702, 10939090033, 58016032114, 71610054760, 00904791459, 55289078121, 37205084826, 55289014290, 12333905401, 45802042790, 61919007530, 65162046610, 76282015201, 59133022924, 23490555702, 69336010260, 00591323001, 45802018490, 54569028500, 62011036901, 52605013905, 54868383701, 00677193401, 00440762794, 00045046311, 66336055660, 68115006915, 49035089726, 11383015121, 00093881510, 71610059753, 54569027703, 68462035805, 54868043704, 00182181000, 54738011801, 66116069312, 00074379903, 68387080030, 54738012002, 55045249000, 00074061602, 49884085101, 55513013760, 00093207501, 69677006002, 53746014005, 60809011955, 69437041501, 42582011210, 00555035201, 00904674751, 66267011640, 77771015701, 49158050710, 00074379561, 63874032515, 00025198051, 16590012530, 68001020106, 62269038324, 41163039312, 60951068970, 63739047221, 49884059601, 00228274496, 71610050192, 55953061740, 70000017603, 55953057340, 58864040030, 55289036714, 50228043405, 67544012680, 10939019701, 62584074801, 12333959401, 00045048137, 00093403001, 00247005300, 41163034020, 50580020524, 50962047560, 49999003830, 52959052060, 00591366505, 67253058044, 52959027114, 11822313500, 11822361600, 00363036878, 51079045920, 23490574408, 13811066010, 68180059801, 00527142010, 46122058041, 53746014224, 25866010502, 11822865020, 00378053101, 21695002330, 67877032030, 50844021612, 00615352713, 11845107209, 54738090201, 10768752202, 65162046505, 58016048990, 00045048109, 46122056271, 00603401821, 15330021801, 53746046530, 65862009805, 00247005399, 00005355143, 16714073202, 49999000915, 10544031930, 71610041480, 49614064778, 17236057005, 71550010090, 00904518761, 62250067630, 00247023601, 70000017505, 63874033415, 00409228831, 00009738704, 76282015301, 19458976001, 58016048900, 58517030030, 54738011913, 15127046650, 49999000914, 70882012830, 00182824000, 49348050034, 43353074953, 51079019901, 38245044350, 52605012105, 00904524660, 00045018409, 42291033860, 00378247401, 69842081178, 00033244340, 00574019360, 54569028703, 54569467200, 71610055092, 00781130601, 42571017701, 71921018201, 00409379319, 58016038890, 50228043205, 53746013205, 00904791524, 10019003004, 55289029824, 51672401601, 16590007430, 00093101693, 54868044902, 63629147005, 63629153504, 00093053601, 58016026210, 55700008830, 71610015492, 30142066026, 55953073780, 70748012906, 52735075101, 00172409870, 54868410108, 00045077004, 19458957403, 00005376123, 18393025642, 57866889101, 41163039212, 11673016626, 11845107201, 16103039308, 58016038100, 52959042321, 54569467204, 42291066201, 37205035071, 54569028901, 66993019395, 00378406605, 68084091401, 68180039902, 00363126394, 68180039601, 69367024205, 42254033201, 50111054602, 00247008000, 00378031301, 43353009560, 49035060462, 65862052201, 52959007625, 65162007810, 71610029680, 00904769860, 63304069101, 42799011201, 71610027560, 00008090103, 43353075260, 58864035990, 59630037510, 11822008650, 52959034630, 54868455802, 60760078910, 00409379649, 68001020100, 59137054504, 54569376000, 66220082522, 00025197531, 00228255150, 00363029158, 54569425500, 00009738604, 70000020102, 00364254501, 50428004814, 51293084501, 63323016214, 66267015160, 00591563701, 68084096911, 50428004212, 59779091478, 57664016508, 00573016820, 67877032050, 00093101519, 49884088911, 11822365070, 66336068615, 54868008008, 68115056404, 43353050680, 00172401870, 00247008010, 53746018805, 53265026960, 00228274610, 00591067601, 68115043730, 71921018050, 65162046603, 62584074711, 63629324802, 00677143101, 70000020101, 58016028930, 21695058810, 30142064762, 49884046701, 00378073301, 31722034201, 50111040701, 55289029830, 00074228801, 52959007790, 50428456312, 58016038210, 00228248010, 42571014560, 60429017205, 40986001074, 70954007710, 65162110305, 19458986901, 61808023005, 59746037990, 00378108905, 00440762804, 10939019601, 16571020150, 60505250203, 00009074202, 24385090526, 96295011176, 52297094685, 56062016626, 54436001704, 53746014001, 59779029115, 70677007201, 53746013100, 72789018790, 57866738201, 54257062518, 69097042207, 00904771260, 00113060471, 42291023010, 68001020003, 63187094820, 68722990801, 00093319501, 00003218831, 00409228821, 55045259202, 52735057741, 52959007602, 00591398401, 00005355943, 42291019260, 54868565001, 00378108901, 00450048101, 00364213790, 55111036705, 00247006200, 52959051209, 42291033901, 00536310502, 69097085507, 42549051890, 70000017501, 58016023620, 12333905402, 60505017600, 49348070601, 00440785204, 49738060490, 71610048980, 60429005810, 49035060490, 60505070601, 68094060061, 00536362201, 63629147605, 63629302105, 60687045711, 00536360330, 00536460501, 55289059040, 55154936605, 60429031201, 00450049824, 00258360706, 00536310506, 55289015010, 50111060801, 00005328443, 00247005730, 21695058825, 42582011310, 11673066026, 00517080125, 75987003104, 59651034930, 00086009010, 00172409960, 77771043501, 00904791451, 00904532324, 23490597303, 51079079501, 52959027115, 55887080815, 00363016634, 00045048100, 43063039590, 00338007610, 30142018383, 50228043601, 25866010504, 00005328543, 00591282005, 54868565004, 55289027230, 52959011020, 50383058416, 21695008660, 71610054780, 71610050592, 13668044201, 50436009801, 66860008403, 76218121509, 68094050359, 63629268201, 00228255106, 50428196330, 00045048148, 61808043090, 59572063255, 00182197201, 46122030971, 53746013190, 00047092230, 10135018305, 49614011678, 00113062871, 69238110305, 16590016260, 69097042112, 00536109401, 16590013860, 49738089726, 54569412302, 00378820001, 00536108802, 00364076601, 36800036862, 00904518640, 00904518740, 42291042030, 46122063262, 40986002350, 36800060485, 63868079050, 00378723305, 18393027253, 16590021130, 70000017605, 43063027030, 00009075026, 55513017707, 51079045720, 59651036005, 00172414170, 00591079301, 72888007305, 68462032560, 00093403093, 19458983604, 52959062330, 12333905403, 65862068401, 54569449402, 51407044301, 11822338650, 72865013701, 00472127094, 00573016530, 00054422131, 68382005016, 00093049201, 64899002350, 00574019530, 62011021301, 00247012402, 33358025160, 49348030610, 00363052162, 00113089734, 42571017601, 55953073770, 35356069890, 00378628110, 50228043305, 68645022090, 68180050103, 58657068001, 50428828596, 00904618640, 54569375801, 00093101610, 24385036126, 63874033460, 45802053490, 51293084405, 49999003860, 54569467201, 00904337861, 63874032450, 57866460600, 55953081540, 16714073101, 63868098324, 60429031101, 11822317000, 54569467209, 68387044020, 51991040401, 67253000305, 00573014736, 66267015360, 42291067790, 31722054305, 66267004830, 00363036882, 54868008000, 49999000430, 67253058042, 00185067510, 00025198031, 68071052930, 65162007710, 00677117305, 00045019924, 00573017510, 52959016130, 54868416500, 37205034578, 49999010050, 71610027580, 58406043501, 00364215601, 49999003890, 11822317790, 62011003003, 00363039314, 00781136301, 70677004601, 60951073870, 54868410100, 68453077703, 62250067730, 49348049934, 00641604225, 00113005705, 00573015475, 00677103101, 47781058493, 54436001204, 67877029505, 33358025875, 60429031301, 65243036409, 54868454800, 67544020430, 51079098920, 00440784090, 13668044101, 54738012102, 49348094059, 00185014501, 00536462205, 00006007468, 41163025407, 60760078960, 00185014505, 00904557720, 11822302510, 59779068526, 41163066026, 00440162830, 68084077211, 52959051620, 00904506340, 45802049078, 71862000701, 00247012100, 51672405101, 00054822325, 53746046601, 87701053760, 55289097717, 71921018001, 71610018960, 61442010360, 60505384905, 00573015498, 72789018560, 00378716005, 42291033790, 60429046101, 52959052030, 59762151703, 66267011730, 00409379501, 50111061502, 11917003512, 42291019490, 00074202302, 87701089898, 60429005701, 69512040090, 00781116410, 53746013118, 52959062360, 00009072513, 52959019324, 37205066026, 00054463825, 55289015015, 00450019801, 00904176061, 55111010105, 00591401005, 69597035030, 55289060940, 49999004290, 70860070041, 42291053101, 00904791461, 70030013917, 55953042080, 65162046650, 00121477440, 50111055601, 12333949401, 49348008709, 51407036905, 54868007403, 00045048101, 43353025730, 49884033405, 49884077901, 58016038200, 53746018801, 49035039312, 68180050203, 50228043605, 00440785120, 00093873501, 67544018281, 51079083206, 60429017105, 00045044804, 52735079001, 16714073302, 63868079150, 76282034205, 00378106605, 41163040097, 58016024115, 55289059030, 19458957402, 59651036101, 00573014718, 57896094150, 00247005328, 64814069560, 65162007711, 70030013925, 00093014710, 70000026302, 50580077010, 00113949062, 51079059601, 43353039160, 00069322066, 55289029807, 55700033030, 72789018690, 00247005220, 71610020260, 00904606940, 64679075701, 00884730809, 66267015130, 62011001403, 53746013550, 00378007601, 50428191238, 70000026401, 00247005315, 17856530905, 00364244201, 63874032360, 00004631601, 21695034072, 68115024460, 69339012406, 43292056054, 11822001820, 61145010206, 52959027121, 00904176060, 65162056910, 00045044510, 58016024345, 69150013915, 58864028790, 49999086990, 40986002351, 00904165860, 00172409860, 43353003830, 51079028120, 54569416602, 43353062930, 00172403070, 00781103301, 00364213705, 61808023001, 67877032101, 00025141134, 66267012020, 43063001304, 58406003204, 52544079205, 42799011101, 00904590347, 67544018253, 00093730606, 70030013220, 00591566101, 42291023360, 54738012101, 00028020561, 41163040096, 68462019005, 00093089201, 70000017503, 11822078948, 52544079301, 70030013430, 71610020280, 47781015530, 63868077904, 64380080907, 50268052615, 72865013790, 53746046401, 00363029108, 00045041460, 00009030503, 00074228854, 36800036882, 50580011037, 69680013500, 11788001001, 00045019208, 54569400200, 00472176098, 55289044560, 50111040603, 66479059210, 68462018805, 68084097601, 47781015475, 54569518900, 46122053460, 00054855005, 00536114730, 52605013805, 63323016201, 00450046304, 70882011530, 15127029604, 42291053201, 67544045680, 00247012101, 54569581202, 11822365680, 49035049078, 00781502401, 55045192600, 54868365903, 49999041602, 00045018404, 63874033430, 33358025360, 42291023260, 68001020006, 59762737803, 18393027362, 67253062250, 57480033801, 66267016830, 49999047330, 00093402919, 49483060450, 59651036001, 11822338490, 00045046308, 00777087602, 50383058407, 00185072001, 67544080760, 65084041020, 00904337860, 49884088805, 65557040105, 00517090225, 41163036220, 66689033901, 50383058405, 55111036601, 51079074220, 43353025753, 00004631301, 71610028360, 17236007805, 00440765130, 00904596660, 00172407760, 51079019017, 63874032514, 60429042110, 41163036878, 75834010405, 54868565100, 43353003860, 16571001201, 55289027260, 65162056524, 50580010904, 00781136305, 49158050810, 63874032404, 00781118801, 00781178760, 62107000201, 15127046624, 16590024230, 63868079101, 42211010243, 43063017404, 43353021170, 51079028298, 52605012305, 24385060462, 65862052005, 54569446800, 68180018806, 00093717006, 55289036710, 62037051501, 10939008722, 43063030806, 54569454501, 00009738504, 49999000640, 00603473421, 87701041773, 68084097832, 00054422121, 41163029778, 71610022292, 68382027214, 49035027201, 54868482601, 49999023415, 36800051778, 60429022101, 52959042390, 00378140105, 68084065801, 10768716701, 42291023290, 00472125594, 52544033905, 58016020942, 52959066330, 18837010160, 00904585560, 64899000950, 00228231750, 00005330043, 42291063910, 68001043594, 00573016989, 00904592661, 00781116405, 23155002001, 76282025805, 55953064040, 49999009718, 51674037501, 70748013006, 71626030330, 54569530301, 54738057201, 58016024100, 10702027803, 00182103901, 51079045701, 00904650261, 63874049505, 65162043603, 65162046609, 00839809506, 00536108805, 42806001805, 49614011671, 68030780106, 36800051771, 55289033215, 00228255011, 69367024105, 43353075353, 67544084053, 65162046310, 51079079401, 49999092801, 00004620201, 00781181105, 63981036879, 52959019328, 59746037960, 29300012401, 00378202001, 11822489380, 71610059153, 55289044515, 11917000555, 00904337960, 55370014107, 11673011750, 59779060490, 58864029690, 00573015020, 63629147006, 54569278900, 10544061330, 00004692409, 58864069130, 00006378464, 43353059360, 40986001068, 52959007710, 68387021060, 42291075018, 23490699102, 41163049078, 59762737901, 62269036024, 10768753401, 52959082160, 63629147007, 13668044105, 63629297407, 00591282501, 55289036706, 31722034205, 59779016634, 63739069110, 00182193401, 72865013810, 00378055505, 59088009300, 50111061403, 24385064762, 54569028502, 52544033910, 43353091870, 00378406601, 42291051701, 40986001056, 00093053701, 50228015660, 63874068730, 11673049078, 18393027262, 00093839701, 00086009175, 60429022105, 67877032001, 67877011995, 59779052162, 23490597109, 54458092305, 49035074260, 71205097730, 69097096512, 00450048103, 00182824100, 00450052415, 10702027703, 00536398101, 11822854260, 00591346601, 16590009260, 00904559060, 58809064060, 55111010301, 55289059510, 00228255111, 57866398101, 10544061315, 57866460608, 59762151802, 33358018800, 59137051500, 00172402960, 54569028707, 00409228731, 70000018101, 71205021520, 00363013280, 00093071101, 68094003758, 00074012403, 54868007408, 49348070619, 00045019204, 43353021180, 33342015611, 00182129701, 69677004002, 10019003017, 55887091730, 23490597003, 00904546330, 70030013589, 00363039308, 50268016915, 52959041630, 63629126301, 54569028903, 00527141901, 60429031401, 62939844101, 55289047530, 54868013303, 21695005021, 54738011813, 00378046701, 00033243542, 41163068526, 00172410970, 64679075805, 00045077003, 00591398560, 68462035801, 52959048330, 49884021605, 55289014021, 54868384300, 67253062203, 43353038974, 61808013001, 73684010030, 68030660902, 00904558460, 16590012690, 10135018324, 31722033901, 59137054004, 36800036878, 60505255301, 25866052812, 59779039315, 41163089726, 68387026060, 62865049071, 55289061160, 00364231601, 50580011039, 68382005105, 53746046400, 55289060960, 00440762620, 41163051771, 00591366519, 00363029214, 61919062160, 55289050130, 65162013710, 00839676306, 00781129701, 65162019010, 69344020129, 00182110616, 55289075930, 00363029112, 58657068050, 53746018950, 11673012140, 49884085010, 66267011520, 41163029129, 42291075118, 00591282505, 63874032230, 72865013890, 50580060304, 00247005806, 62332014271, 77771043605, 53489013405, 25866005440, 00781519501, 38245015050, 62011021401, 59137053504, 60951077270, 49999019430, 63187056230, 65162056950, 11383006850, 11673064785, 47682010013, 68387021090, 49483061901, 75834010301, 00004692506, 49348070609, 65162056511, 59779060482, 46122054890, 49884031901, 68387028030, 64380080707, 00364076690, 00591346401, 33358018740, 58016048930, 60429022190, 00074634702, 00781136201, 55289037630, 60429009230, 11822073311, 00247005330, 55289044512, 57896094125, 53489062105, 00472200216, 10768716702, 00603406721, 00904117580, 52959065660, 55370013907, 00006015030, 42582011218, 71610051660, 00247033405, 67877029605, 00781215360, 41163029771, 68387027060, 59137052004, 69292055001, 00591033960, 00781136205, 52959019310, 00228274311, 52544071301, 55045117307, 49884016305, 00005330243, 70010013905, 00603587321, 68084005101, 00074055402, 66658023401, 49483061801, 49348070610, 00363049071, 69150013914, 52959042320, 10544031060, 12333905406, 59085023024, 52544030301, 65862009801, 69842018383, 00025138134, 00003281411, 63874032220, 68084097611, 75834010401, 87701089897, 58016022390, 00884670010, 43063060006, 49348070614, 16590012660, 58016024300, 00677142405, 58469430330, 00904176089, 16714049901, 51991041901, 70000017606, 13811065910, 57664051213, 61919068360, 00054363063, 10768737301, 64380084307, 60951074485, 00364076590, 66336068760, 10544033812, 49884085001, 69344020229, 69238173002, 35515098528, 00172410860, 68094003701, 21695006630, 59779064790, 49348030609, 63187099460, 16590004620, 55953039240, 00904523059, 62584074701, 19458957301, 67544018260, 31722033801, 00591367005, 00113068526, 68382005101, 00573015110, 42291019560, 54868410105, 53265026711, 00074433906, 00228274511, 63739013601, 00228255096, 23155000505, 54868304305, 68405801806, 59762014001, 59137053001, 00338006910, 71205099130, 64899002305, 52735057605, 00182168205, 68084005111, 54569376205, 45802089734, 68134036301, 00378160105, 00378716001, 58864040060, 31722033905, 12333905602, 00591282001, 00904175860, 21695034320, 11822510630, 60760013500, 51655056784, 52959011130, 68115018190, 66267015220, 55289026715, 00603417828, 00781118710, 52959007700, 50428232570, 60505255401, 00364256305, 49483060201, 71610041492, 00378575001, 52959053921, 54868304303, 87701040305, 63739013401, 00364215605, 00002473230, 54868296602, 17236056801, 30142060490, 60809011772, 43353075283, 55289032815, 21695008620, 16590015960, 00005450704, 66993016030, 68180039807, 68134020116, 00113001162, 00363012318, 10768753402, 66220028411, 57480033701, 58016061900, 59779060462, 00247005340, 27854010301, 63629191301, 57896094101, 50268052511, 00641604201, 24385002734, 59911362201, 00363036861, 00472127016, 72611071925, 50228043201, 00591079205, 00008418101, 00573029001, 11917010270, 16714073201, 54868365902, 49999086930, 69336012710, 68387048190, 49884016301, 00713017630, 38245042710, 69512060090, 00364076501, 71610041460, 33342015715, 59137055004, 52605014005, 10939035633, 50580060102, 00121477405, 37205028226, 59911360801, 00591366419, 00332319109, 49999023390, 70710115803, 55887092360, 49483060110, 55289029730, 52544083801, 52959048530, 68094049461, 54569029401, 00378045101, 51079028220, 62011003901, 46122056478, 15014040090, 49348019609, 55289014040, 55806033003, 43353047660, 54569376203, 00005330131, 68094049458, 57866460603, 16590057460, 42291044290, 58016020100, 00006094268, 50428923748, 00247020260, 00363049078, 00440762810, 59651003247, 49999023490, 51655062952, 00247071830, 57866460602, 21695034160, 41163051178, 00084005214, 66267011620, 00228261711, 71610058030, 00659041830, 52297094785, 11917011658, 70859000401, 11917006896, 58016057410, 65162007850, 00045048164, 59762002802, 00228259950, 69097085412, 58406044501, 60687026801, 42211020329, 53746019005, 54868565104, 54766009306, 00172434860, 21695006730, 00781235001, 51672403601, 00440584000, 62107009001, 00364221101, 21695005060, 87701053836, 00074228749, 00247005805, 60429005718, 49348022834, 00143991601, 00409228721, 00228231710, 16590017860, 69097096607, 69097015907, 17236057001, 00603587221, 55111068305, 53746013250, 59133022844, 42291067750, 71511070116, 87701040486, 00078044605, 96295010725, 00573016540, 63323016116, 55045125706, 00005450705, 00517060125, 55370014007, 16714073301, 11822358090, 00349860901, 52959023209, 41163089734, 59762002903, 66336072790, 00006002568, 59911362001, 00093319505, 36800060478, 52959062300, 00363025510, 00074937402, 68084024601, 62037052001, 42806072101, 40986001055, 69344014443, 68001028008, 00440762730, 52544082205, 60809011755, 13811067730, 33358023301, 58016023600, 70000030802, 63981029114, 33358006930, 40986001065, 66267009560, 42543011501, 41163014082, 50428006166, 54569376005, 00121477410, 53746013705, 49035091212, 00045048152, 42571025830, 50428346253, 61145010106, 37205034571, 54868008006, 10544032030, 00409379601, 23490578906, 51672401701, 00904791580, 63323016223, 57866692402, 00603401921, 49348019635, 60687019901, 16590079430, 00904559040, 50580011038, 69036050330, 52959007718, 00045019217, 67253062111, 60429021901, 68001020108, 00054463925, 42806012601, 55953039740, 50428828594, 54868395501, 50428030774, 41163011471, 10135018301, 33342015809, 50383085075, 54868515800, 49999000900, 52959031820, 65162013210, 37205026178, 49999038360, 50228043505, 68180059907, 68180059701, 55289014030, 42254008960, 60242032750, 53746019401, 54499046601, 63323016226, 53746013724, 69097085512, 70000036003, 49483061850, 54569452000, 00603402128, 51407037105, 54738090301, 00378042701, 16590043460, 00904164860, 59762738002, 63629146906, 58016048940, 50580090924, 66336003012, 69784055001, 55111028960, 51293084305, 54569467205, 60760038220, 16571020110, 59137050504, 63629191401, 23490542404, 00247005290, 00247008002, 69842074820, 50428252797, 00074228702, 49614089726, 53746013502, 55045192409, 69238134301, 54868565101, 60951077290, 43353012953, 23490574306, 51672405104, 49614064878, 00093189301, 00182868800, 69336012410, 49348082910, 00555057249, 00904621740, 50436738202, 00591039819, 51672401801, 53489013401, 00378047105, 51079019156, 00093075701, 42211020623, 54868044901, 68180050102, 60505250303, 55045116301, 71610038780, 24385000934, 68084070301, 00472176194, 00378045105, 42806072205, 96295011210, 00009030530, 54868383703, 23490727403, 54868007405, 55289042040, 69097096507, 66267015340, 54868043800, 29300025601, 67877011901, 63739067201, 69344020623, 58864064330, 54868087800, 61442010210, 50428191234, 50428261925, 58016022300, 76282034001, 00182195901, 49614031478, 00364076505, 60429046260, 67877032130, 11822490020, 66267004660, 55045295008, 62332006230, 72789018682, 13913000812, 43353009580, 46122003860, 30142060462, 00054855006, 52959037760, 65162018810, 60951068870, 00349828201, 17236056901, 66220081522, 00597003401, 10544038720, 00028026260, 68016046220, 00093403019, 10544043030, 55289046706, 50228046605, 10939036333, 66267004820, 10939036033, 00591367101, 51645077701, 49348080810, 57664051318, 55887097690, 69329033500, 00113066026, 00364214501, 47781015301, 55953053341, 59651003212, 00045080116, 52959007730, 65162019011, 58657068250, 00093402901, 21140074151, 52959053915, 69097085207, 00045046302, 49999004206, 67877011905, 52959051660, 69336010130, 58406005501, 58406004404, 00113064771, 11822867420, 65162007610, 00004620314, 00603021721, 00054022613, 65862051601, 63739013710, 60505357801, 63739068401, 70000028801, 11098053301, 00536109406, 65162046450, 53746013801, 49348064227, 61919016330, 68030760206, 10939036233, 50428207077, 00185067501, 00363039216, 41163064778, 52959008120, 47781058468, 53746046590, 40986001064, 68645047490, 12280045610, 37205073658, 00363043815, 60687046811, 54569397400, 00113064778, 54868410102, 23155001101, 43353021670, 68180039702, 00054422125, 60809012955, 68645022254, 00378406891, 71610017680, 00009738603, 58016024200, 61442010310, 00185014601, 55045262806, 62269035024, 63874042660, 68071026130, 00378301601, 24385036871, 62011000801, 63739067210, 36800060462, 30142060471, 52959053060, 58406002104, 00573016020, 63323016200, 00025141160, 68001020008, 52152028504, 87701095927, 63739013703, 70030013725, 67877012105, 54569446801, 60429009330, 50428456341, 00028025861, 42291062820, 60505072601, 49884016201, 49035066026, 68180018906, 50268059511, 62269028624, 53746013790, 00378300005, 00409379349, 00573017511, 66267037360, 49348081910, 50428299472, 63629320201, 00378301505, 00378055501, 11383015111, 55390048110, 17236056555, 55289046710, 00113007471, 00006069361, 00093014793, 00378406591, 52959053928, 30142060485, 42195047101, 55289069314, 00045048173, 00228274396, 16590012420, 10019002232, 58177030304, 59779099505, 65162043806, 87701030771, 00677103105, 41163034014, 16714049702, 63868075818, 50580011004, 52959007604, 59762600201, 53746013200, 49614014278, 00004692606, 00536102301, 23490574304, 55289016610, 00363036862, 00247006220, 00591039760, 63874110800, 00781178960, 63874013904, 37205073657, 00904559160, 69367030205, 60429046001, 36800060493, 00093101619, 21695006740, 50268016811, 55289032830, 54569376001, 63874032228, 54868089705, 55953042040, 10544018901, 50428144778, 00591367001, 68001023106, 67544115553, 42291019350, 53746046505, 00074203949, 00088216130, 00603402228, 67544023353, 68115025290, 66336072730, 77771015705, 45802052278, 00406205005, 00364256401, 49999000630, 65162019410, 10768763101, 00677111901, 00781116513, 55289043515, 69420137501, 00440762760, 00054022725, 50580018406, 54868449004, 54569028700, 55289059620, 63629146903, 70860070142, 00378009301, 11822239990, 18837023907, 54868397401, 65504090405, 54868417101, 00781178710, 65504001292, 66267011690, 59779039214, 00781116501, 49999007407, 49884072305, 00378073305, 52427027201, 68084033301, 40986001059, 62011033401, 00517888001, 49158050610, 00074433907, 54868013305, 59779089726, 00113060485, 59137052000, 36800068526, 43063003892, 00025138151, 42571014260, 50580011013, 11822365080, 00677103201, 45802089726, 52959085615, 49348008609, 52735058126, 49348015110, 21695006620, 42291033750, 68001028000, 63874049590, 51672213008, 68115010128, 42571014305, 50580060140, 00074433902, 62559025101, 58016037200, 71610054230, 60429042101, 60760061820, 49884077905, 54162094190, 00247017005, 53746013810, 65162056810, 55513036955, 10939029522, 43353038992, 00677119706, 60429042260, 57894035001, 00440843160, 00573017910, 55289060120, 00409379561, 63739044410, 00363089726, 55111036805, 30142025510, 49035060458, 00677111905, 63739018215, 71610015460, 43353021653, 71610054430, 40986001057, 63629332801, 25866001002, 69344010101, 62332014131, 00781178701, 00677146401, 00025152034, 00115165801, 69399018481, 65162056550, 50436555901, 00093049805, 15127097804, 55289059008, 69842081162, 16571020206, 67544084080, 10135014305, 60687019911, 58864002630, 53489013301, 60951073860, 87701075606, 68180050202, 00536108297, 00364213701, 43353039360, 50580060104, 55045253004, 54868397400, 53746013505, 58016049615, 00113062878, 00904176040, 00536108901, 70720017599, 65162019050, 63874064430, 53258021801, 68084012701, 00536310501, 49999000414, 00029485121, 54868565102, 00074379661, 52959043660, 00074379902, 65243033006, 00904174840, 68115043830, 00536108806, 00143134805, 51655012826, 66658023407, 66689000950, 00591401105, 59930177201, 53746018910, 72865013710, 49999000960, 15127033524, 00182103902, 11822365010, 55887045590, 11822867410, 00093014993, 61442010260, 00781178560, 00536102306, 51079074201, 00363036871, 50228015701, 53489047801, 00182180989, 54868438500, 72611071901, 00093839901, 57866463902, 54868087502, 11822154520, 00045019202, 18393027742, 00093071105, 58016022330, 50428828597, 66336072714, 43063044560, 00247012110, 50580060150, 50228043301, 54436002204, 00009075002, 00603522321, 00378023701, 00591033801, 66336081520, 68462035901, 11917001403, 62559025001, 76282025701, 66336072260, 49348056859, 54569416400, 55887045520, 51672213001, 54569027701, 49738005705, 00364256405, 10939022644, 00044017305, 53265036310, 36800066026, 43063020540, 49348015009, 45802053478, 54868449003, 00409379661, 60760078930, 11822490010, 50428051962, 69230031112, 00009738602, 23490542309, 63739013403, 51079059624, 63874003077, 50090254802, 71610050560, 71610059253, 55370013909, 00046076199, 11822316930, 60429005801, 00003218713, 59911362101, 68180059607, 58016009790, 00378180105, 50580010020, 51672138509, 21695006772, 69344010233, 55111068469, 57866398203, 53746013224, 60505004001, 00440762890, 50228046505, 00004631401, 12333949407, 53746046690, 76282015310, 00074012404, 63874033960, 70000017103, 10019002209, 29300012510, 69367030101, 60429017101, 81131069960, 54569518800, 54569027500, 00440762610, 51079019120, 87701040493, 16571077650, 58016066690, 00004641601, 10702001606, 00121091400, 21695008690, 17236056501, 49999023430, 00440185260, 00405452901, 63739013515, 54569028902, 00069050230, 00047091424, 00028026201, 54569376204, 55289014206, 55289023960, 87701040580, 55289042010, 67544055380, 71610062992, 37205040262, 00904629760, 68016002351, 58016031330, 55289052614, 49035060482, 19458983602, 52555020510, 00378520001, 00247071803, 54569521200, 29300012410, 15127031224, 69344020523, 60951077370, 63739070110, 49884059701, 69543038810, 00008090901, 37205035078, 54569495400, 00093402993, 49483060101, 58016022328, 00904518660, 87701084242, 59779010882, 33358018815, 36800014082, 67253062210, 55289045114, 00781178913, 54868478200, 00074228711, 52959019360, 58016031300, 63629202001, 62037082675, 00143310801, 43353075380, 52544073601, 00364214550, 68180059802, 55289023945, 51079019056, 42291059790, 00143310701, 00044017301 |
| SNRIs | NDC: 27241009809, 65862052799, 68001049604, 51991031133, 51079013604, 60505377903, 58118326803, 00008121101, 55111045590, 63629334003, 00904645204, 58016022000, 13811071590, 23155024701, 66993066430, 65580030403, 31722000430, 57237001890, 68001041504, 16714003802, 00527260546, 63304019190, 68180029406, 13668001871, 13811067310, 65580030309, 68084071311, 51991031110, 47335061930, 13668002001, 75834021990, 54868425200, 59762122203, 18837004960, 67801031203, 13668011130, 68382002110, 58016000460, 60505378003, 57237001830, 00603615125, 54868425201, 54868341405, 68001041306, 42291089730, 59762018104, 68084048401, 47335038318, 16714090502, 43353026879, 31722012530, 00904624761, 58016061690, 00008083703, 00002327001, 43353079870, 00054040122, 13811066490, 63629210101, 55289003630, 55045319608, 59762348102, 60429012330, 60429086730, 00008083302, 52959089260, 00527260432, 57237017301, 68001015900, 68001041505, 52959038830, 29033004530, 12280016315, 55289086930, 00904647061, 63304019290, 00904624661, 68115039130, 68387034830, 16590048760, 57664039588, 43353069070, 55887066590, 68115047860, 47463008430, 66993066260, 55045338708, 31722012490, 13411011003, 50268028813, 00008083601, 43547038109, 68001036804, 42291089490, 63629414801, 13668001890, 21695014530, 00603615021, 13811067610, 16714004302, 00008083321, 65862052790, 64679071601, 68001049705, 43353042160, 49999024930, 66993007730, 55289002830, 55111054605, 00008070102, 60505299708, 23155024709, 13811066390, 29033004630, 70436001204, 47335038383, 00008070108, 43353010683, 16590048630, 00008121030, 52959043030, 23155024901, 00378488301, 31722000230, 55887012530, 49999059915, 00603614921, 55887027060, 65162030609, 43353042153, 67877026360, 00904636661, 16590048730, 54868293200, 68084069811, 51079048001, 00008083720, 55045336808, 65862069705, 54868341403, 57237017401, 00093738456, 68084070911, 23155024801, 41616075881, 58864062930, 44183089031, 71921017409, 33358009930, 55289003621, 59762018004, 31722000330, 68084085601, 71921017309, 00093738598, 23155025001, 67877026510, 23155024601, 33358009960, 47335075981, 00002323730, 13668001901, 51991031210, 68387034915, 50268028713, 58016061630, 54868614001, 64679071704, 44183089090, 65224088031, 68001041408, 43547037906, 16714004401, 65580030303, 54868341401, 00008122214, 00603615121, 55887019360, 00378488401, 68382003610, 13811067410, 58118326809, 65162030009, 68382003606, 60429016630, 67877072790, 67877072690, 68084068301, 65862040590, 57237001899, 75834021730, 35356049130, 55887027090, 68382001910, 00093738498, 58016073560, 58016076560, 43353069080, 68382001901, 42291025330, 58864069630, 00008122201, 68084048511, 47335075883, 33342019407, 54868425204, 59762121003, 00527260532, 00008078108, 68001049904, 41616076081, 13811066230, 54868531500, 47335080481, 00131326546, 57237001990, 31722058330, 60760032430, 16714065801, 42292002120, 68001049805, 59762121103, 68084067511, 00008083301, 00904645261, 68084048411, 71921017433, 68115050590, 59762018001, 68180029403, 54868425300, 35356058030, 13668011005, 68025008130, 57237001799, 41616075981, 31722000490, 71610040360, 44183088031, 70436001306, 00131326732, 68001041405, 54569567800, 50268081715, 41616076083, 60429016530, 51079013520, 58016035030, 00002323733, 51991074890, 13811071230, 58016035100, 50436997901, 51407045590, 60429016460, 00008083722, 68001016000, 43353042116, 16590008660, 68115097400, 21695004630, 71921017233, 00008083303, 70436001206, 43353068953, 13811071530, 43547038011, 68382002101, 54868352302, 68001025704, 65862040460, 65862040820, 71800001130, 68071044528, 55887027115, 68025008030, 75834021830, 68001049905, 64679071804, 62332001031, 00378423077, 42291089850, 57237017490, 49999024915, 00247085360, 13668011105, 68180059309, 13668001801, 54868519200, 43353023453, 67877026410, 42292001901, 33342016009, 64679071701, 00002323560, 43353086979, 65862069790, 68115050500, 21695014615, 65224088090, 57237017601, 68382003406, 68001025605, 13668001830, 70436001304, 67877026490, 00008083620, 47335079483, 47335038188, 00008070307, 43353026830, 00603615621, 60505377909, 00591366019, 58016034915, 59762018002, 65862040701, 57664039288, 51991031233, 31722012690, 42292002020, 47335074783, 68025007990, 55111054805, 58016073230, 16590084830, 68084084411, 52343013230, 16590084860, 55887066030, 42291089690, 00008070308, 43353042184, 00008121150, 52343013190, 42291089650, 23155025009, 65862052801, 43353042170, 00002323704, 65862040560, 60505299703, 16714065701, 58016035000, 16590048290, 16590048372, 55887066530, 55887027030, 51079048230, 00904645461, 51079048256, 13668002005, 52817013010, 00008070407, 16590064430, 54868519203, 55111045530, 68180059206, 43353042130, 43353069053, 00093738101, 68084089695, 00093738556, 52343013130, 16590006660, 59762018102, 68084084401, 60505377809, 55111060930, 16714004301, 00378488501, 00378513105, 27241016430, 68025008190, 16590008430, 50268079915, 51079013420, 00054040113, 57237017390, 54868519201, 68001049704, 58016061560, 51079048020, 65862040730, 23155065603, 65580030409, 72865020030, 42291089550, 21695004615, 51991031290, 00002327030, 16590008730, 49848002230, 33358012360, 00456224030, 00008070401, 68084067521, 68180029503, 43547037909, 13811071390, 00093738505, 47335075881, 13668001905, 00008083721, 68115029690, 49884037511, 21695004745, 54868521501, 21695004690, 00591365930, 50268028811, 16714004402, 43353086953, 13668001990, 00228289296, 43353079853, 16590048390, 42291089390, 00904646961, 00527260646, 68382003410, 58016074260, 65862052899, 68084070901, 66993066330, 68180059306, 71610040260, 00603615025, 55111054690, 63304019130, 00904704461, 59762348302, 31722000390, 13811066301, 60429016610, 68084090025, 55887019390, 65862052730, 55700010230, 33358009830, 51079013601, 00008121130, 65862069701, 33342016207, 68382003510, 13668001971, 13811071290, 23155065610, 68115029630, 00378513305, 33342019507, 43353088279, 00591365919, 68180029509, 68180029603, 13668001805, 57866391001, 60760032330, 55111060860, 16714065601, 54868425203, 47335061730, 54868425303, 16714003801, 59762348202, 43547038003, 51991075033, 31722012630, 00008083702, 52959089030 |
| TCAs | NDC: 42806009730, 00378354570, 49884005601, 21695025230, 45963029330, 54569596800, 16590048490, 49884021901, 00172567570, 00378268593, 00591578701, 63739035610, 00603404521, 63739018915, 68084012030, 00591563301, 60429028830, 72189010430, 59746071130, 50742011750, 57237000730, 00406992301, 62584055601, 51407035105, 42806052910, 00904126261, 43353037830, 00364057301, 69315013301, 00955103210, 13107000330, 54569017200, 51079080301, 10135069801, 60687043301, 43353096245, 52544059401, 16714044801, 51079065120, 69158000100, 00406992203, 63739009910, 65862002206, 58016081530, 52152034502, 00093720656, 45963029530, 78206016001, 67544020660, 00093096001, 51285055402, 00378262510, 00182102101, 63874109803, 59762141509, 68115041830, 13107000105, 59762141207, 67544035730, 50111043601, 68387033724, 00182119301, 70710122501, 00603221330, 00440763690, 00603404332, 57664051018, 64125013301, 70710122601, 13107000334, 00185073601, 00247117707, 00052010530, 00310004710, 68387033690, 23490539301, 00677047510, 60687029311, 00904705361, 49884005501, 42571034330, 62332031831, 50742011601, 33358011760, 00378265001, 64679055901, 53489033001, 00378351570, 65862002306, 00440709130, 51672421701, 00536307201, 57237001306, 68387036307, 53489010501, 00310004310, 71610052345, 51862001705, 60429097010, 63739035515, 00078007805, 00781197401, 00078008605, 29300042110, 60429096810, 64720041710, 00555024171, 71610011515, 58016081324, 23155058001, 43353053671, 57237001005, 00781176610, 60687026533, 53489033201, 51862001610, 66336002730, 00093081201, 51672401305, 00781897501, 50383096010, 52959053790, 42806053001, 71610036960, 71610047560, 13107000305, 00364040602, 62332063731, 52152034305, 62559071101, 00185021230, 27241016810, 43353030660, 51672400206, 64679056001, 63739019015, 00591080801, 00677047801, 68387033060, 45963034102, 00677047501, 49999006350, 00378009201, 67544008553, 62584068601, 52544069601, 49999006300, 00247061590, 55289012412, 51672401301, 51862001605, 51672400306, 53489010705, 51862094501, 67544007830, 00781176201, 65473070101, 16714044602, 35356042460, 27241021030, 60687028101, 68180031606, 00677042306, 53489010510, 00332212015, 00378312501, 00310004110, 53489033203, 00185002010, 00781280001, 00406992101, 62559071001, 00440730730, 00247124330, 16714013201, 00054467613, 65862003190, 59762141703, 62332064531, 65243024209, 50742011201, 00093052701, 68115002230, 00185073605, 42806053301, 27241017001, 52959054214, 00781204701, 00247061430, 52959035830, 00378425010, 68180031201, 00364250801, 71610052315, 63739009810, 54569017502, 00247123330, 66336067330, 00054021025, 00591578710, 68115026290, 60505024908, 42571034401, 50111043701, 00904332460, 00603221621, 10544041330, 68084003201, 54868248103, 00185072110, 53489010801, 58016482601, 00378006001, 72664021490, 51079080501, 00049536066, 50111043603, 00052010790, 00378351510, 00185022210, 51672400405, 54569246201, 70756020312, 54569151900, 59762141609, 13107003105, 29300042301, 52544059501, 67544042930, 51672400402, 68084012101, 65862000330, 00364227877, 59762141407, 00332212009, 00904705461, 30698000701, 63629236801, 68180031101, 70710122901, 00603221321, 69238117109, 16714044601, 54868222105, 16714070902, 51862001701, 00068001501, 00603345632, 00591111830, 51079043720, 51079043601, 27241017110, 51672400406, 00364251101, 59651017601, 16714085102, 51407031501, 00247061500, 00068002150, 00781148613, 00677047701, 00677042307, 65862003130, 51407035030, 70756020211, 50752025005, 16714070901, 69238106101, 00378267501, 43353053609, 00955103010, 60505024808, 00052010893, 00078031605, 00054467725, 58016051900, 00781897201, 00038004110, 51285053802, 57664050083, 50752025205, 42794000402, 52959028400, 00378268501, 51079080420, 70756020411, 67544035715, 51079064501, 68180031406, 29300041901, 58016087500, 70710122500, 54868254600, 60429097001, 65862003103, 61073003630, 60687026532, 00378307501, 51079008820, 00052010830, 60760021230, 00093032701, 00591223015, 67544025360, 00349872901, 50111036803, 60429009701, 00172569570, 00115165408, 00603221221, 00093032601, 16571068409, 58016085302, 16714013301, 60687044401, 70710122700, 51079043801, 63874043060, 62332031931, 65862003203, 68084012130, 51672400106, 53489033003, 70710106703, 00378641001, 71610052360, 51407035230, 51672400105, 70756020611, 55289001801, 00536307501, 00093961212, 00440782530, 00054867725, 49999090900, 64720041910, 67544016915, 62332031531, 69238117009, 00378353001, 58016081500, 27241016701, 68180049201, 00247061450, 65862003290, 69315013501, 62037075410, 00228213210, 10544032930, 71921016301, 00052436501, 00536307301, 67544008530, 57664069288, 10135069901, 00052010606, 00677042106, 54838051240, 52817028210, 16714084901, 64720041510, 76420071006, 00406992501, 17236000601, 43598056930, 54738091201, 43353033116, 00364252501, 00332212415, 51407035010, 58016085390, 62037075310, 68084003111, 51079043620, 00406880701, 42794000702, 00052011090, 00049534066, 00781263001, 58016051960, 43353016353, 52152022704, 51079010763, 00093730318, 71610047515, 00603221202, 71610010053, 51079056301, 57664051083, 00832063211, 70756020112, 50111036701, 00185002901, 69158000000, 52959000860, 68180049301, 55887039860, 00378104910, 65473071901, 00172567600, 43598056730, 59762141007, 62584075001, 42571034230, 00364211402, 51991094601, 00677047601, 67544020630, 16714085003, 00052011006, 33358002300, 51407035130, 00591247015, 00054867625, 51079013163, 00093081305, 54738043703, 00781148901, 00781184501, 16714044802, 51672400201, 71921016201, 21695009490, 00603221421, 00603345721, 00093095801, 00677042201, 00536307401, 00378417501, 30698001101, 51672401206, 00378354505, 17236035710, 00781197301, 00378305001, 16590017130, 67544006330, 51079056320, 63874043050, 00028002026, 67544007860, 51672400305, 00591571401, 71610052330, 66993071230, 00182101989, 00781263301, 51672401106, 00781197501, 55887055230, 00781197650, 00364211501, 68387033560, 00332212409, 00172567546, 00378265010, 00185001910, 52152025418, 69238117001, 16714026201, 50742011501, 69584042510, 00008415801, 00378354501, 00378267593, 60687043311, 23490504704, 16714085103, 51672401201, 53489010401, 69238117309, 16571068509, 00093720793, 60429028790, 00247077330, 00781897401, 43353053645, 00054021125, 52959000890, 55289083930, 69238110809, 13107003205, 62332040931, 33358019130, 00247120130, 00591111730, 49884065501, 60429096910, 00406993303, 54868006402, 43353016360, 00591562901, 57664068888, 43353096253, 67544016953, 00052010630, 00378351593, 27241017101, 00093720619, 50268060511, 51079013101, 69315013510, 64125013501, 00028002201, 55045168200, 55289001630, 00677042301, 59762141503, 00406992303, 59762141505, 16571068309, 52152034202, 00310004210, 62332031631, 16571068303, 71921016501, 42806009630, 50111052401, 63874031101, 00054027413, 58016051912, 51672422101, 00406990701, 00781149001, 00591037901, 16571068503, 00378354593, 00904020280, 00839619216, 29300042401, 67544025330, 00904020161, 51285059402, 52152025618, 55700024190, 62269033124, 27241021201, 57664049918, 21695025130, 69315016601, 00591563001, 00440747830, 70756020212, 43353053653, 53217014401, 50111036703, 00052010930, 70756020111, 69499033605, 00781176601, 00185001901, 00781280101, 16729017201, 16729017117, 59762141705, 69238105501, 00172567610, 51407035205, 51875031104, 27241017010, 00078007806, 00406990603, 00781149101, 63739035504, 00591080805, 00052010590, 43353016453, 57866389701, 00002246805, 70710122801, 62559071401, 57664069188, 61073003730, 00093730365, 59746071290, 78206015601, 50111036603, 00677119901, 60505024901, 54868488900, 55289073030, 63739018910, 51079043820, 55289001830, 00904020160, 00904126160, 54868006407, 58864002215, 00406990762, 54868222100, 00591111930, 60687028111, 42291024790, 27241021001, 55289073012, 00054467813, 00247120100, 00406991301, 00182101810, 00115165308, 00378261010, 53489033210, 51862094605, 51672421901, 29300041910, 00406990803, 60429096701, 42571034201, 60687029301, 52152034404, 60687044411, 00247081530, 60809030555, 49884021801, 52152022630, 27241016801, 76420071101, 43353061430, 51079014720, 43353053660, 00364040601, 00591578901, 00955103110, 00247061530, 00349208010, 00591111710, 52959046400, 00378312510, 64125013401, 00904697361, 51079008830, 00182133201, 54569017501, 43353037845, 54868006602, 42806080230, 00378261001, 64720041610, 00310004510, 49999021525 |
| Gabapentinoids | NDC: 55289096930, 76282056890, 00904563289, 50228017705, 69367013406, 53746010301, 43547038310, 00172444160, 71610031430, 69097081307, 63304062801, 71610021198, 71610016680, 52959074660, 43353059153, 71093011105, 71610046373, 68115050160, 00172438184, 00093762598, 45963055611, 43353087292, 43547038350, 68001041200, 76282032101, 69097067805, 52343003090, 00904666661, 67544051853, 23490651509, 00904700304, 00093762698, 42582011618, 16714050301, 43063075794, 69097094312, 43353059198, 00378542801, 68084062511, 59762413501, 43353081092, 76282032301, 52427080690, 14550051304, 69367032609, 71610040053, 43353086280, 71610033530, 43353070980, 00904710861, 68084008101, 63629259302, 62332012290, 71610035653, 71610018180, 42549053760, 33358015390, 00071080640, 61919083972, 52343003199, 69238131609, 51407051390, 43353007560, 72606000801, 00071101868, 60687048411, 68084077411, 00172438100, 68071135600, 21695027615, 55289025530, 66336043960, 43598029279, 67877046705, 55289093990, 43353087253, 42582011610, 12280027990, 43547038910, 00093762898, 71610018594, 51224001960, 64980041209, 43547033250, 53746010205, 69367013104, 65084037018, 16590010272, 16714050302, 21695005600, 43353063045, 71610014653, 43547033350, 50436728501, 69097095705, 00172444410, 72606000701, 69238131209, 63304062905, 47781038030, 50228035690, 43063049090, 63739037610, 71610031453, 50228018005, 65162010211, 00904700061, 71610006992, 14550051202, 43353063092, 43353061974, 43598029466, 43353061945, 49999078490, 63874110204, 51407004810, 62332011990, 43547026650, 00071101368, 68001000603, 58016028500, 51407004818, 00172438360, 31722016705, 43353086380, 00172438370, 42806051101, 58016029860, 71610033353, 55154358107, 60429078305, 69367013504, 63739056010, 16590050890, 43353019060, 00228286209, 67877046490, 00677193605, 60687051811, 68084056311, 00904563340, 60429073901, 76282057390, 53489028401, 43353086494, 68071029230, 00093103901, 16590010430, 64380086706, 16714033202, 43598029290, 70010010810, 42291030150, 71610046392, 69097095605, 54868549402, 42806051010, 58657062150, 51407051490, 00172444380, 14450051204, 00185009101, 68405806826, 69367032909, 51224011950, 52343003290, 69238131709, 71610036530, 58657062350, 62332012490, 53746010110, 54569563300, 65862052405, 69097068505, 72205007730, 71610041060, 43353019092, 54868549400, 71093016204, 50383031105, 00904666561, 49999053960, 63629157202, 53489028601, 71610014630, 58016036260, 35356005490, 18837019890, 64980041009, 60687049501, 53746010305, 71610018892, 00172438210, 59762505001, 63739037615, 16590010283, 67877046790, 68382020501, 55154358007, 71610031330, 64380086907, 13913000616, 71610007280, 43353084053, 76282040590, 65162010250, 71093012005, 65862020001, 62756020203, 50228035590, 57866105302, 58657062250, 55289089830, 71610012570, 63304062701, 50228018001, 49999089530, 51224012150, 00093762498, 66336043930, 00185011301, 00228285909, 52959081060, 43547033310, 65862019901, 63629470401, 67877022301, 16590050772, 43353086492, 76282032201, 00172444260, 31722061690, 00071101341, 67877022210, 60429073805, 50436126203, 59762157601, 60687050611, 67544035353, 67544035330, 66336056730, 47335069388, 00378542701, 43353008160, 76282070605, 60429073890, 43353086298, 68405006836, 68084077401, 57866105303, 00904563389, 71610031653, 62756013802, 52343003160, 68462012601, 47335068988, 62756013902, 21695005990, 42292002401, 55154358204, 43353041330, 67877046605, 00071051324, 50268035211, 71610018830, 00904563161, 58657062201, 00378542401, 58657062001, 43353061980, 59762502501, 76282056990, 43353082398, 71610016660, 51407004710, 67877046390, 51224011960, 00904682361, 00093717305, 52959089102, 60687047311, 65084037032, 51407050990, 69367032809, 76282057190, 14550051302, 31722040605, 54569870700, 69367013404, 76282022105, 69097081207, 43353037280, 71610019592, 00182271901, 43547026710, 18837008312, 71610046380, 00071040124, 43353082360, 68387066390, 00172438260, 00093762198, 63739023610, 60505255201, 31722016605, 63629457802, 00093104005, 71610040080, 42582011518, 58016031800, 58016036290, 63304062805, 00228263611, 60505011307, 00228286109, 51407004827, 72205001690, 71610006080, 51079078617, 71093012105, 68084007911, 71610018860, 62756013702, 31722022305, 43598029890, 14550051104, 67877022401, 63874110202, 55887028260, 54868522603, 68084080201, 43547039010, 70010010910, 68084056301, 71610008892, 76282032105, 43353063080, 62756020403, 69238131509, 43063044186, 71610014660, 71610032853, 60505255101, 00440755960, 69097081212, 54868545602, 52959081090, 47335068888, 60687058001, 00172438270, 66105053910, 76282070690, 71610006098, 43353070970, 50228035790, 49999089590, 68071021207, 42291030010, 00071101641, 52343003227, 47335069088, 71610033553, 68084078301, 71610021194, 68001000700, 00904699161, 67544035480, 42291030127, 68462012701, 31722040501, 42291030190, 76282022305, 00904563261, 63739039215, 00071102701, 00071080340, 71610031530, 12280007797, 00185009110, 71610007260, 59762413505, 71399055305, 71610044260, 68084076211, 51079078501, 00378542705, 69097095505, 00172438110, 59762424001, 65243030118, 71093016205, 71610046353, 54868521900, 63629336704, 00904607861, 67544051880, 00378542605, 43353019053, 69367032409, 71610008898, 00904607961, 68382020405, 43353086330, 00172444300, 63629305602, 64980041210, 71610031953, 00172444400, 52959043400, 71717010310, 12280026490, 55289057010, 43547026510, 71610035670, 61919098290, 71610007292, 63874110201, 51407004718, 67877046505, 55289089883, 16590073330, 76282040505, 00677193705, 47335068688, 71610046360, 68084008001, 00172444060, 52343003027, 71610004460, 16590050690, 65862019801, 00440755990, 52343003099, 51407004830, 68084012301, 67877022305, 43353082394, 68405068026, 49999094990, 63304059301, 42192060845, 72205007830, 43353086353, 76282032205, 63629157206, 10135064701, 00904700161, 16714050402, 71610036553, 51224002150, 53451010301, 76282062705, 54868590100, 58016013420, 63739039210, 65862052301, 65862052305, 00378542505, 68084062411, 16590050430, 60760001460, 00677193710, 65162010150, 68084008165, 65243034406, 63629306303, 71610014680, 23490732504, 43547038510, 76282057090, 50268035213, 16714066101, 58016031220, 00093762298, 43063044193, 67544035460, 43353041446, 69097068205, 68084059465, 69097081507, 67877022405, 71399055805, 00172444070, 52343003118, 51407051290, 59762502801, 51224021960, 67877022310, 50228035190, 43353061992, 60429074001, 00440756292, 71717010350, 71610008894, 00071201223, 00071101468, 54868522602, 71093011204, 71610018853, 10135064705, 68084008008, 65162010210, 42582011410, 70010010805, 67544035492, 43598029390, 71610032053, 16590050760, 62332012590, 60687047301, 00904699261, 58016031890, 43547039050, 55289021430, 51079078620, 13913000419, 16590010260, 67877046405, 42292002501, 51079078519, 68084056365, 71610013592, 54569563304, 63629305604, 71610006092, 50383031109, 58657062401, 52959050630, 51991033705, 68084008108, 16714033002, 43353087230, 51079078619, 16590010250, 43547038550, 43353073198, 00228266711, 71610035853, 76282040501, 58016029800, 43598029690, 60687060211, 55289089860, 58016029560, 72606000601, 00172444310, 71610033430, 71610040045, 00904563189, 68084059501, 43598029479, 43353026330, 55289093930, 71610019880, 52959064090, 67877046690, 68084059401, 43353026430, 60687051701, 43353059160, 54868566200, 58016031203, 68001000703, 49999053901, 71093016104, 51079078517, 71610007153, 16590050530, 69097068305, 69097081312, 68084078311, 00677193601, 71610062160, 50268035111, 54868516403, 42291030227, 00093104001, 71610012592, 67877046890, 00093717405, 49483060501, 31722022105, 00904699104, 00228285809, 63739039104, 00071201244, 76282057590, 49483060750, 68387066290, 00440755992, 60505011401, 60687051801, 58016031221, 55887033890, 71610042660, 67877046805, 00172444470, 00228266750, 69238131309, 00228263750, 16590050572, 58016032060, 63629336701, 00071101968, 00904658661, 43353059192, 59762502401, 63739037415, 53746010201, 71610019860, 55154358207, 71610033453, 55048040060, 31722022201, 00143999401, 00904563140, 50228035490, 43063027190, 43353071092, 00904700261, 68084012211, 31722022205, 68084076201, 00185009301, 00093444310, 43353086394, 60429078290, 54868522601, 69367013206, 51224002160, 31722061790, 68084008111, 47335068788, 16714066202, 59762136401, 65862019899, 71610008880, 00172444170, 31722040601, 42192060840, 43353060560, 00228266511, 43598029490, 60429073990, 71610046370, 68387041090, 63739080945, 16590010273, 00172438288, 66267033945, 60505011300, 68071021260 |
| Abbreviations: NSAIDs, non-steroidal anti-inflammatory drugs; SNRIs, serotonin-norepinephrine reuptake inhibitors; TCAs, tricyclic antidepressants.  ^1^ The algorithm for depression diagnosis was adapted from the Centers for Medicare & Medicaid Services Chronic Conditions Warehouse.  ^2^ HCPCS codes for cancer treatment was adapted from Cancer Medications Enquiry Database (CanMED) developed by the National Cancer Institute’s Division of Cancer Control and Population Sciences. | |

**Table S2: Acupuncture utilization statistics**

| **Acupuncture utilization** | **Acupuncture (N=495)** |
| --- | --- |
| Number of 30-minute sessions |  |
| Mean (SD) | 11.5 (16.9) |
| Median (Q1, Q3) | 6 (2, 14) |
| Annual expenditure (2022 USD) |  |
| Total, mean (SD) | 919.60 (1430.39) |
| Total, median (Q1, Q3) | 476.81 (176.53, 1067.16) |
| OOP, mean (SD) | 183.22 (359.11) |
| OOP, median (Q1, Q3) | 49.12 (0, 224.14) |
| Cost per 30-minute session (2022 USD) |  |
| Total, mean (SD) | 84.80 (45.50) |
| Total, median (Q1, Q3) | 78.56 (54.34, 102.54) |
| OOP, mean (SD) | 22.39 (35.69) |
| OOP, median (Q1, Q3) | 10.17 (0, 28.53) |
| Abbreviations: N, counts; OOP, out-of-pocket expenditure; Q1, quartile 1; Q3, quartile 3; SD, standard deviation; USD, United States Dollar. | |

**Table S3: Bivariate association analysis between predetermined covariates and outcomes with significant DID estimates when comparing acupuncture-treated vs non-treated breast cancer survivors**

| **Covariates** | **Opioids use** | **Gabapentinoids use** | **Gabapentinoids long-term Rx fills** |
| --- | --- | --- | --- |
|  | **Unadjusted PR**  **(95% CI)** | **Unadjusted OR**  **(95% CI)** | **Unadjusted RR**  **(95% CI)** |
| Year of index date |  |  |  |
| 2007-2009 | (ref) | (ref) | (ref) |
| 2010-2012 | 1.26*** (1.19, 1.34) | 1.59*** (1.37, 1.84) | 1.76*** (1.44, 2.15) |
| 2013-2015 | 1.40*** (1.31, 1.49) | 2.19*** (1.89, 2.55) | 2.69*** (2.19, 3.29) |
| 2016-2018 | 1.16*** (1.08, 1.24) | 2.14*** (1.83, 2.50) | 2.30*** (1.87, 2.84) |
| 2019-2021 | 1.02 (0.93, 1.13) | 2.89*** (2.39, 3.48) | 3.39*** (2.67, 4.30) |
| Index age (in years) |  |  |  |
| Quartile 1: 18-50 | (ref) | (ref) | (ref) |
| Quartile 2: 51-55 | 0.86*** (0.81, 0.91) | 1.12 (0.98, 1.29) | 1.19 (0.99, 1.42) |
| Quartile 3: 56-60 | 0.85*** (0.80, 0.90) | 1.09 (0.95, 1.24) | 1.16 (0.97, 1.39) |
| Quartile 4: 61-63 | 0.78*** (0.72, 0.83) | 1.07 (0.93, 1.25) | 1.26* (1.04, 1.53) |
| Age at breast cancer diagnosis (in years) |  |  |  |
| Quartile 1: 10-47 | (ref) | (ref) | (ref) |
| Quartile 2: 48-53 | 0.86*** (0.81, 0.92) | 1.08 (0.94, 1.23) | 1.11 (0.93, 1.34) |
| Quartile 3: 54-57 | 0.86*** (0.81, 0.92) | 1.17 (1.02, 1.35) | 1.27* (1.05, 1.53) |
| Quartile 4: 58-62 | 0.86*** (0.81, 0.92) | 1.14 (0.99, 1.31) | 1.19 (0.99, 1.44) |
| Biological sex |  |  |  |
| Female | (ref) | (ref) | (ref) |
| Male | 0.90 (0.71, 1.14) | 0.88 (0.51, 1.49) | - |
| US Region |  |  |  |
| Northeast | (ref) | (ref) | (ref) |
| Midwest | 1.06 (0.99, 1.13) | 1.32*** (1.14, 1.54) | 1.34** (1.08, 1.65) |
| South | 1.34*** (1.25, 1.43) | 1.57*** (1.35, 1.83) | 1.61*** (1.30, 1.99) |
| West | 1.08* (1.01, 1.16) | 1.17* (1.00, 1.37) | 1.15 (0.91, 1.44) |
| Payer type |  |  |  |
| Commercial | (ref) | (ref) | (ref) |
| Managed Medicaid | 1.81*** (1.65, 1.99) | 2.79*** (2.27, 3.43) | 2.85*** (2.23, 3.65) |
| Medicare Advantage or Medicare Cost | 1.82*** (1.65, 2.00) | 3.53*** (2.86, 4.36) | 3.81*** (3.02, 4.80) |
| Others | 1.00 (0.92, 1.09) | 0.92 (0.75, 1.11) | 0.82 (0.58, 1.15) |
| Plan type |  |  |  |
| PPO | (ref) | (ref) | (ref) |
| HMO | 1.15*** (1.09, 1.22) | 1.39*** (1.24, 1.56) | 1.40*** (1.20, 1.63) |
| POS | 0.87* (0.78, 0.98) | 0.70* (0.53, 0.92) | 1.04 (0.84, 1.29) |
| Others | 0.94 (0.88, 1.01) | 0.98 (0.84, 1.14) | 0.54** (0.36, 0.80) |
| Time from pre-index pain health encounter (in days) |  |  |  |
| Quartile 1: 1-116 | (ref) | (ref) | (ref) |
| Quartile 2: 118-251 | 1.05 (0.99, 1.13) | 1.17* (1.01, 1.35) | 1.17 (0.96, 1.43) |
| Quartile 3: 252-335 | 1.11** (1.04, 1.18) | 1.25** (1.09, 1.44) | 1.20 (1.00, 1.45) |
| Quartile 4: 336-365 | 1.13*** (1.06, 1.21) | 1.25** (1.08, 1.44) | 1.39*** (1.16, 1.68) |
| Pain subtypes |  |  |  |
| Musculoskeletal | 0.57*** (0.52, 0.62) | 0.57*** (0.45, 0.72) | 0.52*** (0.39, 0.68) |
| General | 2.04*** (1.92, 2.16) | 2.91*** (2.54, 3.35) | 3.03*** (2.58, 3.56) |
| Neoplasm-related | 2.40*** (2.18, 2.64) | 3.23*** (2.46, 4.24) | 3.29*** (2.46, 4.41) |
| Annual healthcare cost (2022 USD): All claims |  |  |  |
| Quartile 1 | (ref) | (ref) | (ref) |
| Quartile 2 | 1.72*** (1.57, 1.89) | 1.81*** (1.50, 2.18) | 1.97*** (1.52, 2.57) |
| Quartile 3 | 2.69*** (2.47, 2.92) | 2.93*** (2.46, 3.49) | 3.14*** (2.47, 4.00) |
| Quartile 4 | 4.43*** (4.10, 4.79) | 4.99*** (4.23, 5.89) | 5.02*** (3.99, 6.31) |
| Annual healthcare cost (2022 USD): Pain-related claims |  |  |  |
| Quartile 1 | (ref) | (ref) | (ref) |
| Quartile 2 | 1.10** (1.03, 1.19) | 1.28** (1.08, 1.51) | 1.36** (1.08, 1.71) |
| Quartile 3 | 1.29*** (1.21, 1.39) | 1.64*** (1.40, 1.92) | 1.65*** (1.34, 2.04) |
| Quartile 4 | 1.90*** (1.78, 2.02) | 2.83*** (2.44, 3.28) | 2.41*** (1.98, 2.93) |
| NCI Charlson’s comorbidity index |  |  |  |
| 0 | (ref) | (ref) | (ref) |
| 1 | 1.40*** (1.33, 1.47) | 1.72*** (1.53, 1.94) | 1.79*** (1.53, 2.10) |
| 2 | 1.72*** (1.60, 1.85) | 2.93*** (2.52, 3.41) | 3.19*** (2.63, 3.86) |
| 3 or more | 1.94*** (1.79, 2.11) | 4.12*** (3.46, 4.92) | 4.45*** (3.61, 5.48) |
| Cancer-related characteristics |  |  |  |
| Presence of metastases | 1.71*** (1.61, 1.82) | 1.55*** (1.33, 1.82) | 1.40** (1.15, 1.70) |
| Bone metastases | 2.07*** (1.92, 2.23) | 1.63*** (1.31, 2.02) | 1.49** (1.13, 1.96) |
| Brain metastases | 1.99*** (1.71, 2.31) | 1.76* (1.13, 2.72) | 1.84* (1.10, 3.08) |
| Prior taxane exposure | 1.48*** (1.41, 1.55) | 1.85*** (1.66, 2.07) | 1.88*** (1.64, 2.17) |
| Current or prior tamoxifen exposure | 0.98 (0.92, 1.05) | 0.93 (0.80, 1.07) | 0.89 (0.74, 1.08) |
| Current or prior aromatase inhibitors exposure | 1.13* (1.06, 1.21) | 1.35*** (1.19, 1.55) | 1.36** (1.14, 1.62) |
| Supportive care-related characteristics, n (%) |  |  |  |
| Current antidepressant use | 1.77*** (1.69, 1.85) | 2.47*** (2.23, 2.73) | 2.44*** (2.14, 2.78) |
| Depression | 1.59*** (1.51, 1.67) | 2.17*** (1.94, 2.42) | 2.08*** (1.80, 2.39) |
| Two or more 30-day supply of opioids prescription fills | - | 4.77*** (4.08, 5.59) | 4.93*** (4.16, 5.84) |
| Menopausal symptoms | 1.01 (0.95, 1.08) | 1.21** (1.05, 1.39) | 1.21* (1.02, 1.45) |
| Abbreviations: HMO, health maintenance organization; NCI, National Cancer Institute; OR, odds ratio; POS, point-of-service; PPO, preferred provider organization; PR, prevalence ratio; ref, reference group; RR, rate ratio; USD, United States Dollar.  *P<0.05, **P<0.01, ***P<0.001. | | | |

**Table S4: Baseline characteristics comparing acupuncture against control patients after propensity score matching**

| **Characteristics** | **Controls PSM** | **Acupuncture** | **\|SMD\|** | **P-value** |
| --- | --- | --- | --- | --- |
| N | 495 | 495 |  |  |
| Year of index date, n (%) |  |  |  | 0.18 |
| 2007 | 49 (9.9) | 58 (11.7) | 0.0301 |  |
| 2008 | 56 (11.3) | 48 (9.7) |  |  |
| 2009 | 51 (10.3) | 57 (11.5) |  |  |
| 2010 | 36 (7.3) | 28 (5.7) | 0.0574 |  |
| 2011 | 38 (7.7) | 39 (7.9) |  |  |
| 2012 | 29 (5.9) | 48 (9.7) |  |  |
| 2013 | 41 (8.3) | 32 (6.5) | 0.0202 |  |
| 2014 | 28 (5.7) | 25 (5.1) |  |  |
| 2015 | 34 (6.9) | 42 (8.5) |  |  |
| 2016 | 33 (6.7) | 40 (8.1) | 0.0549 |  |
| 2017 | 23 (4.6) | 21 (4.2) |  |  |
| 2018 | 34 (6.9) | 19 (3.8) |  |  |
| 2019 | 22 (4.4) | 27 (5.5) | 0.0379 |  |
| 2020 | 20 (4.0) | 11 (2.2) |  |  |
| 2021 | 1 (0.2) | 0 (0.0) |  |  |
| Index age, median (Q1, Q3) | 55 (51, 59) | 55 (50, 59) | <0.1^1^ | 0.68 |
| Age at breast cancer diagnosis, median (Q1, Q3) | 53 (48, 56) | 52 (47, 57) | <0.1^1^ | 0.49 |
| Female, n (%) | 490 (99.0%) | 492 (99.4%) | 0.0521 | 0.48 |
| US Region, n (%) |  |  |  | 0.96 |
| Northeast | 90 (18.2%) | 88 (17.8%) | 0.0106 |  |
| Midwest | 58 (11.7%) | 61 (12.3%) | 0.0184 |  |
| South | 25 (5.1%) | 22 (4.4%) | 0.0294 |  |
| West | 320 (64.6%) | 323 (65.3%) | 0.0127 |  |
| Payer type, n (%) |  |  |  | 0.81 |
| Commercial | 455 (91.9%) | 455 (91.9%) | 0.0000 |  |
| Managed Medicaid | 4 (0.8%) | 7 (1.4%) | 0.0513 |  |
| Medicare Advantage or Medicare Cost | 3 (0.6%) | 3 (0.6%) | 0.0000 |  |
| Others | 33 (6.7%) | 30 (6.1%) | 0.0254 |  |
| Plan type, n (%) |  |  |  | 0.71 |
| PPO | 358 (72.3%) | 366 (73.9%) | 0.0368 |  |
| HMO | 57 (11.5%) | 55 (11.1%) | 0.0129 |  |
| POS | 30 (6.1%) | 22 (4.4%) | 0.0784 |  |
| Others | 50 (10.1%) | 52 (10.5%) | 0.0132 |  |
| Time from pre-index pain health encounter (in days), median (Q1, Q3) | 236 (97, 331) | 236 (105, 328) | <0.1^1^ | 1.00 |
| Pain subtypes, n (%) |  |  |  |  |
| Musculoskeletal | 490 (99.0%) | 486 (98.2%) | 0.0605 | 0.28 |
| General | 48 (9.7%) | 46 (9.3%) | 0.0139 | 0.83 |
| Neoplasm-related | 2 (0.4%) | 5 (1.0%) | 0.0606 | 0.26 |
| Annual healthcare cost (2022 USD), median (Q1, Q3) |  |  |  |  |
| All claims | 19241.61  (9438.44, 58526.07) | 21492.51  (9591.98, 62439.71) | <0.1^1^ | 0.65 |
| Pain-related claims | 992.20  (417.06, 2257.65) | 902.03  (435.49, 2251.74) | <0.1^1^ | 0.76 |
| NCI Charlson’s comorbidity index, n (%) |  |  |  | 0.83 |
| 0 | 322 (65.1%) | 320 (64.6%) | 0.0085 |  |
| 1 | 134 (27.1%) | 128 (25.9%) | 0.0277 |  |
| 2 | 26 (5.3%) | 31 (6.3%) | 0.0417 |  |
| 3 or more | 13 (2.6%) | 16 (3.2%) | 0.0343 |  |
| Cancer-related characteristics, n (%) |  |  |  |  |
| Presence of metastases | 42 (8.5%) | 57 (11.5%) | 0.0949 | 0.49 |
| Bone metastases | 16 (3.2%) | 21 (4.2%) | 0.0501 | 0.40 |
| Brain metastases | 4 (0.8%) | 3 (0.6%) | 0.0260 | 0.70 |
| Prior taxane exposure | 124 (25.1%) | 122 (24.6%) | 0.0094 | 0.88 |
| Current or prior tamoxifen exposure | 71 (14.3%) | 75 (15.2%) | 0.0225 | 0.72 |
| Current or prior aromatase inhibitors exposure | 63 (12.7%) | 65 (13.1%) | 0.0120 | 0.85 |
| Supportive care-related characteristics, n (%) |  |  |  |  |
| Current antidepressant use | 128 (25.9%) | 131 (26.5%) | 0.0137 | 0.83 |
| Depression | 90 (18.2%) | 91 (18.4%) | 0.0052 | 0.93 |
| Two or more 30-day supply of opioids prescription fills | 14 (2.8%) | 14 (2.8%) | 0.0000 | 1.00 |
| Menopausal symptoms | 133 (26.9%) | 133 (26.9%) | 0.0000 | 1.00 |
| Abbreviations: HMO, health maintenance organization; N/n, counts; NCI, National Cancer Institute; POS, point-of-service; PPO, preferred provider organization; PSM, propensity score matching; Q1, quartile 1; Q3, quartile 3; SMD, standardized mean difference; USD, United States Dollar.  ^1^ Restricted cubic smoothing splines with five knots were used to model the relationship between each of the continuous variables and the log-odds of exposure. | | | | |

**Table S5: Baseline characteristics of high vs low acupuncture utilization patients**

| **Characteristics** | **Acupuncture,**  **<10 sessions** | **Acupuncture,**  **≥10 sessions** | **P-value** |
| --- | --- | --- | --- |
| N (%) | 317 (64.0%) | 178 (36.0%) |  |
| Year of index date, n (%) |  |  | 0.043* |
| 2007 | 37 (11.7%) | 21 (11.8%) |  |
| 2008 | 33 (10.4%) | 15 (8.4%) |  |
| 2009 | 32 (10.1%) | 25 (14.0%) |  |
| 2010 | 18 (5.7%) | 10 (5.6%) |  |
| 2011 | 31 (9.8%) | 8 (4.5%) |  |
| 2012 | 29 (9.1%) | 19 (10.7%) |  |
| 2013 | 21 (6.6%) | 11 (6.2%) |  |
| 2014 | 16 (5.0%) | 9 (5.1%) |  |
| 2015 | 26 (8.2%) | 16 (9.0%) |  |
| 2016 | 22 (6.9%) | 18 (10.1%) |  |
| 2017 | 7 (2.2%) | 14 (7.9%) |  |
| 2018 | 14 (4.4%) | 5 (2.8%) |  |
| 2019 | 21 (6.6%) | 6 (3.4%) |  |
| 2020 | 10 (3.2%) | 1 (0.6%) |  |
| Index age, median (Q1, Q3) | 55 (50, 59) | 55 (50, 59) | 0.93 |
| Age at breast cancer diagnosis, median (Q1, Q3) | 52 (47, 57) | 52 (47, 57) | 0.88 |
| Female, n (%) | 316 (99.7%) | 176 (98.9%) | 0.27 |
| US Region, n (%) |  |  | 0.076 |
| Northeast | 46 (14.5%) | 42 (23.6%) |  |
| Midwest | 40 (12.6%) | 21 (11.8%) |  |
| South | 12 (3.8%) | 10 (5.6%) |  |
| West | 218 (68.8%) | 105 (59.0%) |  |
| Payer type, n (%) |  |  | 0.10 |
| Commercial | 298 (94.0%) | 157 (88.2%) |  |
| Managed Medicaid | 4 (1.3%) | 3 (1.7%) |  |
| Medicare Advantage or Medicare Cost | 2 (0.6%) | 1 (0.6%) |  |
| Others | 13 (4.1%) | 17 (9.6%) |  |
| Plan type, n (%) |  |  | 0.35 |
| PPO | 234 (73.8%) | 132 (74.2%) |  |
| HMO | 31 (9.8%) | 24 (13.5%) |  |
| POS | 14 (4.4%) | 8 (4.5%) |  |
| Others | 38 (12.0%) | 14 (7.9%) |  |
| Time from pre-index pain health encounter (in days), median (Q1, Q3) | 232 (102, 326) | 248 (105, 333) | 0.29 |
| Pain subtypes, n (%) |  |  |  |
| Musculoskeletal | 311 (98.1%) | 175 (98.3%) | 0.87 |
| General | 31 (9.8%) | 15 (8.4%) | 0.62 |
| Neoplasm-related | 3 (0.9%) | 2 (1.1%) | 0.85 |
| Annual healthcare cost (2022 USD), median (Q1, Q3) |  |  |  |
| All claims | 18790.60  (8426.96, 48976.97) | 24879.03  (11651.11, 78318.55) | 0.010* |
| Pain-related claims | 767.17  (371.52, 2073.74) | 1194.25  (493.53, 3079.97) | 0.002** |
| NCI Charlson’s comorbidity index, n (%) |  |  | 0.88 |
| 0 | 208 (65.6%) | 112 (62.9%) |  |
| 1 | 81 (25.6%) | 47 (26.4%) |  |
| 2 | 19 (6.0%) | 12 (6.7%) |  |
| 3 or more | 9 (2.8%) | 7 (3.9%) |  |
| Cancer-related characteristics, n (%) |  |  |  |
| Presence of metastases | 37 (11.7%) | 20 (11.2%) | 0.88 |
| Bone metastases | 13 (4.1%) | 8 (4.5%) | 0.83 |
| Brain metastases | 1 (0.3%) | 2 (1.1%) | 0.27 |
| Prior taxane exposure | 65 (20.5%) | 57 (32.0%) | 0.004** |
| Current or prior tamoxifen exposure | 41 (12.9%) | 24 (13.5%) | 0.86 |
| Current or prior aromatase inhibitors exposure | 47 (14.8%) | 28 (15.7%) | 0.79 |
| Supportive care-related characteristics, n (%) |  |  |  |
| Current antidepressant use | 85 (26.8%) | 46 (25.8%) | 0.81 |
| Depression | 59 (18.6%) | 32 (18.0%) | 0.86 |
| Two or more 30-day supply of opioids prescription fills | 10 (3.2%) | 4 (2.2%) | 0.56 |
| Menopausal symptoms | 91 (28.7%) | 42 (23.6%) | 0.22 |
| Abbreviations: HMO, health maintenance organization; N/n, counts; NCI, National Cancer Institute; POS, point-of-service; PPO, preferred provider organization; Q1, quartile 1; Q3, quartile 3; USD, United States Dollar.  *P<0.05, **P<0.01. | | | |

**Table S6: Difference-in-difference exploratory analysis by high vs low acupuncture utilization**

|  | **Acupuncture, ≥10 sessions (N=178)** | | | **Acupuncture, <10 sessions (N=317)** | | | **DID**  **(ref: <10 sessions)** |
| --- | --- | --- | --- | --- | --- | --- | --- |
|  | **Pre-index** | **Post-index** | **Pre-Post Change Ratio^1^ (95% CI)** | **Pre-index** | **Post-index** | **Pre-Post Change Ratio^2^ (95% CI)** | **Ratio^3^ (95% CI)** |
| Proportion of users, n (%) |  |  |  |  |  |  |  |
| Opioids | 51 (28.7%) | 30 (16.9%) | 0.59** (0.43, 0.80) | 94 (29.7%) | 63 (19.9%) | 0.67** (0.53, 0.85) | 0.88 (0.59, 1.30) |
| NSAIDs | 46 (25.8%) | 31 (17.4%) | 0.67* (0.48, 0.95) | 57 (18.0%) | 39 (12.3%) | 0.68* (0.51, 0.91) | 0.98 (0.63, 1.55) |
| SNRIs | 15 (8.4%) | 12 (6.7%) | 0.79 (0.49, 1.26) | 32 (10.1%) | 36 (11.4%) | 1.14 (0.83, 1.57) | 0.69 (0.39, 1.22) |
| TCAs | 8 (4.5%) | 6 (3.4%) | 0.74 (0.36, 1.52) | 10 (3.2%) | 6 (1.9%) | 0.59 (0.28, 1.23) | 1.25 (0.45, 3.50) |
| Gabapentinoids | 21 (11.8%) | 24 (13.5%) | 1.17 (0.79, 1.72) | 26 (8.2%) | 41 (12.9%) | 1.66** (1.15, 2.41) | 0.70 (0.41, 1.20) |
| Short-term Rx fills, count (rate/person) |  |  |  |  |  |  |  |
| Opioids | 93 (0.52) | 90 (0.51) | 0.97 (0.53, 1.75) | 170 (0.54) | 153 (0.48) | 0.90 (0.63, 1.28) | 1.08 (0.54, 2.14) |
| NSAIDs | 42 (0.24) | 22 (0.12) | 0.52* (0.30, 0.91) | 63 (0.20) | 55 (0.17) | 0.87 (0.58, 1.32) | 0.60 (0.30, 1.20) |
| SNRIs | 2 (0.01) | 1 (0.01) | 0.50 (0.03, 8.00) | 7 (0.02) | 9 (0.03) | 1.29 (0.42, 3.91) | 0.39 (0.02, 7.73) |
| TCAs^4^ | 1 (0.01) | 0 (0.00) | - | 0 (0.00) | 1 (0.00) | - | - |
| Gabapentinoids^4^ | 0 (0.00) | 2 (0.01) | - | 10 (0.03) | 2 (0.01) | - | - |
| Long-term Rx fills, count (rate/person) |  |  |  |  |  |  |  |
| Opioids | 21 (0.12) | 25 (0.14) | 1.19 (0.33, 4.25) | 61 (0.19) | 84 (0.26) | 1.38 (0.83, 2.30) | 0.86 (0.22, 3.41) |
| NSAIDs | 60 (0.34) | 58 (0.33) | 0.97 (0.57, 1.63) | 93 (0.29) | 119 (0.38) | 1.28 (0.79, 2.08) | 0.76 (0.37, 1.54) |
| SNRIs | 56 (0.31) | 47 (0.26) | 0.84 (0.57, 1.23) | 142 (0.45) | 170 (0.54) | 1.20 (0.88, 1.63) | 0.70 (0.43, 1.14) |
| TCAs | 36 (0.20) | 16 (0.09) | 0.44 (0.18, 1.08) | 34 (0.11) | 31 (0.10) | 0.91 (0.41, 2.03) | 0.49 (0.15, 1.61) |
| Gabapentinoids | 96 (0.54) | 126 (0.71) | 1.31 (0.86, 2.01) | 90 (0.28) | 169 (0.53) | 1.88*** (1.35, 2.61) | 0.70 (0.41, 1.20) |
| Healthcare resource utilization |  |  |  |  |  |  |  |
| Total cost (2022 USD), median (Q1, Q3) | 24879 (11651, 78319) | 17634 (9056, 37089) | 0.59*** (0.46, 0.76) | 18791 (8427, 48977) | 15302 (7332, 32419) | 0.82 (0.64, 1.06) | 0.72 (0.50, 1.02) |
| Hospitalization, count (rate/person) | 41 (0.23) | 23 (0.13) | 0.56 (0.30, 1.04) | 64 (0.20) | 59 (0.19) | 0.92 (0.61, 1.38) | 0.61 (0.29, 1.28) |
| Emergency department, count (rate/person) | 98 (0.55) | 75 (0.42) | 0.77 (0.52, 1.14) | 108 (0.34) | 100 (0.32) | 0.93 (0.64, 1.33) | 0.83 (0.48, 1.41) |
| Abbreviations: CI, confidence interval; DID, difference-in-difference; GEE, generalized estimating equation; N/n, counts; NSAIDs, non-steroidal anti-inflammatory drugs; ref, reference group for DID analysis; Rx, prescription; SNRIs, serotonin-norepinephrine reuptake inhibitors; TCAs, tricyclic antidepressants; USD, United States Dollar.  ^1^ Exponential function of *β1 + β3* in the GEE-DID model, *log(Y) = β0 + β1*[post-index] + β2*[group] + β3*[post-index × group] + ε,* where *Y* are either the prevalence or odds for binary outcomes, rate outcomes for prescription fills, hospitalization and emergency department visits, or cost outcomes.  ^2^ Exponential function of *β1* in the GEE-DID model.  ^3^ Exponential function of *β3* in the GEE-DID model.  ^4^ Models did not converge if point estimate and 95% CI is not estimated.  *P<0.05, **P<0.01, ***P<0.001. | | | | | | | |

**Table S7: Baseline characteristics comparing acupuncture-treated patients for pain vs for other conditions**

| **Characteristics** | **Acupuncture for other conditions** | **Acupuncture for pain** | **P-value** |
| --- | --- | --- | --- |
| N (%) | 231 (46.7%) | 264 (53.3%) |  |
| Number of acupuncture sessions, n (%) | 5 (1.5, 12.5) | 7.5 (3, 15) | <0.001*** |
| Acupuncture total cost, median (Q1, Q3) | 385.00  (126.55, 1012.31) | 588.33  (217.70, 1143.21) | 0.004** |
| Year of index date, n (%) |  |  | 0.13 |
| 2007 | 28 (12.1%) | 30 (11.4%) |  |
| 2008 | 23 (10.0%) | 25 (9.5%) |  |
| 2009 | 19 (8.2%) | 38 (14.4%) |  |
| 2010 | 17 (7.4%) | 11 (4.2%) |  |
| 2011 | 24 (10.4%) | 15 (5.7%) |  |
| 2012 | 24 (10.4%) | 24 (9.1%) |  |
| 2013 | 11 (4.8%) | 21 (8.0%) |  |
| 2014 | 14 (6.1%) | 11 (4.2%) |  |
| 2015 | 13 (5.6%) | 29 (11.0%) |  |
| 2016 | 19 (8.2%) | 21 (8.0%) |  |
| 2017 | 10 (4.3%) | 11 (4.2%) |  |
| 2018 | 10 (4.3%) | 9 (3.4%) |  |
| 2019 | 15 (6.5%) | 12 (4.5%) |  |
| 2020 | 4 (1.7%) | 7 (2.7%) |  |
| Index age, median (Q1, Q3) | 55 (50, 60) | 55 (50, 59) | 0.98 |
| Age at breast cancer diagnosis, median (Q1, Q3) | 52 (47, 57) | 52 (47, 56) | 0.63 |
| Female, n (%) | 231 (100.0%) | 261 (98.9%) | 0.10 |
| US Region, n (%) |  |  | 0.75 |
| Northeast | 39 (16.9%) | 49 (18.6%) |  |
| Midwest | 28 (12.1%) | 33 (12.5%) |  |
| South | 12 (5.2%) | 10 (3.8%) |  |
| West | 151 (65.4%) | 172 (65.2%) |  |
| Payer type, n (%) |  |  | 0.27 |
| Commercial | 218 (94.4%) | 237 (89.8%) |  |
| Managed Medicaid | 3 (1.3%) | 4 (1.5%) |  |
| Medicare Advantage or Medicare Cost | 9 (3.9%) | 21 (8.0%) |  |
| Others | 9 (3.9%) | 21 (8.0%) |  |
| Plan type, n (%) |  |  | 0.97 |
| PPO | 169 (73.2%) | 197 (74.6%) |  |
| HMO | 27 (11.7%) | 28 (10.6%) |  |
| POS | 11 (4.8%) | 11 (4.2%) |  |
| Others | 24 (10.4%) | 28 (10.6%) |  |
| Time from pre-index pain health encounter (in days), median (Q1, Q3) | 233 (108, 332) | 237 (100.5, 326) | 0.96 |
| Pain subtypes, n (%) |  |  |  |
| Musculoskeletal | 226 (97.8%) | 260 (98.5%) | 0.59 |
| General | 20 (8.7%) | 26 (9.8%) | 0.65 |
| Neoplasm-related | 5 (2.2%) | 0 (0.0%) | 0.016* |
| Annual healthcare cost (2022 USD), median (Q1, Q3) |  |  |  |
| All claims | 21492.51  (10085.66, 70310.37) | 21468.33  (8804.27, 60037.03) | 0.42 |
| Pain-related claims | 852.43  (433.61, 2000.01) | 958.67  (436.44, 2774.64) | 0.23 |
| NCI Charlson’s comorbidity index, n (%) |  |  | 0.46 |
| 0 | 143 (61.9%) | 177 (67.0%) |  |
| 1 | 62 (26.8%) | 66 (25.0%) |  |
| 2 | 16 (6.9%) | 15 (5.7%) |  |
| 3 or more | 10 (4.3%) | 6 (2.3%) |  |
| Cancer-related characteristics, n (%) |  |  |  |
| Presence of metastases | 32 (13.9%) | 25 (9.5%) | 0.13 |
| Bone metastases | 11 (4.8%) | 10 (3.8%) | 0.59 |
| Brain metastases | 2 (0.9%) | 1 (0.4%) | 0.49 |
| Prior taxane exposure | 65 (28.1%) | 57 (21.6%) | 0.092 |
| Current or prior tamoxifen exposure | 35 (15.2%) | 40 (15.2%) | 1.00 |
| Current or prior aromatase inhibitors exposure | 34 (14.7%) | 31 (11.7%) | 0.33 |
| Supportive care-related characteristics, n (%) |  |  |  |
| Current antidepressant use | 63 (27.3%) | 68 (25.8%) | 0.70 |
| Depression | 43 (18.6%) | 48 (18.2%) | 0.90 |
| Two or more 30-day supply of opioids prescription fills | 5 (2.2%) | 9 (3.4%) | 0.40 |
| Menopausal symptoms | 61 (26.4%) | 72 (27.3%) | 0.83 |
| Abbreviations: HMO, health maintenance organization; N/n, counts; NCI, National Cancer Institute; POS, point-of-service; PPO, preferred provider organization; Q1, quartile 1; Q3, quartile 3; USD, United States Dollar.  *P<0.05, **P<0.01, ***P<0.001. | | | |

**Table S8: Difference-in-difference exploratory analysis by acupuncture for pain vs other conditions**

|  | **Acupuncture for pain**  **(N=264)** | | | **Acupuncture for other conditions (N=231)** | | | **DID**  **(ref: other conditions)** |
| --- | --- | --- | --- | --- | --- | --- | --- |
|  | **Pre-index** | **Post-index** | **Pre-Post Change Ratio^1^ (95% CI)** | **Pre-index** | **Post-index** | **Pre-Post Change Ratio^2^ (95% CI)** | **Ratio^3^ (95% CI)** |
| Proportion of users, n (%) |  |  |  |  |  |  |  |
| Opioids | 76 (28.8%) | 40 (15.2%) | 0.53*** (0.40, 0.70) | 69 (29.9%) | 53 (22.9%) | 0.77* (0.60, 0.99) | 0.69 (0.47, 1.00) |
| NSAIDs | 60 (22.7%) | 38 (14.4%) | 0.63** (0.47, 0.85) | 43 (18.6%) | 32 (13.9%) | 0.74 (0.53, 1.05) | 0.85 (0.54, 1.34) |
| SNRIs | 20 (7.6%) | 22 (8.3%) | 1.11 (0.74, 1.66) | 27 (11.7%) | 26 (11.3%) | 0.96 (0.68, 1.35) | 1.16 (0.68, 1.97) |
| TCAs | 10 (3.8%) | 10 (3.8%) | 1.00 (0.56, 1.78) | 8 (3.5%) | 2 (0.9%) | 0.24* (0.07, 0.82) | ***4.11* (1.07, 15.72)*** |
| Gabapentinoids | 20 (7.6%) | 35 (13.3%) | 1.86** (1.26, 2.75) | 27 (11.7%) | 30 (13.0%) | 1.13 (0.77, 1.64) | 1.65 (0.96, 2.84) |
| Short-term Rx fills, count (rate/person) |  |  |  |  |  |  |  |
| Opioids | 140 (0.53) | 91 (0.34) | 0.65* (0.45, 0.93) | 123 (0.53) | 152 (0.66) | 1.24 (0.78, 1.95) | ***0.53* (0.29, 0.94)*** |
| NSAIDs | 76 (0.29) | 44 (0.17) | 0.58* (0.37, 0.91) | 29 (0.13) | 33 (0.14) | 1.14 (0.70, 1.86) | ***0.51* (0.26, 0.99)*** |
| SNRIs | 3 (0.01) | 1 (0.00) | 0.33 (0.03, 3.85) | 6 (0.03) | 9 (0.04) | 1.50 (0.44, 5.14) | 0.22 (0.01, 3.44) |
| TCAs^4^ | 1 (0.00) | 1 (0.00) | - | 0 (0.00) | 0 (0.00) | - | - |
| Gabapentinoids | 5 (0.02) | 1 (0.00) | 0.20 (0.02, 2.54) | 5 (0.02) | 3 (0.01) | 0.60 (0.13, 2.79) | 0.33 (0.02, 6.50) |
| Long-term Rx fills, count (rate/person) |  |  |  |  |  |  |  |
| Opioids | 43 (0.16) | 66 (0.25) | 1.53 (0.88, 2.68) | 39 (0.17) | 43 (0.19) | 1.10 (0.48, 2.55) | 1.39 (0.51, 3.81) |
| NSAIDs | 87 (0.33) | 90 (0.34) | 1.03 (0.66, 1.62) | 66 (0.29) | 87 (0.38) | 1.32 (0.73, 2.38) | 0.78 (0.37, 1.64) |
| SNRIs | 76 (0.29) | 104 (0.39) | 1.37 (0.86, 2.17) | 122 (0.53) | 113 (0.49) | 0.93 (0.72, 1.19) | 1.48 (0.87, 2.50) |
| TCAs | 55 (0.21) | 43 (0.16) | 0.78 (0.39, 1.55) | 15 (0.06) | 4 (0.02) | 0.27 (0.06, 1.23) | 2.93 (0.55, 15.70) |
| Gabapentinoids | 72 (0.27) | 143 (0.54) | 1.99** (1.19, 3.31) | 114 (0.49) | 152 (0.66) | 1.33 (0.99, 1.80) | 1.49 (0.82, 2.69) |
| Healthcare resource utilization |  |  |  |  |  |  |  |
| Total cost (2022 USD), median (Q1, Q3) | 21468 (8804, 60037) | 14709 (7727, 29149) | 0.67*** (0.54, 0.82) | 21493 (10086, 70310) | 17275 (8173, 43146) | 0.79 (0.59, 1.06) | 0.85 (0.59, 1.21) |
| Hospitalization, count (rate/person) | 46 (0.17) | 33 (0.13) | 0.72 (0.41, 1.26) | 59 (0.26) | 49 (0.21) | 0.83 (0.54, 1.27) | 0.86 (0.43, 1.75) |
| Emergency department, count (rate/person) | 104 (0.39) | 87 (0.33) | 0.84 (0.57, 1.23) | 102 (0.44) | 88 (0.38) | 0.86 (0.59, 1.25) | 0.97 (0.57, 1.66) |
| Abbreviations: CI, confidence interval; DID, difference-in-difference; GEE, generalized estimating equation; N/n, counts; NSAIDs, non-steroidal anti-inflammatory drugs; ref, reference group for DID analysis; Rx, prescription; SNRIs, serotonin-norepinephrine reuptake inhibitors; TCAs, tricyclic antidepressants; USD, United States Dollar.  ^1^ Exponential function of *β1 + β3* in the GEE-DID model, *log(Y) = β0 + β1*[post-index] + β2*[group] + β3*[post-index × group] + ε,* where *Y* are either the prevalence or odds for binary outcomes, rate outcomes for prescription fills, hospitalization and emergency department visits, or cost outcomes.  ^2^ Exponential function of *β1* in the GEE-DID model.  ^3^ Exponential function of *β3* in the GEE-DID model.  ^4^ Models did not converge if point estimate and 95% CI is not estimated.  *P<0.05, **P<0.01, ***P<0.001. | | | | | | | |

**Table S9: Change in gabapentinoids utilization and total healthcare cost among patients treated with acupuncture for pain, stratified by high vs low acupuncture utilization**

|  | **Acupuncture, ≥10 sessions (N=106)** | **Acupuncture, <10 sessions (N=158)** | **Difference-in-difference (reference: <10 sessions)** |
| --- | --- | --- | --- |
|  | Change (95% CI) | Change (95% CI) | Change (95% CI) |
| Gabapentinoids utilization |  |  |  |
| Proportion of users, OR | 1.65 (0.99, 2.76) | 2.13* (1.17, 3.86) | 0.77 (0.35, 1.70) |
| Short-term Rx fills^1^, RR | - | - | - |
| Long-term Rx fills, RR | 1.57 (0.81, 3.05) | 2.76** (1.42, 5.36) | 0.57 (0.22, 1.46) |
| Total healthcare cost (2022 USD) |  |  |  |
| Change ratio | 0.58*** (0.43, 0.78) | 0.75* (0.57, 0.99) | 0.77 (0.51, 1.16) |
| Mean absolute change | -24377***  (-37324, -11431) | -11277*  (-21216, -1337) |  |
| Abbreviations: CI, confidence interval; DID, difference-in-difference; N, counts; NSAIDs, OR, odds ratio; RR, rate ratio; Rx, prescription; USD, United States Dollar.  ^1^ Models did not converge if point estimate and 95% CI is not estimated.  *P<0.05, **P<0.01, ***P<0.001. | | | |

**Fig. S1: Acupuncture and control cohort selection**


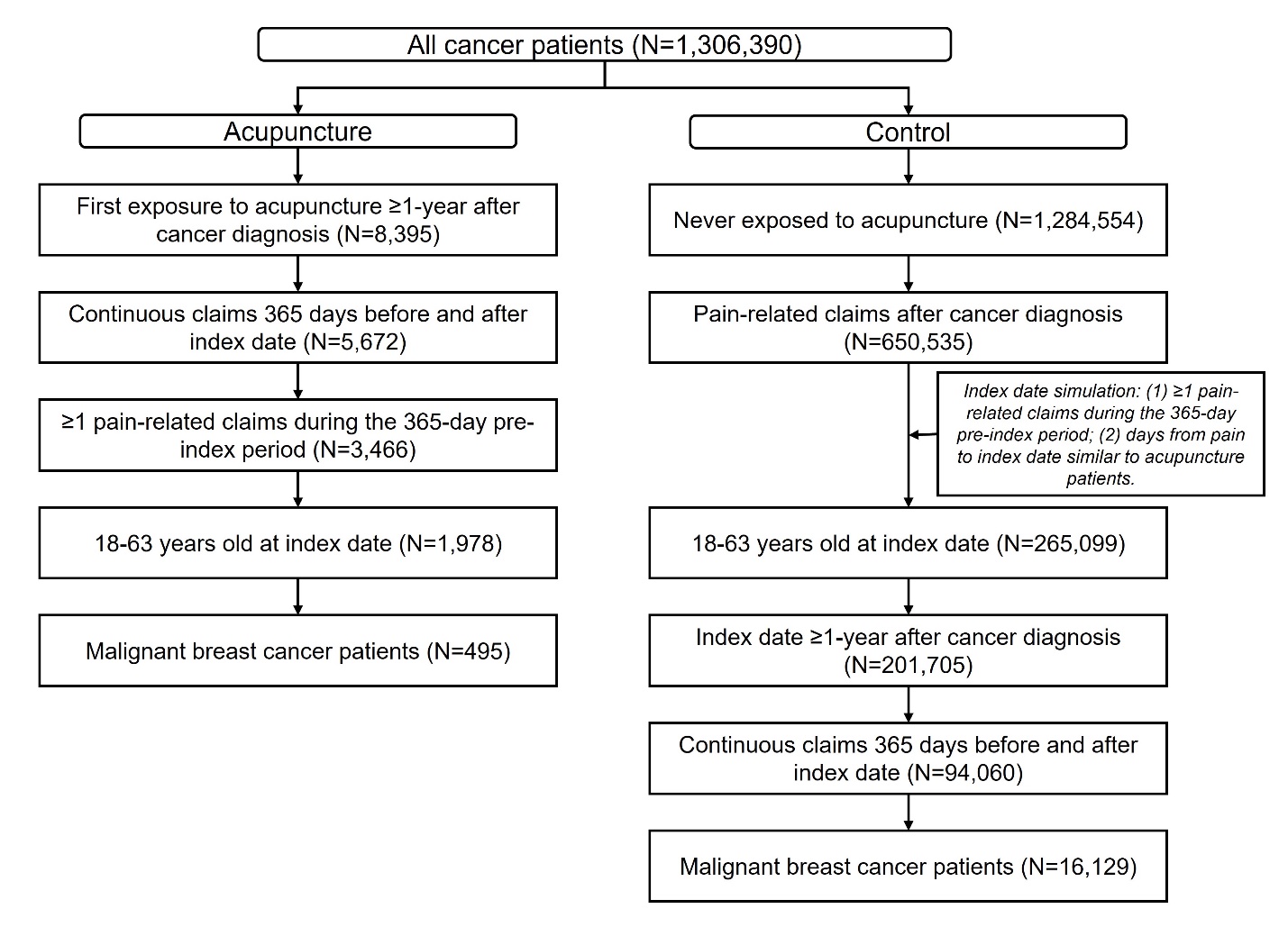


**Fig. S2: Distribution of annualized total healthcare cost, pre- and post-index**

The figure illustrates the right-skewed distribution of annualized total healthcare cost, pre- and post-index periods, for all eligible patients. Costs above $200,000 were truncated at $200,000 to make the graph easier to read.

**Fig. S3: Standardized mean differences in the unadjusted (original) and weighted (IPTW-weighted) cohorts**

**
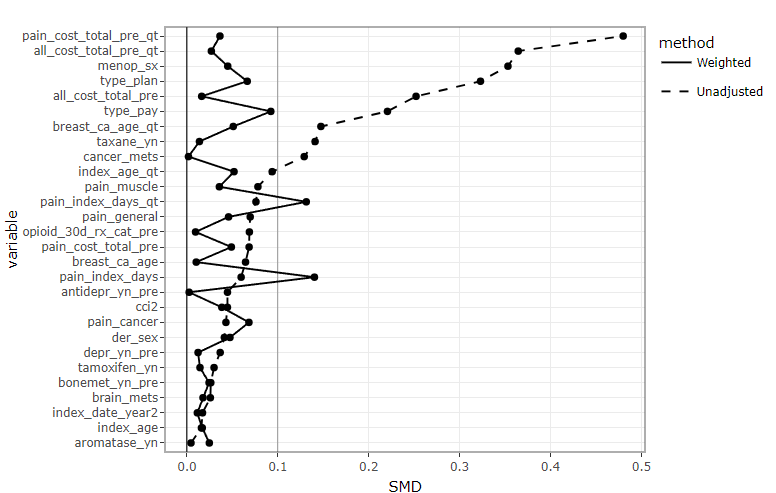
**

Abbreviations: SMD, standardized mean difference.

Time from pre-index pain health encounter could not achieve a standardized mean difference < |0.1| and was added to the generalized estimating equations, difference-in-difference models.

**Fig. S4: Effect of number of acupuncture sessions (in quintiles) on the change in total all-cause healthcare cost from pre- to post-index**


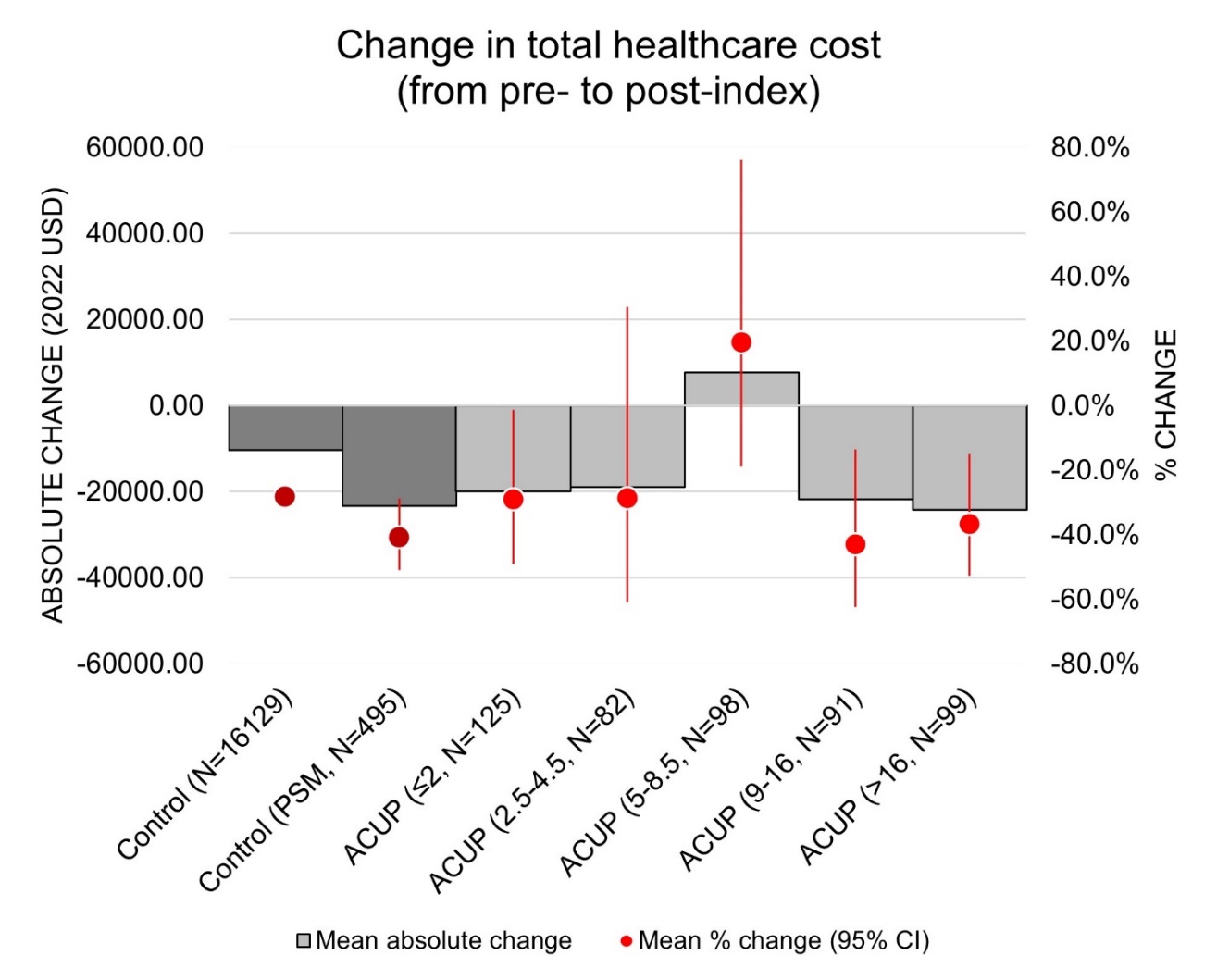

Supplement: Supplementary file 1 — Supplementary Material 1: Additional file 1: Table S1 Algorithms for identifying health-related covariates. Table S2 Acupuncture utilization statistics. Table S3 Bivariate association analysis between predetermined covariates and outcomes with significant DID estimates when comparing acupuncture-treated vs non-treated breast cancer survivors. Table S4 Baseline characteristics comparing acupuncture against control patients after propensity score matching. Table S5 Baseline characteristics of high vs low acupuncture utilization patients. Table S6 Difference-in-difference exploratory analysis by high vs low acupuncture utilization. Table S7 Baseline characteristics comparing acupuncture-treated patients for pain vs for other conditions. Table S8 Difference-in-difference exploratory analysis by acupuncture for pain vs other conditions. Table S9 Change in gabapentinoid utilization and total healthcare cost among patients treated with acupuncture for pain, stratified by high vs low acupuncture utilization. Fig. S1 Acupuncture and control cohort selection. Fig. S2 Distribution of annualized total healthcare cost, pre- and post-index. Fig. S3 Standardized mean differences in the unadjusted (original) and weighted (IPTW-weighted) cohorts. Fig. S4 Effect of number of acupuncture sessions (in quintiles) on the change in total all-cause healthcare cost from pre- to post-index. [file 12916_2024_3626_MOESM1_ESM.docx]
